# Host Colonization as a Major Evolutionary Force Favoring the Diversity and the Emergence of the Worldwide Multidrug-Resistant *Escherichia coli* ST131

**DOI:** 10.1128/mBio.01451-21

**Published:** 2021-08-24

**Authors:** Richard Bonnet, Racha Beyrouthy, Marisa Haenni, Marie-Hélène Nicolas-Chanoine, Guillaume Dalmasso, Jean-Yves Madec

**Affiliations:** a Institut National de la Santé et de la Recherche Médicale (UMR 1071), Institut National de la Recherche Agronomique (USC-2018), Université Clermont Auvergne, Clermont-Ferrand, France; b Centre National de Référence de la Résistance aux Antibiotiques, Centre Hospitalier Universitaire, Clermont-Ferrand, France; c Unité Antibiorésistance et Virulence Bactériennes, Anses Laboratoire de Lyon—Université de Lyon, Lyon, France; d Université de Paris, INSERM, IAME, Paris, France; Louis Stokes Veterans Affairs Medical Center

**Keywords:** ST131, *Escherichia coli*, population structure, extended-spectrum beta-lactamase, antibiotic resistance, virulence, veterinary microbiology, veterinary epidemiology, genome analysis, host colonization, one health, population genetics, virulence factors

## Abstract

The emergence of multidrug-resistant Escherichia coli ST131 is a major worldwide public health problem in humans. According to the “one health” approach, this study investigated animal reservoirs of ST131, their relationships with human strains, and the genetic features associated with host colonization. High-quality genomes originating from human, avian, and canine hosts were classified on the basis of their accessory gene content using pangenomic. Pangenomic clusters and subclusters were specifically and significantly associated with hosts. The functions of clustering accessory genes were mainly enriched in functions involved in DNA acquisition, interactions, and virulence (e.g., pathogenesis, response to biotic stimulus and interaction between organisms). Accordingly, networks of cooccurrent host interaction factors were significantly associated with the pangenomic clusters and the originating hosts. The avian strains exhibited a specific content in virulence factors. Rarely found in humans, they corresponded to pathovars responsible for severe human infections. An emerging subcluster significantly associated with both human and canine hosts was evidenced. This ability to significantly colonize canine hosts in addition to humans was associated with a specific content in virulence factors (VFs) and metabolic functions encoded by a new pathogenicity island in ST131 and an improved fitness that is probably involved in its emergence. Overall, VF content, unlike the determinants of antimicrobial resistance, appeared as a key actor of bacterial host adaptation. The host dimension emerges as a major driver of genetic evolution that shapes ST131 genome, enhances its diversity, and favors its dissemination.

## INTRODUCTION

Multidrug-resistant (MDR) Escherichia coli of sequence type 131 (ST131) has rapidly and globally disseminated over the last decades in human medicine, becoming the dominant MDR bacteria responsible for extraintestinal infections across the world (1–3). E. coli ST131 is therefore regarded as a major threat to human health given its high antimicrobial resistance (AMR) level and the broad spectrum of infections it causes (3).

Single nucleotide polymorphism (SNP)-based genome analysis defined three ST131 clades and subclades (4–7). They also differed by the content in accessory genes involved in AMR and virulence. The predominant subclade C2 harbor mutations in the *gyrA*/*parC* genes conferring resistance to quinolones and extended-spectrum β-lactamase (ESBL)-encoding gene *bla*_CTX-M-15_ (4–9). Initial findings on accessory virulence factors (VFs) define ST131 as extraintestinal pathogenic E. coli (2, 9–11). However, the distribution of VFs within ST131 remains poorly known, and VF content may rely more on persistence in the host rather than by conferring disease and probably results from a fine tuning of the VF content (12, 13).

Reports on E. coli ST131 agree with a much lower prevalence of ST131 outside the human sector (14). In animals, data are fragmented, and most of them report ST131 isolates from avian sources (4, 15–17) and companion animals (2, 18). Factors involved in host colonization and the role of these reservoirs in the evolutionary dynamics of ST131 lineages have not been investigated.

In this study, we showed how AMR and VF networks connected ST131 lineages to hosts, thereby providing new information on factors involved in host colonization. We also identified a emerging ST131 lineage strongly associated with the canine host, which underscores the impact of host diversity in the evolutionary dynamics of ST131.

## RESULTS

### Phylogenomic classification of genomes.

We gathered a data set of 800 high-quality ST131 E. coli genomes originating from three main hosts (avian, *n* = 140; canine, *n* = 100; human, *n* = 508) and seven geographic areas (see Table S1 in the supplemental material). Core genome SNP-based phylogeny and clade assignation (here termed phylogenomic classification) showed the clades and subclades (see Fig. S1) previously reported (4–8, 19). Interestingly, the calculation of host trait heritability (20) revealed that 75, 88, and 35% of host variance could be explained by genome variance for human, avian, and canine hosts, respectively. Our ST131 E. coli genome data set therefore contained significant information explaining host colonization.

10.1128/mBio.01451-21.1 FIG S1 (a to c) Phylogenomic classification of ST131 genomes as clades and subclades (a) and distributions among hosts distribution (%) of antibiotic resistance mechanisms among ST131 clades (b) and the main subclades (c). The significant enrichments are indicated (FDR-adjusted *P* values for Fisher tests: *, *P* ≤ 0.05; **, *P* ≤ 0.01; ***, *P* ≤ 0.001). Download FIG S1, TIF file, 0.2 MB. Copyright © 2021 Bonnet et al. 2021 Bonnet et al. https://creativecommons.org/licenses/by/4.0/ This content is distributed under the terms of the Creative Commons Attribution 4.0 International license.

10.1128/mBio.01451-21.9 TABLE S1 Genomes included in the study, associated metadata, assigned phylogenomic clades/subsclades, pangenomic clusters/subclusters, and genetic features. Avian sources, which correspond to food-producing animals (i.e., poultry) were separated from wild birds. Download Table S1, PDF file, 1.4 MB. Copyright © 2021 Bonnet et al. 2021 Bonnet et al. https://creativecommons.org/licenses/by/4.0/ This content is distributed under the terms of the Creative Commons Attribution 4.0 International license.

### Pangenomic classification of genomes.

**FIG 1:**
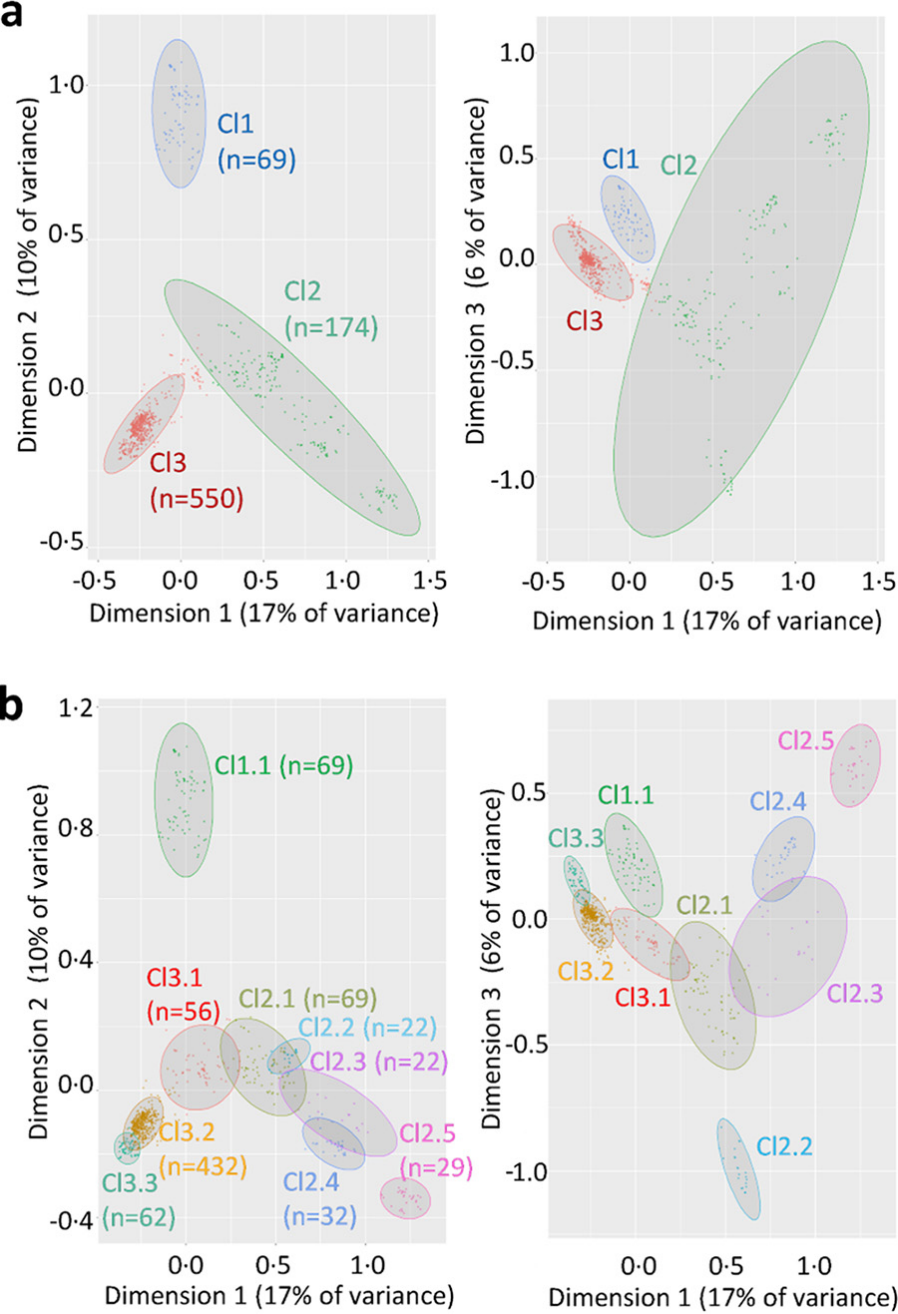
Pangenomic classification of ST131 genomes based on their accessory gene content. Clusters (a) and subclusters (b) were inferred from MCA and PAM clustering. The ellipses are 95% confidence ellipses.

**FIG 2:**
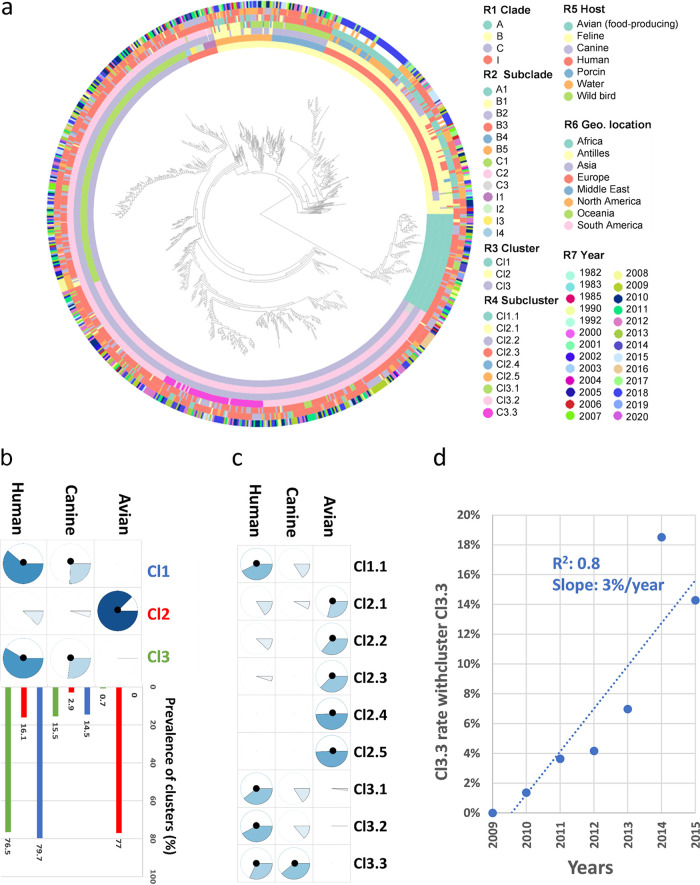
Relationships of pangenomic classifications of ST131 genomes with originating hosts. (a) Pangenomic clusters and subclsuters in the context of the SNP-based phylogenetic tree and associated data as rings (R) numbered from the inner to the outside. (b) Index of correlation (IndVal) and prevalence (%) of clusters Cl1 to Cl3 with originating hosts. (c) IndVal association index of subclusters Cl1.1 to Cl3.3 with the hosts. (d) Subcluster Cl3.3 rate among cluster Cl3 between 2008 and 2015 within canine hosts. The IndVals are reported as pie charts. The significant associations are indicated by black dots (permutation test, FDR-adjusted *P* value of <0.05). The prevalence of cluster Cl1 is reported as blue bars, that for cluster Cl2 is reported as red bars, and that for cluster Cl3 is reported as green bars.

Since the accessory genome may include crucial genes for bacterial adaptation, we conducted a pangenomic analysis of the ST131 E. coli genomes to extract accessory genes. The partitioning of ST131 genomes based on accessory genes revealed three major clusters (Cl1, CI2, and Cl3) subdivided into nine subclusters (Cl1.1, Cl2.1 to Cl2.5, and Cl3.1 to Cl3.3) (Fig. 1; see also Fig. S2) supported by Adonis tests (*P* value of <0.001). Using correlation index as a metric, clusters overlapped with clades at 91%, whereas subclusters only overlapped with subclades at 61% (Fig. 2a). Subcluster Cl3.3 formed a new entity, including human and canine strains harbored by a phylogenetic branch of subclade C2. Subcluster Cl3.2 included the predominant clades C1 and C2 (except strains Cl3.3), subclade B4, and strains from B5. Cluster Cl2 corresponded to most clade B and corresponding subclusters except B4 and B5 strains. Cluster 1 and subcluster 1.1 corresponded to clade A. Table S2 summarizes subcluster-subclade relationships and key features of both classifications. The pangenomic classification was globally consistent with the phylogenetic tree but provided a novel classification related to accessory gene distribution.

10.1128/mBio.01451-21.2 FIG S2 Pearson’s Phi association coefficients of accessory genes with clusters (c) and subcluster (d) reported as heatmaps. The red-blue gradient indicates positive and negative correlations. Download FIG S2, TIF file, 0.2 MB. Copyright © 2021 Bonnet et al. 2021 Bonnet et al. https://creativecommons.org/licenses/by/4.0/ This content is distributed under the terms of the Creative Commons Attribution 4.0 International license.

10.1128/mBio.01451-21.10 TABLE S2 Cluster/subcluster features with the frequency of subclades, serogroups, *fimH* alleles, major hosts, discriminant VFs, and resistance mechanisms. The key values are indicated in boldface. Download Table S2, PDF file, 0.1 MB. Copyright © 2021 Bonnet et al. 2021 Bonnet et al. https://creativecommons.org/licenses/by/4.0/ This content is distributed under the terms of the Creative Commons Attribution 4.0 International license.

### Host and pangenomic classification.

The ST131 hosts were linked to the pangenomic clusters by a Cramer’s correlation index of 60% and of only 42% for the clades. We therefore investigated the significant links (false discovery rate [FDR]-adjusted *P* value of <0.05) between hosts, clusters, and subclusters (Fig. 2b to d). The avian source was significantly associated with cluster Cl2 and corresponding subclusters (Fig. 2b and c), which gathered 96% of avian strains. A total of 77% of the Cl2 strains were from avian hosts, while only 16% were from human hosts. Conversely, human host was associated with the other clusters Cl1 and Cl3 (Fig. 2b), which combined >90% of strains isolated from humans. Subclusters Cl1.1, Cl3.1, and Cl3.2 and, to a lesser extent, Cl3.3 were significantly associated with human hosts (Fig. 2c). The canine hosts exhibited a similar distribution among the clusters and clusters, suggesting a cross-transmission between human and dogs. However, Cl3.3 was the only subcluster significantly associated with canine hosts, showing a significant enrichment in canine hosts within subcluster Cl3.3 (Fig. 2c). Cl3.3 was undetected before 2010, and its rate increased (∼3%/year) among the Cl3 strains between 2010 and 2015 (Fig. 2d), suggesting that these strains, isolated in Europe, Asia, and North America, were emerging among ST131. Overall, there was not a strict specificity of clusters or subclusters for host, but there were significant and preferential associations.

### Distribution of VFs among clusters and subclusters.

**FIG 3:**
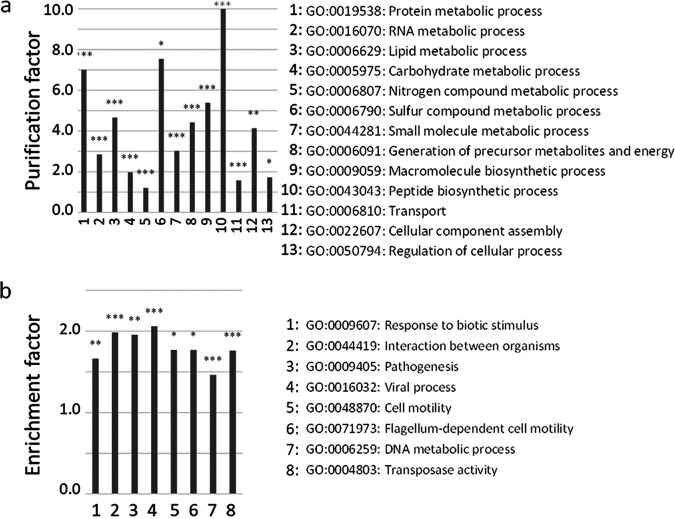
(a and b) Significantly purified (a) and enriched (b) GO terms among the GO annotations of 9,407 accessory genes significantly involved in the clustering of ST131 strains. The enrichment factors are the ratio of GO term frequencies in the clustering genes and in the whole pangenome. Inversely, the purification factors are the ratio of GO term frequencies in the whole pangenome and in the clustering genes. GO IDs are indicated with the corresponding definitions. The enrichment and the purification in GO terms were assessed by Fisher tests. The *P* values were adjusted for multiple testing by the FDR procedure (variable significance: *, *P* ≤ 0.05; **, *P* ≤ 0.01; ***, *P* ≤ 0.001).

**FIG 4:**
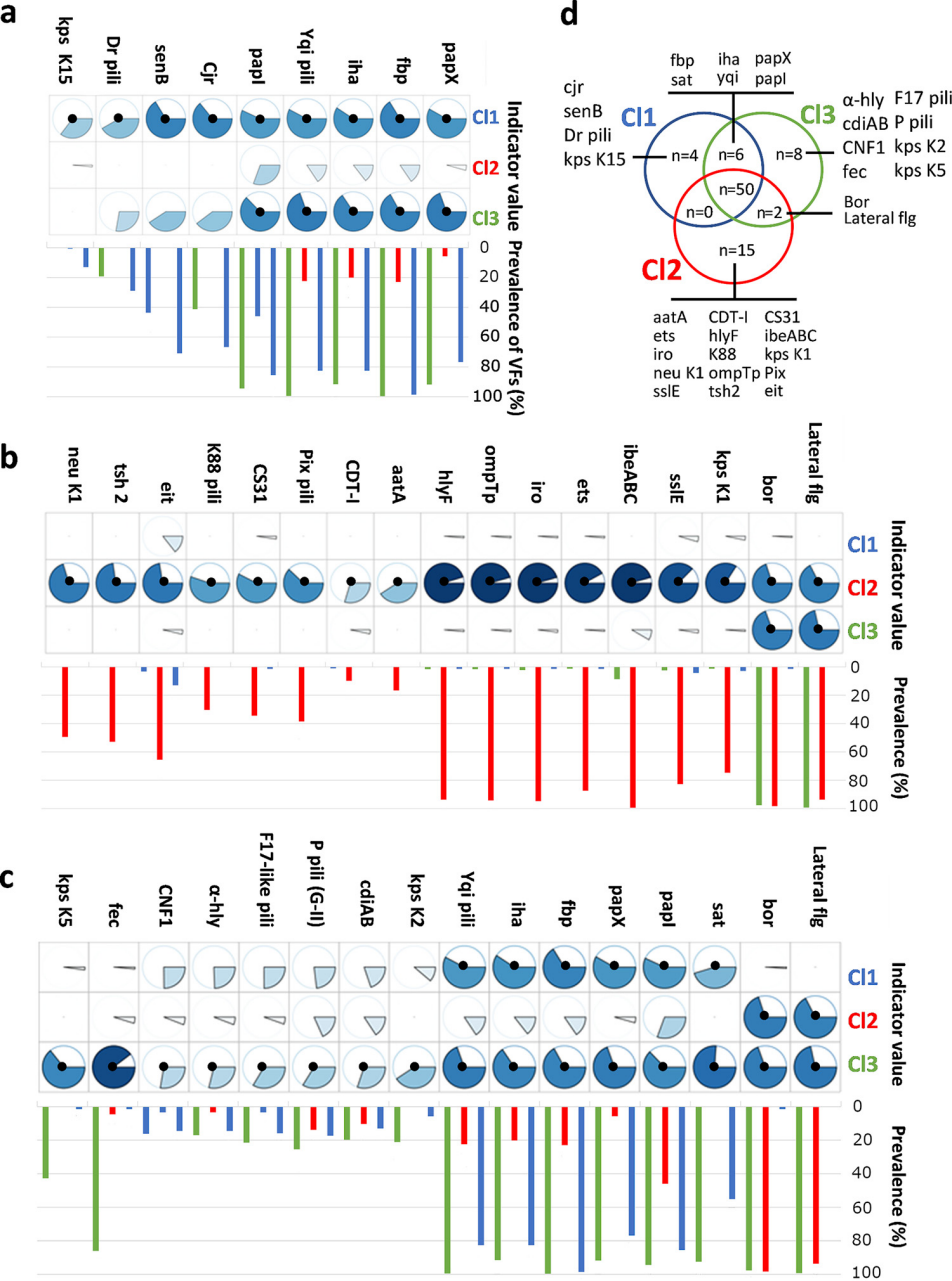
VFs were significantly associated with the pangenomic clusters using IndVal as an association metric. (a) VFs preferentially associated with cluster Cl1; (b) VFs preferentially associated with cluster Cl2; (c) VFs preferentially associated with Cl3 and (d) Venn’s diagram reporting VFs significantly associated with the clusters and their combinations. The IndVals are reported as pie charts, and the significant values (adjusted *P* value of ≤ 0.01) are indicated by a black dot. The significance was assessed by permutation tests, and the *P* values were adjusted for multiple testing by the FDR procedure. The prevalence of VFs within each cluster is indicated as a bar plot (Cl1 in blue, Cl2 in red, and Cl3 in green).

A total of 9,407 genes were involved in the clustering of ST131 genomes (permutation test, FDR-adjusted *P* value of ≤0.05; see Fig. S3). The functions significantly purified from these clustering genes were housekeeping functions (Fig. 3a). Enriched GO terms corresponded to functions involved in virulence and host interactions (Fig. 3b). We therefore investigated the distribution of VFs among the clusters and subclusters to explain their ability to colonize different hosts. The resulting binary matrix revealed 32 persistent VFs and VFs significantly associated with the pangenomic clusters (Fig. 4).

10.1128/mBio.01451-21.3 FIG S3 The 9,407 clustering accessory genes of ST131 strains presented as a Venn diagram. Download FIG S3, TIF file, 0.1 MB. Copyright © 2021 Bonnet et al. 2021 Bonnet et al. https://creativecommons.org/licenses/by/4.0/ This content is distributed under the terms of the Creative Commons Attribution 4.0 International license.

Fifteen VFs were preferentially associated with cluster Cl2 strains, which exhibited the most distinct VF profile. Seven VFs had both a sensitivity and specificity of association higher than 70% and are well-known VFs of avian pathogenic E. coli (APEC) (21). The association of APEC VFs with cluster Cl2 strains agreed with their preferential association with avian hosts and their rare isolation from humans. Cluster Cl1 differed by significant associations to four VFs, and cluster Cl3 differed by significant associations to eight VFs (Fig. 4). However, strains of cluster Cl1 and Cl3 shared six significantly associated VFs that can explain their similar affinity for humans.

**FIG 5:**
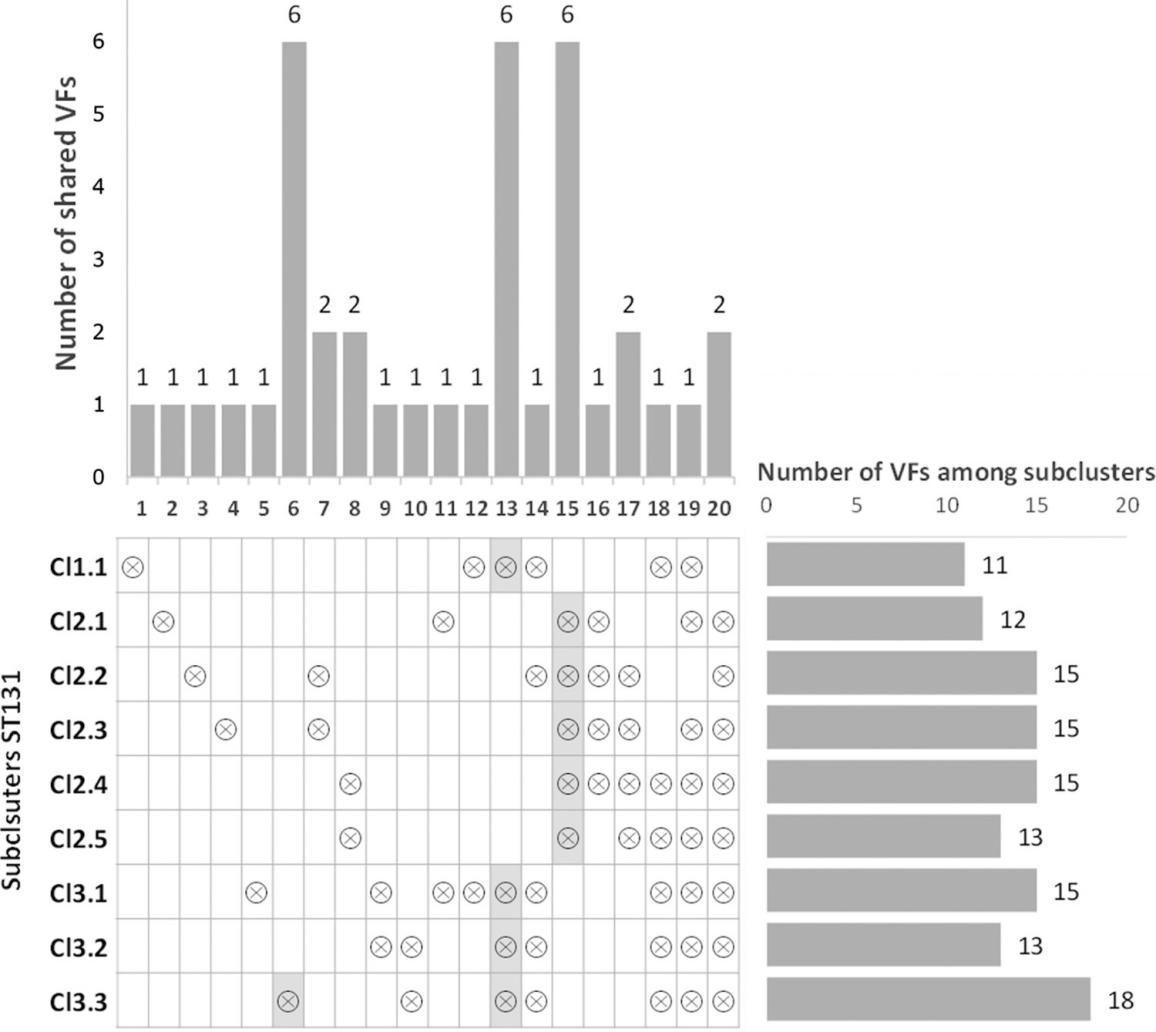
Distribution of VFs among the subclusters (see Fig. S4). The horizontal graph reports the total number of VFs significantly associated with each subcluster. The vertical graph reports the distribution of VF numbers that were significantly associated with a subcluster or the combination of subclusters as indicated in the table by “⊗” symbols. Shaded cells in the table correspond to subclusters and combinations of subclusters that were significantly associated with preferential hosts (line 6, subcluster 3.3 associated with canine hosts; line 13, subclusters Cl1.1, Cl3.1, Cl3.2, and, Cl3.3 associated with human hosts; and line 15, subclusters Cl2.1 to Cl2.5 associated with avian hosts). The IndVal index was used as an association metric of VFs with the subclusters. The statistical significance of associations was assessed by permutation tests. The resulting *P* values were adjusted for multiple testing by the FDR procedure (statistical significance, adjusted *P* ≤ 0.01).

The number of VFs significantly associated with the subclusters (see Fig. S4) varied between 11 and 18; the richest subcluster being subcluster Cl3.3 (Fig. 5). The count of VFs shared by the subclusters uncovered a high number of VFs accumulated for the subcluster Cl3.3, which was identified as a main cluster of canine strains, and for the combinations of subclusters associated with avian and human hosts (Fig. 5). The consistent relationships between VF distributions among subclusters and hosts suggests a host-associated selective pressure shaping VF content.

10.1128/mBio.01451-21.4 FIG S4 VFs significantly associated with the pangenomic subclusters using IndVal as an association metric. The IndVals are reported as pie charts and the significant values (adjusted *P* value of ≤0.01) are indicated by a black dot. The significance was assessed by permutation tests, and the *P* values were adjusted for multiple testing by the FDR procedure. Download FIG S4, TIF file, 0.2 MB. Copyright © 2021 Bonnet et al. 2021 Bonnet et al. https://creativecommons.org/licenses/by/4.0/ This content is distributed under the terms of the Creative Commons Attribution 4.0 International license.

### Networks of VFs among clusters and subclusters.

**FIG 6:**
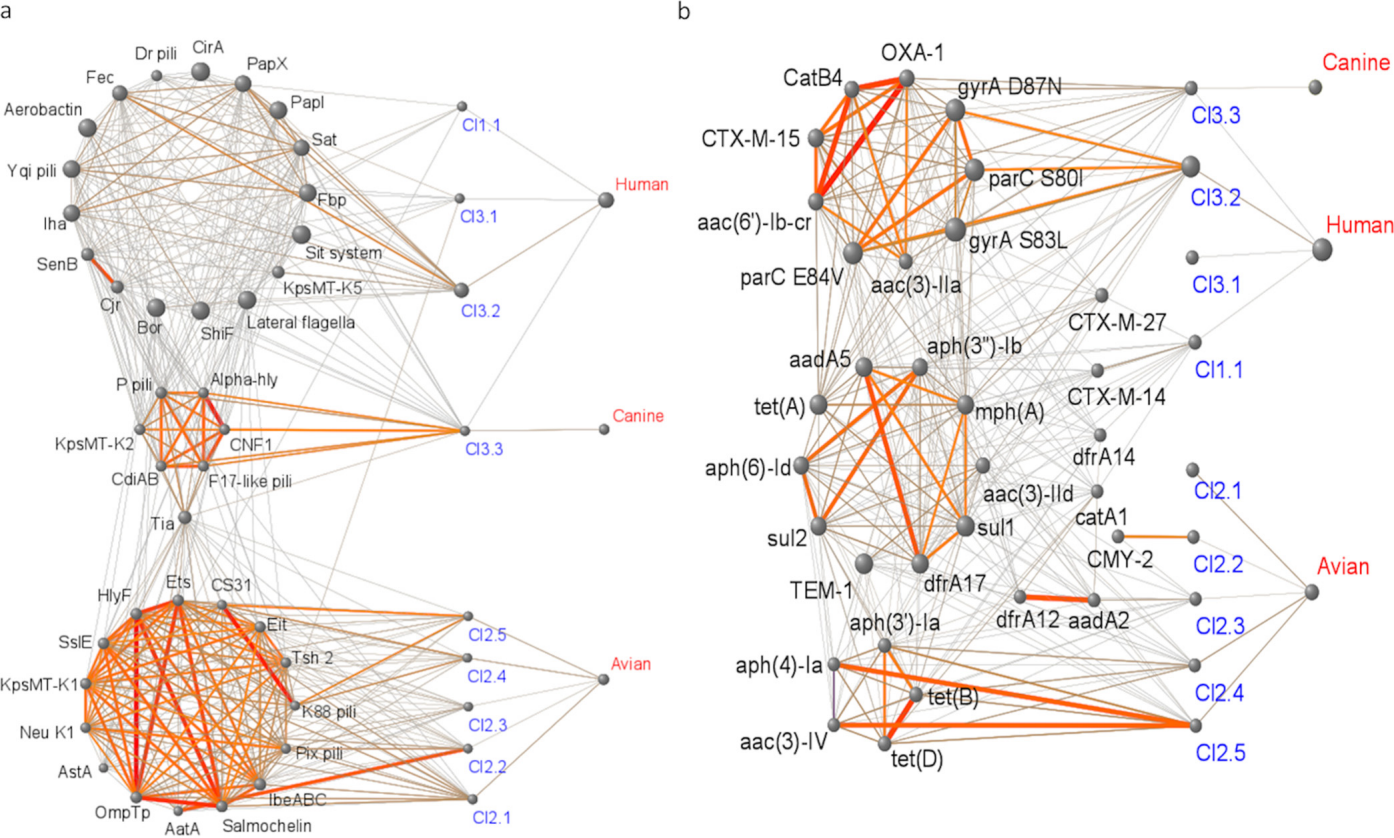
(a and b) Network analysis of VF (a) and AMR (b) distribution among ST131 subclusters and the originating hosts. Nodes represent VFs (annotation in black), subclusters (annotation in blue), and hosts (annotation in red) and link the significant cooccurrence (adjusted *P* values of ≤0.01). The cooccurrence was inferred from Jaccard index. Node that the diameter is scaled to the frequency and that the link width and color (gray-yellow-red gradient) are scaled to the Jaccard index value. The statistical significance of correlations was assessed from 1,000 bootstrap replicates, and *P* values were adjusted for multiple testing by the FDR procedure.

The combinations of VFs significantly associated with subclusters and hosts (permutation test, FDR-adjusted *P* value of ≤0.05) using a network approach based on Jaccard’s correlation coefficient (Fig. 6a). Three VF networks emerged from the data. The first network showed a high density of strong and significant connections within VFs and with all Cl2 subclusters, which were also strongly and similarly linked to the avian source. Conversely, it was scarcely connected to the other VF networks, subclusters, and hosts, showing that strains Cl2 clearly diverged by VF content. The second VF network was a weakly structured and connected to subclusters Cl1.1, Cl3.1, and Cl3.2 to Cl3.3, which were linked to human host. The subcluster Cl3.2, which was the strongest subcluster connected to the VF network, was also the subcluster most strongly linked to the human source, suggesting that the presence of the VF network enhances human colonization. The third network was densely structured and only connected to subcluster Cl3.3. Cl3.3 shared therefore links with two VF networks, showing that the Cl3.3 strains accumulate VFs that could explain its association with both human and canine hosts.

The genes included in the VF network specific to Cl3.3 were localized on the same contig and constituted a pathogenicity island (PAI) (see Fig. S5a). In addition to VF genes, the PAI-encoded genes involved in fermentative and ethanolamine metabolisms, which are important for bacterial growth and gut colonization (12, 22). Accordingly, the growth of Cl3.3 strains provided higher CFU rates than Cl3.2 strains after overnight incubation at 37°C in Luria-Bertani broth (see Fig. S5b). In a conventional mouse model, where the animals were orally infected with 10^8^ CFU, Cl3.3 isolates were recovered at higher rates from the feces than were Cl3.2 isolates (see Fig. S5c). Cl3.3 thus has specific features that improve its fitness and could favor its emergence.

10.1128/mBio.01451-21.5 FIG S5 (a to c) Pathogenicity island (128.9 kb) inserted in tRNA gene *pheU* of subcluster Cl3.3 strains ST131 (a) and the ability of these strains Cl3.3 (*n* = 10) to grow in Luria-Bertani broth overnight at 37°C (b) and to colonize the guts of mice (c) in comparison to strains Cl3.2 devoid the PAI (*n* = 10). The PAI insertion site *pheU* and the resulting duplication of its 3′ end are indicated in red. The virulence genes are annotated and zones encoding functions involved in gut colonization (i.e., fermentation and ethanolamine metabolism). Gut colonization was challenged in a mouse model by oral gavage with 10^8^ CFU of E. coli. The numbers of E. coli CFU were determined in feces collected daily postinoculation by plating on selective medium. The significant differences were assessed by the *t* test after pretesting for data normality using the Shapiro-Wilks test. (significance: *, *P* ≤ 0.05; **, *P* ≤ 0.01). Download FIG S5, TIF file, 0.1 MB. Copyright © 2021 Bonnet et al. 2021 Bonnet et al. https://creativecommons.org/licenses/by/4.0/ This content is distributed under the terms of the Creative Commons Attribution 4.0 International license.

### Distribution of AMR genes among ST131 clusters and subclusters.

AMR genes were significantly associated with one or two ST131 clusters (see Fig. S6). Cl2 did not share AMR markers with the other clusters, and the prevalence of AMR genes was overall lower in this cluster than in the others. The most prevalent AMR genes were the *tet*(B) and *tet*(D), which target antibiotics frequently used in veterinary medicine. In contrast to Cl2, Cl1 and (mainly) Cl3 strains accumulating AMR genes target antibiotics that are critical and widely used in human medicine (see Fig. S6). The numbers of AMR genes significantly associated with subclusters were heterogeneous and varied between 0 and 16 (see Fig. S7 and S8 in the supplemental material). The co-occurrence of AMR genes and their associations to subclusters and hosts revealed three AMR networks (Fig. S6b). Two were well structured by dense and strong links; one was significantly linked to Cl3.2 and Cl3.3, and the second was significantly linked to Cl2.4 and Cl2.5. However, all subclusters were not linked to AMR networks.

10.1128/mBio.01451-21.6 FIG S6 AMRs were significantly associated with the pangenomic clusters using IndVal as an association metric. (a) AMRs preferentially associated with cluster Cl1; (b) AMRs preferentially associated with cluster Cl2; (c) AMRs preferentially associated with Cl3 and (d) Venn diagram reporting AMRs significatively associated with the clusters and their combinations. The IndVals are reported as pie charts, and the significant values (adjusted *P* value of ≤0.01) are indicated by a black dot. The significance was assessed by permutation tests, and the *P* values were adjusted for multiple testing by the FDR procedure. The prevalence of VFs within each cluster is indicated as a bar plot (Cl1 in blue, Cl2 in red, and Cl3 in green). Download FIG S6, TIF file, 0.2 MB. Copyright © 2021 Bonnet et al. 2021 Bonnet et al. https://creativecommons.org/licenses/by/4.0/ This content is distributed under the terms of the Creative Commons Attribution 4.0 International license.

10.1128/mBio.01451-21.7 FIG S7 AMRs significantly associated with the pangenomic subclusters using IndVal as an association metric. The IndVals are reported as pie charts and the significant values (adjusted *P* value of ≤0.01) are indicated by a black dot. The significance was assessed by permutation tests, and the *P* values were adjusted for multiple testing by the FDR procedure. Download FIG S7, TIF file, 0.2 MB. Copyright © 2021 Bonnet et al. 2021 Bonnet et al. https://creativecommons.org/licenses/by/4.0/ This content is distributed under the terms of the Creative Commons Attribution 4.0 International license.

10.1128/mBio.01451-21.8 FIG S8 Distribution of ARMs among the subclusters. The horizontal graph reports the total number of ARMs significantly associated with each subcluster. The vertical graph reports the distribution of ARM numbers that were significantly associated with a subcluster or a combination of subclusters, as indicated in the subcluster table. The IndVal index was used as an association metric. The statistical significance was assessed by permutation tests. The resulting *P* values were adjusted for multiple testing by the FDR procedure (statistical significance, adjusted *P* value of ≤0.01). Download FIG S8, TIF file, 0.08 MB. Copyright © 2021 Bonnet et al. 2021 Bonnet et al. https://creativecommons.org/licenses/by/4.0/ This content is distributed under the terms of the Creative Commons Attribution 4.0 International license.

## DISCUSSION

Until now, there has been no indication that the evolutionary dynamics of ST131 reflect an independent and host-specific adaptation of this lineage outside humans. In contrast, the limited number of ST131 reports in animals support the common view that it rather reflects a spillover of the human sector. This study uncovered a link between host, ST131 population structure, and VF content. Host colonization requires hijacking host colonization resistance systems, which differ among hosts (23). The changes in VF prevalence among hosts may reflect adaptations to these constraints, since the primary role of VFs is to establish commensal relationships with the host rather than virulence *per se* (24).

The strains preferentially hosted by humans were linked to a sparse VF network, showing less need for specific VFs in human colonization. Accordingly, human strains were observed in all clades and clusters. Previous studies have established humans as the preferential host of ST131 (2), and humans are indeed the preferential host of clade A ST131 strains, which are rooting the ST131 phylogenetic tree (4–6, 8). Human colonization is therefore an old and intrinsic feature of ST131. However, the density and the strength of connections between VFs and the clusters strongly associated with humans (Cl3.2) increased with the association intensity of clusters with human hosts, suggesting that accessory VFs enhance human colonization and can explain the ecological success of subcluster Cl3.2 (MDR subclades C1 and C2) in humans.

Within the core-genomic clade B, previously identified as a foodborne uropathogen (16), we identified subclusters preferentially associated with avian hosts, essentially poultries. These subclusters shared a specific VF network, suggesting that the avian ecological niche generates selective pressure to maintain the coexistence of specific VFs. These VFs have been previously observed in avian pathogenic E. coli and E. coli strains responsible for severe human infections such as sepsis and neonatal meningitidis (21). These pathovars, responsible for systemic infections in avian species and more rarely in humans, are similar and two faces of the same pathovar (21). Human invasive infections by ST131 may be therefore a coincidental by-product of ST131 adaptation to avian habitat. The results also show that avian hosts are the preferred reservoir for these invasive strains and humans and that an occasional host that could be infected through contacts with avian reservoirs.

A novel subcluster, Cl3.3, was significantly associated with canine hosts. The prevalence of these strains increased rapidly in our data set between 2008 and 2015 in humans and even more in canine hosts. Most strains were collected from Europe (71%, *n* = 44) and France (53%, *n* = 33), so further studies are needed to assess whether there is truly a heterogeneous prevalence among geographic areas or whether this overrepresentation is specific to our collection. Although Cl3.3 strains are closely related to the human subcluster Cl3.2 (both belong to clade C2), they contained a specific PAI in the ST131 population. This PAI is related to those reported in adherent-invasive E. coli, which are highly competitive E. coli in the context of the gut (21). Preliminary results on the *in vitro* growth, as well as the *in vivo* survival, in the digestive tracts of mice showed that the Cl3.3 isolates reached higher CFU rates and survived better in mice than Cl3.2 isolates. This PAI may therefore enhance host colonization and explain Cl3.3 emergence. The constraints resulting from canine host colonization may have been an evolutionary force that shaped the emergence of this new lineage in ST131 and tied it to the ST131 population structure.

The heritability suggested that bacterial genomes contained significant information explaining dog colonization. Accordingly, the genotype corresponding to subcluster 3.3 was enriched in canine hosts. However, this heritability was lower than for humans and avian hosts, showing that other major factors, independent of the genome, may be involved in dog colonization. Except for the overrepresentation of subcluster 3.3, the distribution of subclusters in canine hosts was closely related to that in humans, in agreement with previously reported data (25). An increase in multiresistant E. coli genotypes was also demonstrated for farmers and employees in direct contact with broilers (26). Close and direct contacts with animals, especially with pets, probably favor human-animal cross transmissions. The impact of such transmissions may be limited for the avian hosts, which are food-producing animals living at distance of most humans, but can explain the similarity in ST131 subclusters that we observed in humans and canine hosts.

Distinct cooccurring AMR networks were also preferentially associated with different ST131 hosts. However, unlike VF networks, they appear to be not required for host colonization, since they are absent from several ST131 clusters. This observation agrees with a previous study stating that AMR is not a major driver of ST131 ecological success (9). AMR genes targeting molecules widely used in humans, such as fluoroquinolones and third-generation cephalosporins, were preferentially observed in strains originating from human hosts. In contrast, AMR genes targeting molecules widely used in veterinary medicine, such as cyclins, were preferentially observed in strains originating from avian hosts. AMR profiles are therefore probably the consequence of host-associated antibiotic selective pressure. Since VFs appear as major factors of host colonization, AMRs may therefore emerge in hosts secondarily as a response to antibiotics administered to hosts. They appear as second-line adaptation factors and can consequently enhance the colonization of host in the presence of antibiotics.

Overall, avian ST131 strains formed a distinct cluster rarely found in humans but responsible for severe infections. Conversely, humans were the main reservoir of the other clusters and the probable source of emerging strains frequently observed in dogs, exhibiting a specific VFs and an improved fitness. This study helps us to better understand the reservoir of ST131, the putative transmission flux, associated risks, and the evolutionary dynamics of this bacterial population and highlights a paradigm in which host colonization stands as a key ecological force of the ST131 evolution.

## MATERIALS AND METHODS

### Genomic data set.

The genome data set was generated from previously published data (4–6, 8, 27), Enterobase (https://enterobase.warwick.ac.uk), and 200 E. coli strains ST131 isolated from humans (*n* = 138) and animals (*n* = 62) that were sequenced for this study. It includes clinical and fecal isolates from both humans and animals. Strain names and metadata are reported in Table S1.

### DNA sequencing.

Genomic DNA was extracted using a QIAxtractor (Qiagen, Valencia, CA), and library preparation was performed by using a Nextera XT DNA sample preparation kit (Illumina, San Diego, CA) according to the Illumina protocol. The libraries were sequenced with the Illumina NextSeq500 platform (150-bp paired-end reads) at an average depth of mapped reads of 132.8 ± 60.9 (mean ± the standard deviation) with a minimum of 46-fold and an average breadth of coverage of 94.4% ± 2.6% using the EC958 genome as reference (6, 28). The sequences were submitted to ENA (BioProject PRJEB42322).

### Genome assembly and quality control.

Contaminant searches were performed for each sample using centrifuge (29). Quality control metrics were examined across the whole collection as a batch report to ensure a mean read base-pair quality score of Q ≥ 20 and a read length of 70% of the original read length. Quality-filtered Illumina reads were assembled using Unicycler (30). Contigs were annotated using Prokka (31).

Low-quality genomes were excluded as previously described (6). Genomes were excluded based on the sequence coverage, the number of unmapped bases (in the mapping reference EC958 genome), the number of uncalled bases due to low coverage or mixed-base calls, the number of scaffolds, and the estimated genome size and the numbers of open reading frames, as previously described (6). Consequently, the genome selected from Illumina short reads assemblies (*n* = 796) had *N*_50_ values of 185,053 ± 77,812 bp (mean ± the standard deviation), lengths of 5,191,763 ± 142,233 bp, 133 ± 92 contigs, and 4,880 ± 160 genes. The three PacBio assemblies and the complete genome (NC_013654) had one to six contigs with an *N*_50_ of 4,925,610 ± 181,939 bp, were 5,079,569 ± 294,615 bp long, and contained 4,908 ± 429 genes.

### Gene annotation.

A total of 17,157 orthologous genes, including 4,146 persistent and 13,011 accessory genes, were identified from the pangenomic analysis. They were annotated with the gene ontology functional enrichment annotation tool (32). To define the presence of specific genes and their alleles, we used ARIBA (33), DIAMOND (34), and the following databases: MLST database, serotypeFinder O:H typing database, the *fimH* typing database, and curated databases of VF genes and AMR genes/SNPs, including VFDB, Res-Finder, NDARO, and CARD. A total of 621 virulence genes were identified and clustered as 96 VFs.

### Comparative genomic analysis.

The genome assemblies were aligned against the reference strain EC958 (6) to call SNPs using BactSNP (35). A maximum-likelihood phylogeny was then generated by RAxML-ng v0.9 (36) based on the resulting core genome alignment filtered for recombination using Gubbins v2.2 (37). Clade and subclade classifications were assigned as previously reported (4–8, 19). The pangenome was created with PPanGGOLiN (38).

### Intestinal colonization.

The animal protocols were approved by the French Ministry of Education, Research and Innovation (permit numbers 16354 and 22507), and all animals were used in accordance with European Community guidelines (86/609/CEE). Experiments were performed using 6- to 8-week-old C57/BL6 male mice (Charles River) in a specific-pathogen-free animal facility at the University of Clermont Auvergne. They received oral gavage with a 200-μl suspension containing 10^8^ CFU of Cl3.3 (*n* = 10) or Cl3.2 (*n* = 10) E. coli producing ESBL CTX-M-15. Fecal samples were collected, diluted in sterile phosphate-buffered saline, and then plated on the selective chromogenic medium CHROMID ESBL (bioMérieux) to quantify the number of these bacteria in gut. No cefotaxime-resistant bacteria were found in noninfected mice, and the identification of E. coli isolates was confirmed by mass spectrometry using the Vitek MS system (bioMérieux).

### Pangenomic clustering.

Multiple correspondence analysis (MCA) was performed to convert the matrix reporting accessory gene presence or absence in ST131 genomes as a three-dimensional distance matrix using package FactoMiner in R. The accessory genes observed at high (≥97%) or low (≤3%) frequency were filtered out owing to their weak discriminating power and the effect of low-frequency categories (39). The distance matrix was then used in R for clustering the genomes by partitioning around medoids to assign clusters and subclusters with the cluster package. Their number was determined with the same package on the basis of the Gap algorithm using Tibs2001SEmax metric (goodness of clustering measure) (40). The clusters were visually checked in the MCA plot using 95% confidence ellipses. Four outliers were excluded.

### Statistical approaches.

The indexes and statistical tests were also calculated in R with packages ade4, jaccard, corrplot, and vegan. Heritability was calculated as previously reported (20). The indicator values (IndVals) that were used to assess the association of a target feature to a target group were calculated in R from the following equation (41):

$$\mathrm{IndVal}=\;\sqrt{\frac{N_{g}}{N_{t}}\times \frac{N_{g}}{E_{g}}}=\sqrt{A\;\times B},$$

with *N_g_* being the number of occurrences of the target feature within the target group; *N_t_* being the number of occurrences of the target feature in all groups (e.g., all subclusters); *E_g_* being the number of members within the target groups (i.e., number of strains within the target subcluster); *A* being the probability that the target feature (e.g., a virulence gene) belongs to the target group (e.g., a subcluster), namely, the specificity; and *B* being the probability of finding the target feature in the target group, namely, the sensitivity (or fidelity). Permutation test (500 permutations) was used to identify the significant associations (α < 0.05), and the *P* values were adjusted for multiple testing by the FDR procedure.

## References

[1] Banerjee R, Johnson JR . 2014. A new clone sweeps clean: the enigmatic emergence of *Escherichia coli* sequence type 131. Antimicrob Agents Chemother 58:4997–5004. doi:10.1128/AAC.02824-14. 24867985 PMC4135879

[2] Nicolas-Chanoine M-H, Bertrand X, Madec J-Y . 2014. *Escherichia coli* ST131, an intriguing clonal group. Clin Microbiol Rev 27:543–574. doi:10.1128/CMR.00125-13. 24982321 PMC4135899

[3] Pitout JDD, Finn TJ . 2020. The evolutionary puzzle of *Escherichia coli* ST131. Infect Genet Evol 81:104265. doi:10.1016/j.meegid.2020.104265. 32112974

[4] Price LB, Johnson JR, Aziz M, Clabots C, Johnston B, Tchesnokova V, Nordstrom L, Billig M, Chattopadhyay S, Stegger M, Andersen PS, Pearson T, Riddell K, Rogers P, Scholes D, Kahl B, Keim P, Sokurenko EV . 2013. The epidemic of extended-spectrum-β-lactamase-producing *Escherichia coli* ST131 is driven by a single highly pathogenic subclone, H30-Rx. mBio 4:e00377-13. doi:10.1128/mBio.00377-13. 24345742 PMC3870262

[5] Petty NK, Ben Zakour NL, Stanton-Cook M, Skippington E, Totsika M, Forde BM, Phan M-D, Gomes Moriel D, Peters KM, Davies M, Rogers BA, Dougan G, Rodriguez-Bano J, Pascual A, Pitout JDD, Upton M, Paterson DL, Walsh TR, Schembri MA, Beatson SA . 2014. Global dissemination of a multidrug-resistant *Escherichia coli* clone. Proc Natl Acad Sci USA 111:5694–5699. doi:10.1073/pnas.1322678111. 24706808 PMC3992628

[6] Ben Zakour NL, Alsheikh-Hussain AS, Ashcroft MM, Khanh Nhu NT, Roberts LW, Stanton-Cook M, Schembri MA, Beatson SA . 2016. Sequential acquisition of virulence and fluoroquinolone resistance has shaped the evolution of *Escherichia coli* ST131. mBio 7:e00347-16. doi:10.1128/mBio.00347-16. 27118589 PMC4850260

[7] Matsumura Y, Pitout JDD, Peirano G, DeVinney R, Noguchi T, Yamamoto M, Gomi R, Matsuda T, Nakano S, Nagao M, Tanaka M, Ichiyama S . 2017. Rapid identification of different *Escherichia coli* sequence type 131 clades. Antimicrob Agents Chemother 61:e00179-17. doi:10.1128/AAC.00179-17. 28584160 PMC5527616

[8] Stoesser N, Sheppard AE, Pankhurst L, De Maio N, Moore CE, Sebra R, Turner P, Anson LW, Kasarskis A, Batty EM, Kos V, Wilson DJ, Phetsouvanh R, Wyllie D, Sokurenko E, Manges AR, Johnson TJ, Price LB, Peto TEA, Johnson JR, Didelot X, Walker AS, Crook DW, Modernizing Medical Microbiology Informatics Group (MMMIG). 2016. Evolutionary history of the global emergence of the *Escherichia coli* epidemic clone ST131. mBio 7:e02162-15. doi:10.1128/mBio.02162-15. 27006459 PMC4807372

[9] Kallonen T, Brodrick HJ, Harris SR, Corander J, Brown NM, Martin V, Peacock SJ, Parkhill J . 2017. Systematic longitudinal survey of invasive *Escherichia coli* in England demonstrates a stable population structure only transiently disturbed by the emergence of ST131. Genome Res 27:1437–1449. doi:10.1101/gr.216606.116. PMC5538559 28720578

[10] Blanco J, Mora A, Mamani R, López C, Blanco M, Dahbi G, Herrera A, Marzoa J, Fernández V, de la Cruz F, Martínez-Martínez L, Alonso MP, Nicolas-Chanoine M-H, Johnson JR, Johnston B, López-Cerero L, Pascual Á, Rodríguez-Baño J, Spanish Group for Nosocomial Infections (GEIH). 2013. Four main virotypes among extended-spectrum-β-lactamase-producing isolates of *Escherichia coli* O25b:H4-B2-ST131: bacterial, epidemiological, and clinical characteristics. J Clin Microbiol 51:3358–3367. doi:10.1128/JCM.01555-13. 23926164 PMC3811668

[11] McNally A, Oren Y, Kelly D, Pascoe B, Dunn S, Sreecharan T, Vehkala M, Välimäki N, Prentice MB, Ashour A, Avram O, Pupko T, Dobrindt U, Literak I, Guenther S, Schaufler K, Wieler LH, Zhiyong Z, Sheppard SK, McInerney JO, Corander J . 2016. Combined analysis of variation in core, accessory and regulatory genome regions provides a super-resolution view into the evolution of bacterial populations. PLoS Genet 12:e1006280. doi:10.1371/journal.pgen.1006280. 27618184 PMC5019451

[12] McNally A, Kallonen T, Connor C, Abudahab K, Aanensen DM, Horner C, Peacock SJ, Parkhill J, Croucher NJ, Corander J . 2019. Diversification of colonization factors in a multidrug-resistant *Escherichia coli* lineage evolving under negative frequency-dependent selection. mBio 10:e00644-19. doi:10.1128/mBio.00644-19. 31015329 PMC6479005

[13] Duprilot M, Baron A, Blanquart F, Dion S, Pouget C, Lettéron P, Flament-Simon S-C, Clermont O, Denamur E, Nicolas-Chanoine M-H . 2020. Success of *Escherichia coli* O25b:H4 sequence type 131 clade C associated with a decrease in virulence. Infect Immun 88:e00644-19. doi:10.1128/IAI.00576-20. PMC7671891 32989036

[14] Nicolas-Chanoine M-H, Blanco J, Leflon-Guibout V, Demarty R, Alonso MP, Canica MM, Park Y-J, Lavigne J-P, Pitout J, Johnson JR . 2008. Intercontinental emergence of *Escherichia coli* clone O25:H4-ST131 producing CTX-M-15. J Antimicrob Chemother 61:273–281. doi:10.1093/jac/dkm464. 18077311

[15] Müller A, Stephan R, Nüesch-Inderbinen M . 2016. Distribution of virulence factors in ESBL-producing *Escherichia coli* isolated from the environment, livestock, food, and humans. Sci Total Environ 541:667–672. doi:10.1016/j.scitotenv.2015.09.135. 26437344

[16] Liu CM, Stegger M, Aziz M, Johnson TJ, Waits K, Nordstrom L, Gauld L, Weaver B, Rolland D, Statham S, Horwinski J, Sariya S, Davis GS, Sokurenko E, Keim P, Johnson JR, Price LB . 2018. *Escherichia coli* ST131-H22 as a foodborne uropathogen. mBio 9:e00470-18. doi:10.1128/mBio.00470-18. 30154256 PMC6113624

[17] Saidenberg ABS, Stegger M, Price LB, Johannesen TB, Aziz M, Cunha MPV, Moreno AM, Knöbl T . 2020. *mcr*-positive *Escherichia coli* ST131-H22 from poultry in Brazil. Emerg Infect Dis 26:1951–1954. doi:10.3201/eid2608.191724. 32687033 PMC7392447

[18] Melo LC, Haenni M, Saras E, Duprilot M, Nicolas-Chanoine M-H, Madec J-Y . 2019. Emergence of the C1-M27 cluster in ST131 *Escherichia coli* from companion animals in France. J Antimicrob Chemother 74:3111–3113. doi:10.1093/jac/dkz304. 31299071

[19] Decano AG, Ludden C, Feltwell T, Judge K, Parkhill J, Downing T . 2019. Complete assembly of *Escherichia coli* sequence type 131 genomes using long reads demonstrates antibiotic resistance gene variation within diverse plasmid and chromosomal contexts. mSphere 4:e00130-19. doi:10.1128/mSphere.00130-19. 31068432 PMC6506616

[20] Lees JA, Mai TT, Galardini M, Wheeler NE, Horsfield ST, Parkhill J, Corander J . 2020. Improved prediction of bacterial genotype-phenotype associations using interpretable pangenome-spanning regressions. mBio 11:e01344-20. doi:10.1128/mBio.01344-20. 32636251 PMC7343994

[21] Desvaux M, Dalmasso G, Beyrouthy R, Barnich N, Delmas J, Bonnet R . 2020. Pathogenicity factors of genomic islands in intestinal and extraintestinal *Escherichia coli*. Front Microbiol 11:2065. doi:10.3389/fmicb.2020.02065. 33101219 PMC7545054

[22] Delmas J, Gibold L, Faïs T, Batista S, Leremboure M, Sinel C, Vazeille E, Cattoir V, Buisson A, Barnich N, Dalmasso G, Bonnet R . 2019. Metabolic adaptation of adherent-invasive *Escherichia coli* to exposure to bile salts. Sci Rep 9:2175. doi:10.1038/s41598-019-38628-1. 30778122 PMC6379400

[23] Kim S, Covington A, Pamer EG . 2017. The intestinal microbiota: antibiotics, colonization resistance, and enteric pathogens. Immunol Rev 279:90–105. doi:10.1111/imr.12563. 28856737 PMC6026851

[24] Tenaillon O, Skurnik D, Picard B, Denamur E . 2010. The population genetics of commensal *Escherichia coli*. Nat Rev Microbiol 8:207–217. doi:10.1038/nrmicro2298. 20157339

[25] van den Bunt G, Fluit AC, Bootsma MCJ, van Duijkeren E, Scharringa J, van Pelt W, Bonten MJM . 2020. Dynamics of intestinal carriage of extended-spectrum beta-lactamase-producing *Enterobacteriaceae* in the Dutch general population, 2014–2016. Clin Infect Dis 71:1847–1855. doi:10.1093/cid/ciz1091. 31688916

[26] Huijbers PMC, Graat E.aM, Haenen APJ, van Santen MG, van Essen-Zandbergen A, Mevius DJ, van Duijkeren E, van Hoek A.aM . 2014. Extended-spectrum and AmpC β-lactamase-producing *Escherichia coli* in broilers and people living and/or working on broiler farms: prevalence, risk factors and molecular characteristics. J Antimicrob Chemother 69:2669–2675. doi:10.1093/jac/dku178. 24879667

[27] Matsumura Y, Pitout JDD, Gomi R, Matsuda T, Noguchi T, Yamamoto M, Peirano G, DeVinney R, Bradford PA, Motyl MR, Tanaka M, Nagao M, Takakura S, Ichiyama S . 2016. Global *Escherichia coli* sequence type 131 clade with *bla*_CTX-M-27_ gene. Emerg Infect Dis 22:1900–1907. doi:10.3201/eid2211.160519. 27767006 PMC5088012

[28] Totsika M, Beatson SA, Sarkar S, Phan M-D, Petty NK, Bachmann N, Szubert M, Sidjabat HE, Paterson DL, Upton M, Schembri MA . 2011. Insights into a multidrug-resistant *Escherichia coli* pathogen of the globally disseminated ST131 lineage: genome analysis and virulence mechanisms. PLoS One 6:e26578. doi:10.1371/journal.pone.0026578. 22053197 PMC3203889

[29] Kim D, Song L, Breitwieser FP, Salzberg SL . 2016. Centrifuge: rapid and sensitive classification of metagenomic sequences. Genome Res 26:1721–1729. doi:10.1101/gr.210641.116. 27852649 PMC5131823

[30] Wick RR, Judd LM, Gorrie CL, Holt KE . 2017. Unicycler: resolving bacterial genome assemblies from short and long sequencing reads. PLoS Comput Biol 13:e1005595. doi:10.1371/journal.pcbi.1005595. 28594827 PMC5481147

[31] Seemann T . 2014. Prokka: rapid prokaryotic genome annotation. Bioinformatics 30:2068–2069. doi:10.1093/bioinformatics/btu153. 24642063

[32] Araujo FA, Barh D, Silva A, Guimarães L, Ramos RTJ . 2018. GO FEAT: a rapid web-based functional annotation tool for genomic and transcriptomic data. Sci Rep 8:1794. doi:10.1038/s41598-018-20211-9. 29379090 PMC5789007

[33] Hunt M, Mather AE, Sánchez-Busó L, Page AJ, Parkhill J, Keane JA, Harris SR . 2017. ARIBA: rapid antimicrobial resistance genotyping directly from sequencing reads. Microb Genom 3:e000131. doi:10.1099/mgen.0.000131. 29177089 PMC5695208

[34] Buchfink B, Xie C, Huson DH . 2015. Fast and sensitive protein alignment using DIAMOND. Nat Methods 12:59–60. doi:10.1038/nmeth.3176. 25402007

[35] Yoshimura D, Kajitani R, Gotoh Y, Katahira K, Okuno M, Ogura Y, Hayashi T, Itoh T . 2019. Evaluation of SNP calling methods for closely related bacterial isolates and a novel high-accuracy pipeline: BactSNP. Microb Genom 5:e000261. 10.1099/mgen.0.000261 PMC6562250 31099741

[36] Kozlov AM, Darriba D, Flouri T, Morel B, Stamatakis A . 2019. RAxML-NG: a fast, scalable and user-friendly tool for maximum likelihood phylogenetic inference. Bioinformatics 35:4453–4455. doi:10.1093/bioinformatics/btz305. 31070718 PMC6821337

[37] Croucher NJ, Page AJ, Connor TR, Delaney AJ, Keane JA, Bentley SD, Parkhill J, Harris SR . 2015. Rapid phylogenetic analysis of large samples of recombinant bacterial whole-genome sequences using Gubbins. Nucleic Acids Res 43:e15. doi:10.1093/nar/gku1196. 25414349 PMC4330336

[38] Gautreau G, Bazin A, Gachet M, Planel R, Burlot L, Dubois M, Perrin A, Médigue C, Calteau A, Cruveiller S, Matias C, Ambroise C, Rocha EPC, Vallenet D . 2020. PPanGGOLiN: depicting microbial diversity via a partitioned pangenome graph. PLoS Comput Biol 16:e1007732. doi:10.1371/journal.pcbi.1007732. 32191703 PMC7108747

[39] Di Franco G . 2016. Multiple correspondence analysis: one only or several techniques? Qual Quant 50:1299–1315. doi:10.1007/s11135-015-0206-0.

[40] Tibshirani R, Walther G, Hastie T . 2001. Estimating the number of clusters in a data set via the gap statistic. J R Stat Soc Ser B Stat Methodol 63:411–423. doi:10.1111/1467-9868.00293.

[41] Cáceres MD, Legendre P . 2009. Associations between species and groups of sites: indices and statistical inference. Ecology 90:3566–3574. doi:10.1890/08-1823.1. 20120823

