# Supplementary figures and images for: Host Colonization as a Major Evolutionary Force Favoring the Diversity and the Emergence of the Worldwide Multidrug-Resistant Escherichia coli ST131
Source: mBio. 2021 Aug 24;12(4):e01451-21. doi: 10.1128/mBio.01451-21 (PMC8406181; doi:10.1128/mBio.01451-21)

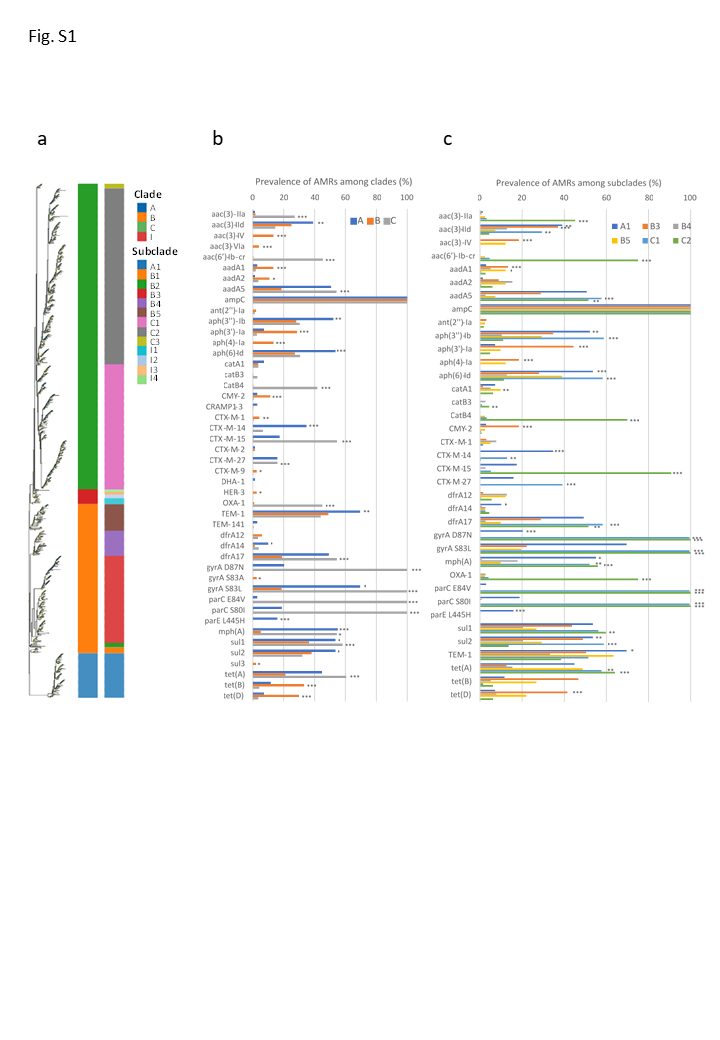

Supplement: FIG S1 [file mbio.01451-21-sf001.tif]

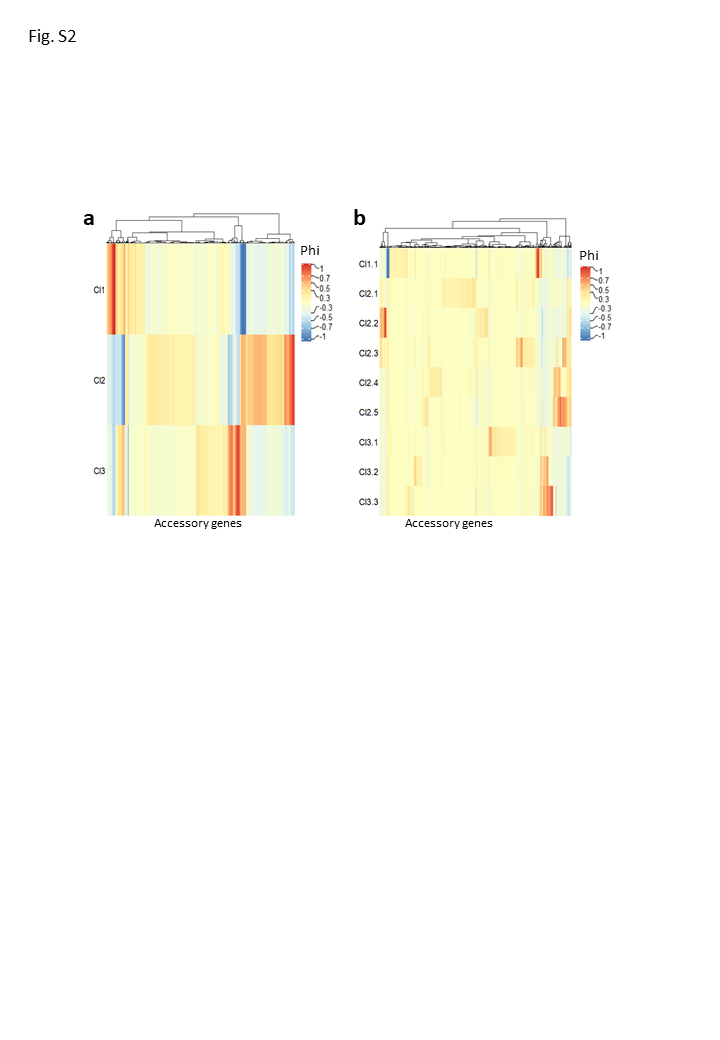

Supplement: FIG S2 [file mbio.01451-21-sf002.tif]

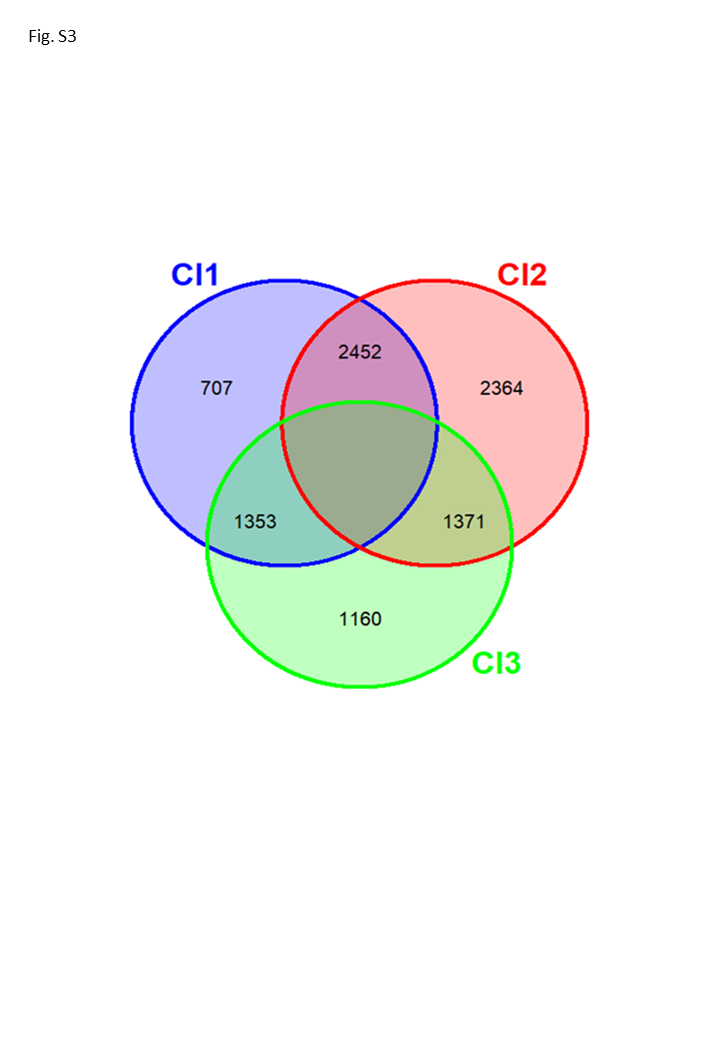

Supplement: FIG S3 [file mbio.01451-21-sf003.tif]

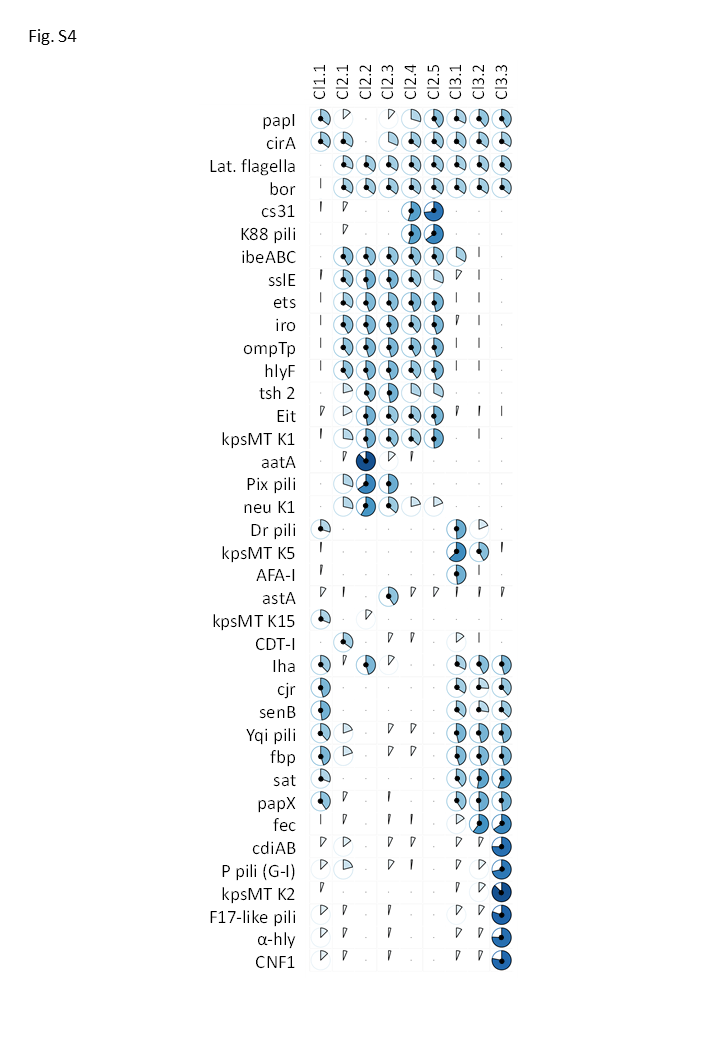

Supplement: FIG S4 [file mbio.01451-21-sf004.tif]

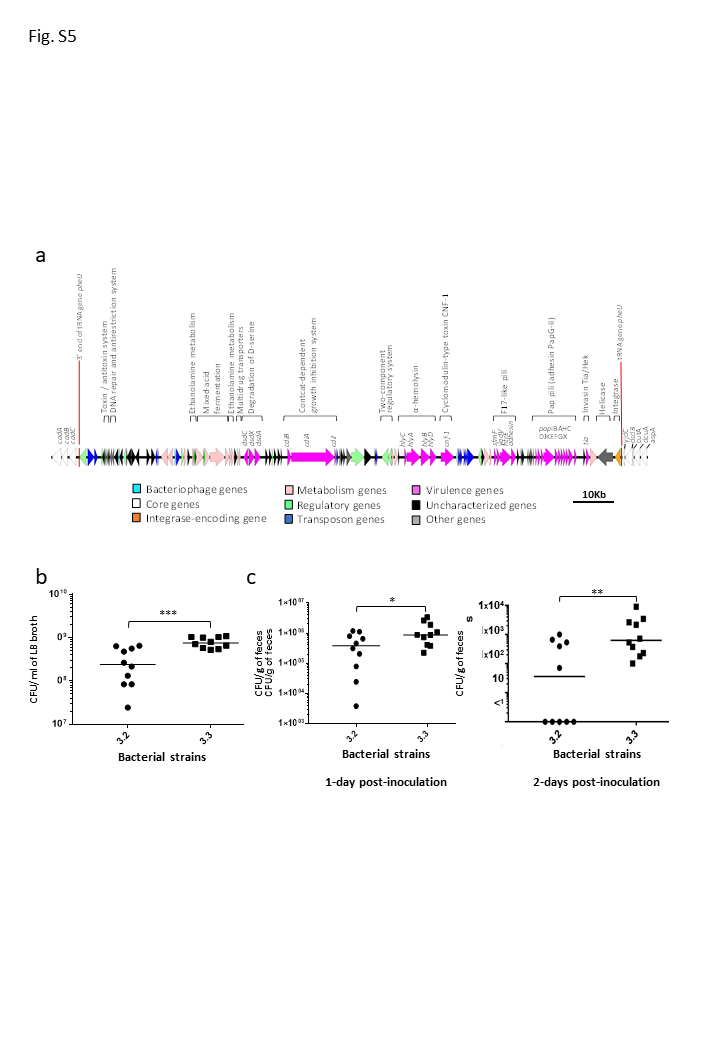

Supplement: FIG S5 [file mbio.01451-21-sf005.tif]

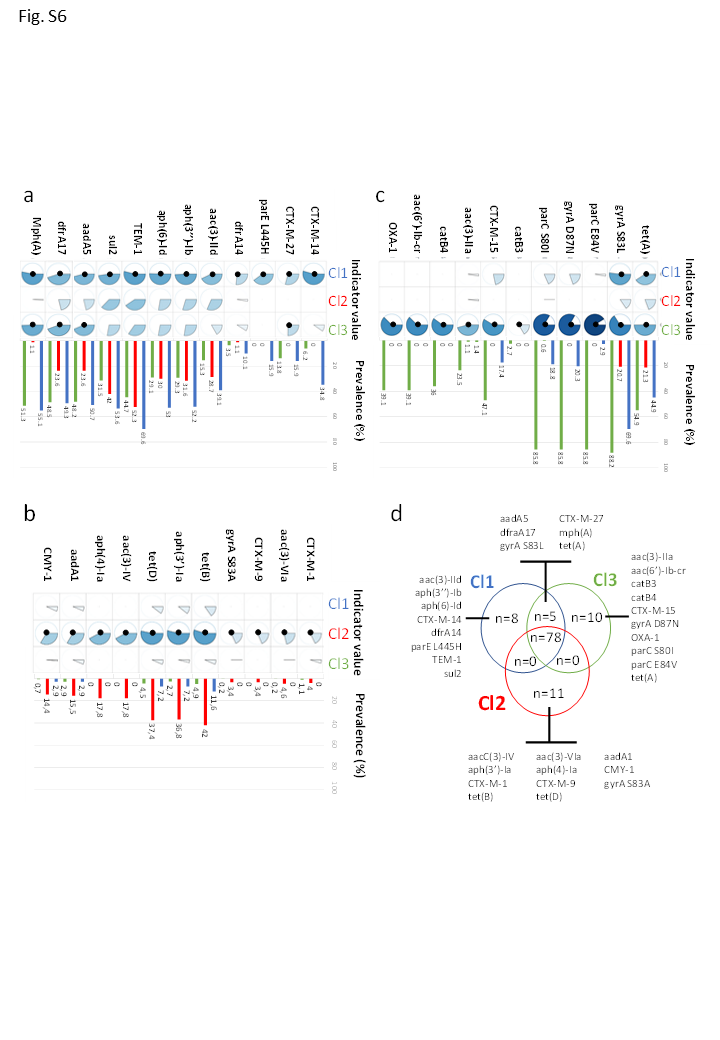

Supplement: FIG S6 [file mbio.01451-21-sf006.tif]

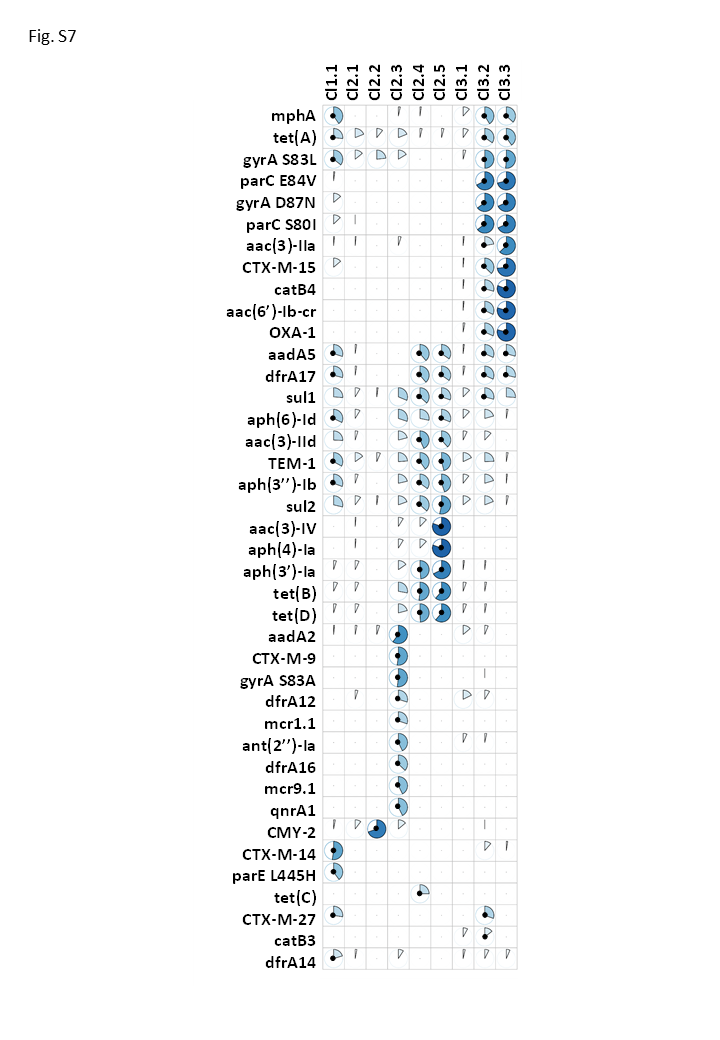

Supplement: FIG S7 [file mbio.01451-21-sf007.tif]

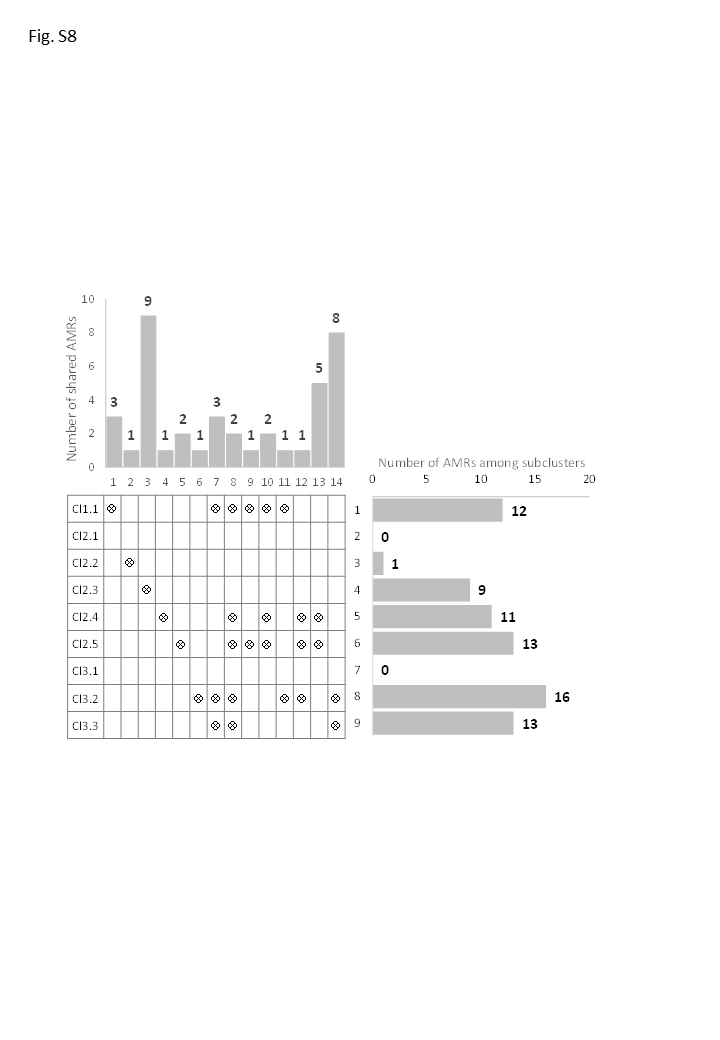

Supplement: FIG S8 [file mbio.01451-21-sf008.tif]
