# Supplementary material for: Host Colonization as a Major Evolutionary Force Favoring the Diversity and the Emergence of the Worldwide Multidrug-Resistant Escherichia coli ST131
Source: mBio. 2021 Aug 24;12(4):e01451-21. doi: 10.1128/mBio.01451-21 (PMC8406181; doi:10.1128/mBio.01451-21)
Supplement: TABLE S1 [file mbio.01451-21-st001.pdf]

Supplementary Table 1: The genomes included in the study, associated metadata, assigned phylogenomic clades/subsclades, pangenomic clusters/subclusters and genetic features. Avian sources, which correspond to food-producing animals (i.e. poultry) were separated from wild birds.

Strain	Accession	Genome size	Contig number	N50	Gene number	Country	Year	Host[a]	Clade	Subclade	Cluster	Subcluster	Serotype	FimH	CTX-M ESBL	GyrA	ParC
1	ERS1382645	5096797	128	150389	4797	Sweden	2016	Human	C	C2	Cl3	Cl3.2	O25b:H4	fimH35	CTX-M-15	gyrA L83, N87	parC I80, V84
2	ERS1382646	5102801	80	277261	4802	Sweden	2016	Human	C	C2	Cl3	Cl3.2	O25b:H4	fimH35	CTX-M-15	gyrA L83, N87	parC I80, V84
3	ERS1382647	5144209	109	276245	4853	Sweden	2016	Human	C	C2	Cl3	Cl3.2	O25b:H4	fimH35	CTX-M-15	gyrA L83, N87	parC I80, V84
4	ERS1382648	5150797	107	223074	4872	Sweden	2016	Human	C	C2	Cl3	Cl3.2	O25b:H4	fimH35	CTX-M-15	gyrA L83, N87	parC I80, V84
5	ERS1382649	5140054	115	190458	4850	Sweden	2016	Human	C	C2	Cl3	Cl3.2	O25b:H4	fimH35	CTX-M-15	gyrA L83, N87	parC I80, V84
6	ERS1382725	5152054	123	164688	4864	Sweden	2016	Human	C	C2	Cl3	Cl3.2	O25b:H4	fimH35	CTX-M-15	gyrA L83, N87	parC I80, V84
7	ERS1382726	5115362	123	167300	4807	Sweden	2016	Human	C	C2	Cl3	Cl3.2	O25b:H4	fimH35	CTX-M-15	gyrA L83, N87	parC I80, V84
8	ERS1382727	5046615	112	163508	4724	Sweden	2016	Human	C	C1	Cl3	Cl3.2	O25b:H4	fimH30	CTX-M-15	gyrA L83, N87	parC I80, V84
12	ERS1382731	5153050	134	177914	4858	Sweden	2016	Human	C	C2	Cl3	Cl3.2	O25b:H4	fimH35	CTX-M-15	gyrA L83, N87	parC I80, V84
13	ERS1382732	5128433	139	143973	4808	Sweden	2016	Human	C	C2	Cl3	Cl3.2	O25b:H4	fimH30	CTX-M-15	gyrA L83, N87	parC I80, V84
14	ERS1382733	5164812	150	286051	4863	Sweden	2016	Human	C	C2	Cl3	Cl3.2	O25b:H4	fimH35-like	CTX-M-15	gyrA L83, N87	parC I80, V84
16	ERS1382735	5163959	143	185168	4856	Sweden	2016	Human	C	C2	Cl3	Cl3.2	O25b:H4	fimH35	CTX-M-15	gyrA L83, N87	parC I80, V84
852	SRS1263374	5139657	67	234086	4837	Germany	2011	Human	C	C2	Cl3	Cl3.2	O25b:H4	fimH30		gyrA L83, N87	parC I80, V84
972	SRS1274427	5211766	104	197429	4916	Germany	2011	Human	C	C1	Cl3	Cl3.2	O25b:H4	fimH30		gyrA L83, N87	parC I80, V84
1011	SRS1274444	5085226	57	260239	4737	Germany	2011	Human	B	B5	Cl2	Cl2.1	O25b:H4	fimH22		gyrA S83, N87	parC S80, E84
1019	SRS1274443	5093248	104	162169	4794	Germany	2011	Human	C	C1	Cl3	Cl3.2	O25b:H4	fimH30		gyrA L83, N87	parC I80, V84
1039	SRS1274442	5097522	80	251551	4791	Germany	2011	Human	C	C2	Cl3	Cl3.2	O25b:H4	fimH30		gyrA L83, N87	parC I80, V84
1087	SRS1274441	5068299	103	161155	4754	Germany	2011	Human	I	I4	Cl3	Cl3.2	O25b:H4	fimH30-like		gyrA S83, N87	parC S80, E84
1223	SRS1274440	5068544	103	173835	4756	Germany	2011	Human	C	C1	Cl3	Cl3.2	O25b:H4	fimH30		gyrA L83, N87	parC I80, V84
1351	SRS1274439	5018731	54	284392	4690	Germany	2011	Human	B	B4	Cl3	Cl3.1	O25b:H4	fimH22		gyrA S83, N87	parC S80, E84
1366	SRS1274438	5125842	80	244732	4812	Germany	2011	Human	B	B4	Cl3	Cl3.1	O25b:H4	fimH22		gyrA S83, N87	parC S80, E84
1369	SRS1274437	5153775	92	191026	4878	Germany	2011	Human	C	C2	Cl3	Cl3.2	O25b:H4	fimH30		gyrA L83, N87	parC I80, V84
1389	SRS1274435	5096641	76	229816	4788	Germany	2011	Human	C	C2	Cl3	Cl3.2	O25b:H4	fimH30		gyrA L83, N87	parC I80, V84
1402	SRS1274434	5125916	106	181260	4837	Germany	2011	Human	C	C2	Cl3	Cl3.2	O25b:H4	fimH30		gyrA L83, N87	parC I80, V84
2963	SRS1274433	5108582	120	157541	4781	Germany	2011	Human	C	C1	Cl3	Cl3.2	O25b:H4	fimH30	CTX-M-27	gyrA L83, N87	parC I80, V84
2999	SRS1274432	5114129	95	216488	4774	Germany	2011	Human	B	B1	Cl2	Cl2.1	O25b:H4	fimH22		gyrA S83, N87	parC S80, E84
3019	SRS1274431	5188664	63	235887	4884	Germany	2011	Human	B	B5	Cl2	Cl2.1	O25b:H4	fimH22		gyrA S83, N87	parC S80, E84
3020	SRS1274430	5066731	90	173833	4749	Germany	2011	Human	C	C1	Cl3	Cl3.2	O25b:H4	fimH30		gyrA L83, N87	parC I80, V84
3134	SRS1274429	5063990	80	195054	4752	Germany	2011	Human	C	C2	Cl3	Cl3.2		fimH30		gyrA L83, N87	parC I80, V84
3140	SRS1274428	5194753	128	173820	4902	Germany	2011	Human	C	C1	Cl3	Cl3.2	O25b:H4	fimH30		gyrA L83, N87	parC I80, V84
81009	SRR5482170	5283524	90	222552	4977	UAE	2009	Human	C	C1	Cl3	Cl3.2	O25b:H4	fimH30	CTX-M-27	gyrA L83, N87	parC I80, V84
001-001	SRS5403698	5132872	195	175888	4768	France	2010	Human	B	B4	Cl3	Cl3.1	O25b:H4	fimH22-like		gyrA S83, N87	parC S80, E84
08B08158	SRR2970754	5619730	117	122552	5374	Thailand	2008	Human	I	I1			O25b:H4	fimH5		gyrA S83, N87	parC S80, E84
08B09891	SRR2970755	5199325	80	173688	4900	Thailand	2008	Human	C	C2	Cl3	Cl3.2	O25b:H4	fimH30	CTX-M-15	gyrA L83, N87	parC I80, V84
09B06064	SRR2970756	5199988	79	173688	4898	Thailand	2009	Human	C	C2	Cl3	Cl3.2	O25b:H4	fimH30	CTX-M-15	gyrA L83, N87	parC I80, V84
09B06312	SRR2970757	5093074	129	173799	4755	Thailand	2009	Human	C	C1	Cl3	Cl3.2	O25b:H4	fimH30	CTX-M-27	gyrA L83, N87	parC I80, V84
09B06460	SRR2970758	5232007	76	186355	4928	Thailand	2009	Human	C	C2	Cl3	Cl3.2	O25b:H4	fimH30	CTX-M-15	gyrA L83, N87	parC I80, V84
09B06574	SRR2970759	5350366	85	186355	5028	Thailand	2009	Human	C	C2	Cl3	Cl3.2	O25b:H4	fimH30	CTX-M-15	gyrA L83, N87	parC I80, V84
09B06576	SRR2970760	5203338	72	186345	4910	Thailand	2009	Human	C	C2	Cl3	Cl3.2	O25b:H4	fimH30	CTX-M-15	gyrA L83, N87	parC I80, V84
09B07697	SRR2970761	5299633	136	124114	5017	Thailand	2009	Human	I	I1	Cl3	Cl3.2	O25b:H4	fimH27		gyrA S83, N87	parC S80, E84
09B09003	SRR2970763	5224577	99	136475	4919	Thailand	2009	Human	C	C2	Cl3	Cl3.2	O25b:H4	fimH30	CTX-M-15	gyrA L83, N87	parC I80, V84
09B09491	SRR2970764	5201381	78	175114	4901	Thailand	2009	Human	C	C2	Cl3	Cl3.2	O25b:H4	fimH30	CTX-M-15	gyrA L83, N87	parC I80, V84
09B11376	SRR2970765	5327866	91	172926	5049	Thailand	2009	Human	C	C2	Cl3	Cl3.2	O25b:H4	fimH30	CTX-M-15	gyrA L83, N87	parC I80, V84
09B13160	SRR2970766	5206135	79	186358	4913	Thailand	2009	Human	C	C2	Cl3	Cl3.2	O25b:H4	fimH30	CTX-M-15	gyrA L83, N87	parC I80, V84
09B13464	SRR2970767	5081049	100	124114	4770	Thailand	2009	Human	I	I1	Cl3	Cl3.2	O25b:H4	fimH27		gyrA L83, N87	parC S80, E84
10B05056	SRR2970768	5311582	154	159291	5038	Thailand	2010	Human	A	A1	Cl1	Cl1.1	O16:H5	fimH41		gyrA L83, N87	parC S80, E84
10B05087	SRR2970769	5328268	66	209569	5058	Thailand	2010	Human	C	C2	Cl3	Cl3.3	O25b:H4	fimH30	CTX-M-15	gyrA L83, N87	parC I80, V84
10B05611	SRR2970770	5198189	96	191001	4888	Thailand	2010	Human	C	C2	Cl3	Cl3.2	O25b:H4	fimH30	CTX-M-15	gyrA L83, N87	parC I80, V84
10B05736	SRR2970771	5460667	89	188213	5176	Thailand	2010	Human	C	C2	Cl3	Cl3.3	O25b:H4	fimH30	CTX-M-15	gyrA L83, N87	parC I80, V84

10B06164	SRR2970733	5293281	73	170358	5013	Thailand	2010	Human	C	C2	Cl3	Cl3.3	O25b:H4	fimH30		gyrA L83, N87	parC I80, V84
10B06797	SRR2970734	5279980	74	172177	5002	Thailand	2010	Human	C	C2	Cl3	Cl3.3	O25b:H4	fimH30		gyrA L83, N87	parC I80, V84
10B07347	SRR2970748	5280023	134	140954	4980	Thailand	2010	Human	C	C2	Cl3	Cl3.2	O25b:H4	fimH30	CTX-M-15	gyrA L83, N87	parC I80, V84
10B07395	SRR2970735	5209832	108	142077	4903	Thailand	2010	Human	I	I1	Cl3	Cl3.2	O25b:H4	fimH27		gyrA L83, N87	parC S80, E84
11B00062	SRR2970738	5452634	108	158810	5208	Thailand	2011	Human	C	C2	Cl3	Cl3.3	O25b:H4	fimH30	CTX-M-15	gyrA L83, N87	parC I80, V84
11B00094	SRR2970739	5310812	86	185032	5039	Thailand	2011	Human	C	C2	Cl3	Cl3.3	O25b:H4	fimH30		gyrA L83, N87	parC I80, V84
11B00134	SRR2970741	5086122	99	124713	4752	Thailand	2011	Human	C	C1	Cl3	Cl3.2	O25b:H4	fimH30	CTX-M-27	gyrA L83, N87	parC I80, V84
11B00320	SRR2970742	5020744	118	141696	4665	Thailand	2011	Human	A	A1	Cl1	Cl1.1	O16:H5	fimH41	CTX-M-15	gyrA L83, N87	parC S80, E84
11B00663	SRR2970743	5216207	89	173436	4893	Thailand	2011	Human	I	I1	Cl3	Cl3.2	O25b:H4	fimH27		gyrA S83, N87	parC S80, E84
11B00726	SRR2970744	5245324	93	172437	4946	Thailand	2011	Human	I	I1	Cl3	Cl3.2	O25b:H4	fimH5		gyrA S83, N87	parC S80, E84
11B00806	SRR2970745	4973613	102	157430	4644	Thailand	2011	Human	A	A1	Cl1	Cl1.1	O16:H5	fimH41		gyrA L83, N87	parC S80, E84
11B01631	SRR2970746	5187585	94	149220	4895	Thailand	2011	Human	I	I1	Cl3	Cl3.2	O25b:H4	fimH27		gyrA S83, N87	parC S80, E84
11B01979	SRR2970747	5316647	111	161041	5012	Thailand	2011	Human	C	C2	Cl3	Cl3.2	O25b:H4	fimH30	CTX-M-15	gyrA L83, N87	parC I80, V84
a19470	SAMEA7758127	5141080	132	102221	4787	Croatia	2017	Avian	B	B3	Cl2	Cl2.1	O25b:H4	fimH22		gyrA L83, N87	parC S80, E84
a21190	SAMEA7758128	5083457	348	33176	4723	Croatia	2017	Avian	B	B3	Cl2	Cl2.1		fimH22		gyrA L83, N87	parC S80, E84
a25795	SAMEA7758129	5090651	80	212627	4798	France	2010	Canine	C	C1	Cl3	Cl3.2	O25b:H4	fimH30	CTX-M-14	gyrA L83, N87	parC I80, V84
a25822	SAMEA7758130	5400732	101	167701	5150	France	2010	Canine	C	C2	Cl3	Cl3.3	O25b:H4	fimH30	CTX-M-15	gyrA L83, N87	parC I80, V84
a27046	SAMEA7758131	5314046	89	180948	5062	France	2011	Canine	C	C2	Cl3	Cl3.3	O25b:H4	fimH30	CTX-M-15	gyrA L83, N87	parC I80, V84
a27608	SAMEA7758132	5039673	65	186390	4755	France	2011	Feline	C	C1	Cl3	Cl3.2	O25b:H4	fimH30	CTX-M-14	gyrA L83, N87	parC I80, V84
a27610	SAMEA7758133	5320766	82	208133	5075	France	2011	Canine	C	C2	Cl3	Cl3.3	O25b:H4	fimH30	CTX-M-15	gyrA L83, N87	parC I80, V84
a27611	SAMEA7758134	5080911	84	173730	4749	France	2011	Canine	C	C1	Cl3	Cl3.2	O25b:H4	fimH30	CTX-M-27	gyrA L83, N87	parC I80, V84
a32551	SAMEA7758135	5311207	85	186391	5051	France	2012	Canine	C	C2	Cl3	Cl3.3	O25b:H4	fimH30	CTX-M-15	gyrA L83, N87	parC I80, V84
a32561	SAMEA7758136	5346924	88	307863	5082	France	2012	Canine	B	B5	Cl2	Cl2.1	O25b:H4	fimH22	CTX-M-1	gyrA S83, N87	parC S80, E84
a32573	SAMEA7758137	5143534	75	191152	4826	France	2012	Feline	A	A1	Cl1	Cl1.1	O16:H5	fimH41	CTX-M-14	gyrA L83, N87	parC S80, E84
a32580	SAMEA7758138	5146277	77	224309	4830	France	2012	Feline	A	A1	Cl1	Cl1.1	O16:H5	fimH41	CTX-M-14	gyrA L83, N87	parC S80, E84
a32795	SAMEA7758139	5325616	81	229402	5080	France	2012	Canine	C	C2	Cl3	Cl3.3	O25b:H4	fimH30	CTX-M-15	gyrA L83, N87	parC I80, V84
a32907	SAMEA7758140	5170178	68	229405	4906	France	2012	Canine	C	C2	Cl3	Cl3.3	O25b:H4	fimH30	CTX-M-15	gyrA L83, N87	parC I80, V84
a33680	SAMEA7758141	5050931	90	173499	4724	France	2012	Horse	C	C1	Cl3	Cl3.2	O25b:H4	fimH30	CTX-M-27	gyrA L83, N87	parC I80, V84
a33681	SAMEA7758142	5060880	69	222833	4716	France	2012	Canine	C	C1	Cl3	Cl3.2	O25b:H4	fimH30	CTX-M-27	gyrA L83, N87	parC I80, V84
a33731	SAMEA7758143	5197897	68	186467	4901	France	2013	Canine	C	C2	Cl3	Cl3.2	O25b:H4	fimH30	CTX-M-15	gyrA L83, N87	parC I80, V84
a33732	SAMEA7758144	5150437	72	235715	4853	France	2013	Feline	C	C2	Cl3	Cl3.2	O25b:H4	fimH30	CTX-M-15	gyrA L83, N87	parC I80, V84
a33734	SAMEA7758145	5169652	78	191771	4902	France	2013	Canine	C	C2	Cl3	Cl3.3	O25b:H4	fimH30	CTX-M-15	gyrA L83, N87	parC I80, V84
a33735	SAMEA7758146	5096576	64	209703	4778	France	2013	Canine	C	C2	Cl3	Cl3.2	O25b:H4	fimH30	CTX-M-15	gyrA L83, N87	parC I80, V84
a34114	SAMEA7758147	5211886	70	186469	4948	France	2013	Canine	C	C2	Cl3	Cl3.3	O25b:H4	fimH30	CTX-M-15	gyrA L83, N87	parC I80, V84
a34117	SAMEA7758148	5169894	69	173729	4898	France	2013	Canine	C	C2	Cl3	Cl3.3	O25b:H4	fimH30	CTX-M-15	gyrA L83, N87	parC I80, V84
a34145	SAMEA7758149	5023199	75	186386	4689	France	2013	Canine	C	C1	Cl3	Cl3.2	O25b:H4	fimH30	CTX-M-27	gyrA L83, N87	parC I80, V84
a34180	SAMEA7758150	5047140	61	177100	4774	France	2013	Canine	C	C1	Cl3	Cl3.2	O25b:H4	fimH30	CTX-M-14	gyrA L83, N87	parC I80, V84
a34961	SAMEA7758151	5056334	92	116912	4726	France	2013	Canine	C	C1	Cl3	Cl3.2	O25b:H4	fimH30	CTX-M-27	gyrA L83, N87	parC I80, V84
a34978	SAMEA7758152	5085193	83	173727	4766	France	2013	Canine	C	C2	Cl3	Cl3.2	O25b:H4	fimH30	CTX-M-15	gyrA L83, N87	parC I80, V84
a36001	SAMEA7758153	5319842	96	191771	5066	France	2014	Canine	C	C2	Cl3	Cl3.3	O25b:H4	fimH30	CTX-M-15	gyrA L83, N87	parC I80, V84
a36228	SAMEA7758154	5512264	124	173729	5308	France	2014	Feline	C	C2	Cl3	Cl3.3	O25b:H4	fimH30	CTX-M-15	gyrA L83, N87	parC I80, V84
a37686	SAMEA7758155	5288769	97	186389	5030	France	2014	Canine	C	C2	Cl3	Cl3.3	O25b:H4	fimH30	CTX-M-15	gyrA L83, N87	parC I80, V84
a37695	SAMEA7758156	5061352	80	186389	4749	France	2014	Canine	C	C1	Cl3	Cl3.2	O25b:H4	fimH30	CTX-M-27	gyrA L83, N87	parC I80, V84
a38213	SAMEA7758157	5361347	99	173728	5121	France	2014	Canine	C	C2	Cl3	Cl3.3	O25b:H4	fimH30	CTX-M-15	gyrA L83, N87	parC I80, V84
a38632	SAMEA7758158	5133220	97	182857	4826	France	2014	Canine	C	C1	Cl3	Cl3.2	O25b:H4	fimH30	CTX-M-27	gyrA L83, N87	parC I80, V84
a39572	SAMEA7758159	5021807	76	166916	4741	France	2015	Canine	C	C1	Cl3	Cl3.2	O25b:H4	fimH30	CTX-M-14	gyrA L83, N87	parC I80, V84
a39584	SAMEA7758160	5347067	83	211687	5076	France	2014	Canine	C	C2	Cl3	Cl3.3	O25b:H4	fimH30	CTX-M-15	gyrA L83, N87	parC I80, V84
a39778	SAMEA7758161	5302974	94	158913	5055	France	2014	Canine	C	C2	Cl3	Cl3.3	O25b:H4	fimH30	CTX-M-15	gyrA L83, N87	parC I80, V84
a39784	SAMEA7758162	5136936	77	173732	4842	France	2015	Canine	C	C1	Cl3	Cl3.2	O25b:H4	fimH30		gyrA L83, N87	parC I80, V84
a40309	SAMEA7758163	5309911	68	229405	5026	France	2015	Canine	C	C2	Cl3	Cl3.3	O25b:H4	fimH30	CTX-M-15	gyrA L83, N87	parC I80, V84
a40311	SAMEA7758164	5274186	93	186390	5005	France	2015	Canine	C	C2	Cl3	Cl3.3	O25b:H4	fimH30	CTX-M-15	gyrA L83, N87	parC I80, V84
a42008	SAMEA7758165	5119556	102	153770	4811	France	2015	Canine	C	C1	Cl3	Cl3.2	O25b:H4	fimH30	CTX-M-27	gyrA L83, N87	parC I80, V84
a42017	SAMEA7758166	5188736	65	180832	4899	France	2015	Canine	B	B4	Cl3	Cl3.1	O25b:H4	fimH30	CTX-M-1	gyrA S83, N87	parC S80, E84
a42020	SAMEA7758167	5192991	65	192539	4904	France	2015	Feline	B	B4	Cl3	Cl3.1	O25b:H4	fimH30	CTX-M-1	gyrA S83, N87	parC S80, E84
a42149	SAMEA7758168	5144708	90	186466	4844	France	2015	Canine	C	C2	Cl3	Cl3.2	O25b:H4	fimH30	CTX-M-15	gyrA L83, N87	parC I80, V84
a42151	SAMEA7758169	5222469	95	222123	4945	France	2015	Canine	C	C2	Cl3	Cl3.3	O25b:H4	fimH30	CTX-M-15	gyrA L83, N87	parC I80, V84

a42490	SAMEA7758170	5268460	80	140860	4970	France	2015	Feline	C	C2	Cl3	Cl3.2	O25b:H4	fimH30	CTX-M-15	gyrA L83, N87	parC I80, V84
a42506	SAMEA7758171	5190518	68	204371	4900	France	2015	Feline	B	B4	Cl3	Cl3.1	O25b:H4	fimH30	CTX-M-1	gyrA S83, N87	parC S80, E84
a42897	SAMEA7758172	5224006	87	158854	4913	France	2015	Feline	C	C2	Cl3	Cl3.2	O25b:H4	fimH30	CTX-M-15	gyrA L83, N87	parC I80, V84
a42900	SAMEA7758173	5133950	84	176510	4830	France	2015	Canine	C	C2	Cl3	Cl3.2	O25b:H4	fimH30	CTX-M-15	gyrA L83, N87	parC I80, V84
a42902	SAMEA7758174	5036609	84	146600	4696	France	2015	Canine	C	C1	Cl3	Cl3.2	O25b:H4	fimH30	CTX-M-27	gyrA L83, N87	parC I80, V84
a43116	SAMEA7758175	5227416	92	170065	4956	France	2015	Canine	C	C2	Cl3	Cl3.3	O25b:H4	fimH30	CTX-M-15	gyrA L83, N87	parC I80, V84
a43117	SAMEA7758176	5131981	79	156150	4869	France	2015	Canine	C	C2	Cl3	Cl3.2	O25b:H4	fimH30	CTX-M-15	gyrA L83, N87	parC I80, V84
a43449	SAMEA7758177	5316273	96	159148	5060	France	2015	Canine	C	C2	Cl3	Cl3.3	O25b:H4	fimH30	CTX-M-15	gyrA L83, N87	parC I80, V84
a43661	SAMEA7758178	5022423	71	191796	4665	France	2016	Feline	A	A1	Cl1	Cl1.1	O16:H5	fimH41	CTX-M-27	gyrA L83, N87	parC S80, E84
a43668	SAMEA7758179	5018721	91	173730	4691	France	2016	Canine	C	C1	Cl3	Cl3.2	O25b:H4	fimH30	CTX-M-27	gyrA L83, N87	parC I80, V84
a43671	SAMEA7758180	5077375	82	165393	4779	France	2016	Canine	C	C1	Cl3	Cl3.2	O25b:H4	fimH30	CTX-M-14	gyrA L83, N87	parC I80, V84
a43682	SAMEA7758181	5225822	94	172686	4963	France	2016	Feline	C	C2	Cl3	Cl3.3	O25b:H4	fimH30	CTX-M-15	gyrA L83, N87	parC I80, V84
a44082	SAMEA7758182	5066456	53	212937	4755	France	2016	Feline	C	C2	Cl3	Cl3.2	O25b:H4	fimH30	CTX-M-15	gyrA L83, N87	parC I80, V84
a44086	SAMEA7758183	5351728	84	180948	5125	France	2016	Canine	C	C2	Cl3	Cl3.3	O25b:H4	fimH30	CTX-M-15	gyrA L83, N87	parC I80, V84
a44547	SAMEA7758184	5229038	116	212554	4939	France	2016	Canine	C	C2	Cl3	Cl3.2	O25b:H4	fimH30	CTX-M-15	gyrA L83, N87	parC I80, V84
a44551	SAMEA7758185	5038449	78	191796	4678	France	2016	Canine	A	A1	Cl1	Cl1.1	O16:H5	fimH41	CTX-M-14	gyrA S83, N87	parC S80, E84
a44553	SAMEA7758186	5075920	70	204880	4728	France	2016	Canine	B	B3	Cl2	Cl2.1		fimH22	CTX-M-1	gyrA S83, N87	parC S80, E84
a44557	SAMEA7758187	5015182	81	205780	4674	France	2016	Canine	C	C1	Cl3	Cl3.2	O25b:H4	fimH30	CTX-M-27	gyrA L83, N87	parC I80, V84
a48760	SAMEA7758188	5332330	100	460900	4961	Denmark	2015	Avian	B	B3	Cl2	Cl2.2	O25b:H4	fimH22		gyrA S83, N87	parC S80, E84
AURA44	SAMEA7758189	5095163	90	169601	4791	France	2019	Human	A	A1	Cl1	Cl1.1	O16:H5	fimH41	CTX-M-15	gyrA L83, N87	parC S80, E84
AURA46	SAMEA7758190	5096702	103	146317	4765	France	2019	Human	C	C2	Cl3	Cl3.2	O25b:H4	fimH30	CTX-M-15	gyrA L83, N87	parC I80, V84
AURA49	SAMEA7758191	4988010	92	169937	4630	France	2019	Human	A	A1	Cl1	Cl1.1	O16:H5	fimH41	CTX-M-15	gyrA L83, N87	parC I80, V84
AVC183	SRS3500511	5124116	114	272832	4807	Australia	2010	Avian	B	B3	Cl2	Cl2.1	O25b:H4	fimH22		gyrA S83, N87	parC S80, E84
AZ600324	SRR2970645	5291923	108	159136	5027	China	2009	Human	C	C2	Cl3	Cl3.2	O25b:H4	fimH30	CTX-M-15	gyrA L83, N87	parC I80, V84
AZ647978	SRR2970647	5043642	71	146418	4761	Venezuela	2010	Human	C	C1	Cl3	Cl3.2	O25b:H4	fimH30		gyrA L83, N87	parC I80, V84
AZ657898	SRR2970644	4986898	73	186460	4654	Germany	2010	Human	C	C1	Cl3	Cl3.2	O25b:H4	fimH30		gyrA L83, N87	parC I80, V84
AZ684313	SRR2970654	5044414	76	186349	4733	France	2010	Human	C	C1	Cl3	Cl3.2	O25b:H4	fimH30		gyrA L83, N87	parC I80, V84
AZ720925	SRR2970649	4968872	71	191167	4650	Taiwan	2011	Human	A	A1	Cl1	Cl1.1	O16:H5	fimH41		gyrA L83, N87	parC I80, E84
AZ726786	SRR2970648	5432791	137	138935	5170	Taiwan	2011	Human	C	C1	Cl3	Cl3.2	O25b:H4	fimH30		gyrA L83, N87	parC I80, V84
AZ727008	SRR2970652	5421114	122	158986	5149	Taiwan	2011	Human	C	C2	Cl3	Cl3.2	O25b:H4	fimH30	CTX-M-15	gyrA L83, N87	parC I80, V84
AZ735521	SRR2970653	5025917	108	177602	4701	Spain	2011	Human	A	A1	Cl1	Cl1.1	O16:H5	fimH41		gyrA L83, N87	parC S80, E84
AZ779845	SRR2970651	5128699	73	172289	4847	Spain	2011	Human	A	A1	Cl1	Cl1.1	O16:H5	fimH41	CTX-M-15	gyrA S83, N87	parC S80, E84
B104	SRS1274426	5324262	119	173833	5070	UK	2012	Human	C	C2	Cl3	Cl3.3	O25b:H4	fimH30		gyrA L83, N87	parC I80, V84
B11J12	SRS5403661	5079239	180	180448	4758	France	2005	Human	B	B1	Cl2	Cl2.1	O25b:H4	fimH22		gyrA L83, N87	parC S80, E84
B125	SRS1274423	5176551	105	185329	4893	UK	2012	Human	C	C2	Cl3	Cl3.2	O25b:H4	fimH30	CTX-M-15	gyrA L83, N87	parC I80, V84
B12I1	SRS5403690	5158005	134	160682	4860	France	2005	Human	C	C2	Cl3	Cl3.2	O25b:H4	fimH30		gyrA L83, N87	parC I80, V84
B132	SRS1274422	5115728	67	216431	4775	UK	2012	Human	B	B5	Cl2	Cl2.1	O25b:H4	fimH22	CTX-M-1	gyrA S83, N87	parC S80, E84
B133	SRS1274421	5286586	122	174125	4987	UK	2012	Human	C	C2	Cl3	Cl3.2	O25b:H4	fimH30	CTX-M-15	gyrA L83, N87	parC I80, V84
B150	SRS1274420	5320621	136	185995	5034	UK	2012	Human	C	C2	Cl3	Cl3.2	O25b:H4	fimH30	CTX-M-15	gyrA L83, N87	parC I80, V84
B16	SRS1274419	5254298	134	155024	4967	UK	2012	Human	C	C2	Cl3	Cl3.2	O25b:H4	fimH30	CTX-M-15	gyrA L83, N87	parC I80, V84
B1A5	SRS5403675	5217708	142	160657	4937	France	2005	Human	C	C1	Cl3	Cl3.2	O25b:H4	fimH30		gyrA L83, N87	parC I80, V84
B1H12	SRS5403676	5173590	130	173800	4899	France	2005	Human	C	C1	Cl3	Cl3.2	O25b:H4	fimH30		gyrA L83, N87	parC I80, V84
B26	SRS1274417	5124722	102	180826	4805	UK	2012	Human	B	B4	Cl3	Cl3.1	O25b:H4			gyrA S83, N87	parC S80, E84
B2B2	SRS5403677	5232830	170	160846	4943	France	2005	Human	C	C1	Cl3	Cl3.2	O25b:H4	fimH30	CTX-M-14	gyrA L83, N87	parC I80, V84
B3	SRS1274416	5127628	67	272656	4809	UK	2012	Human	B	B4	Cl3	Cl3.1	O25b:H4	fimH22		gyrA S83, N87	parC S80, E84
B44	SRS1274413	5201130	69	284412	4921	UK	2012	Human	B	B4	Cl3	Cl3.1	O25b:H4	fimH22		gyrA S83, N87	parC S80, E84
B46	SRS1274414	4983199	85	187011	4664	UK	2012	Human	C	C1	Cl3	Cl3.2	O25b:H4	fimH30		gyrA L83, N87	parC I80, V84
B47	SRS1274412	5098575	122	191433	4789	UK	2012	Human	C	C1	Cl3	Cl3.2	O25b:H4	fimH30		gyrA L83, N87	parC I80, V84
B48	SRS1274411	5172436	114	143082	4876	UK	2012	Human	B	B5	Cl2	Cl2.1	O25b:H4	fimH22		gyrA S83, N87	parC S80, E84
B5	SRS1274410	5402070	167	173074	5105	UK	2012	Human	C	C2	Cl3	Cl3.2	O25b:H4	fimH30	CTX-M-15	gyrA L83, N87	parC I80, V84
B51	SRS1274409	5022381	60	181717	4691	UK	2012	Human	B	B4	Cl3	Cl3.1	O25b:H4	fimH22		gyrA S83, N87	parC S80, E84
B54	SRS1274408	5147556	95	135745	4832	UK	2012	Human	B	B4	Cl3	Cl3.1	O25b:H4	fimH22		gyrA S83, N87	parC S80, E84
B58	SRS1274407	5241072	116	164996	4926	UK	2012	Human	C	C2	Cl3	Cl3.2	O25b:H4	fimH30	CTX-M-15	gyrA L83, N87	parC I80, V84
B5B6	SRS5403689	5185566	111	180447	4879	France	2005	Human	C	C2	Cl3	Cl3.2	O25b:H4	fimH30	CTX-M-15	gyrA L83, N87	parC I80, V84
B65	SRS1274406	5198381	101	173780	4928	UK	2012	Human	C	C2	Cl3	Cl3.2	O25b:H4	fimH30	CTX-M-15	gyrA L83, N87	parC I80, V84
B71	SRS1274405	5165904	83	172368	4830	UK	2012	Human	B	B4	Cl3	Cl3.1	O25b:H4			gyrA S83, N87	parC S80, E84

B75	SRS1274404	5286100	128	175144	4983	UK	2012	Human	C	C2	Cl3	Cl3.2	O25b:H4	fimH30	CTX-M-15	gyrA L83, N87	parC I80, V84
B77	SRS1274403	5276229	118	194969	4980	UK	2012	Human	C	C2	Cl3	Cl3.2	O25b:H4	fimH30	CTX-M-15	gyrA L83, N87	parC I80, V84
B7S75	SRS3807985	5186331	131	162042	4919	USA	2017	Avian	B	B3	Cl2	Cl2.1	O25b:H4	fimH22-like		gyrA S83, N87	parC S80, E84
B89	SRS1274400	5327453	138	175130	5028	UK	2012	Human	C	C2	Cl3	Cl3.2	O25b:H4	fimH30	CTX-M-15	gyrA L83, N87	parC I80, V84
B94	SRS1274399	5276835	117	195284	4985	UK	2012	Human	C	C2	Cl3	Cl3.2	O25b:H4	fimH30	CTX-M-15	gyrA L83, N87	parC I80, V84
B95	SRS1274398	5195773	109	155110	4911	UK	2012	Human	C	C2	Cl3	Cl3.2	O25b:H4	fimH30	CTX-M-15	gyrA L83, N87	parC I80, V84
BLSE105-2012	SAMEA7758192	5107550	58	198064	4807	France	2012	Human	C	C3	Cl3	Cl3.2	O25b:H4	fimH30	CTX-M-15	gyrA L83, N87	parC I80, V84
BLSE108-2012	SAMEA7758193	5122535	85	172923	4793	France	2012	Human	C	C2	Cl3	Cl3.2	O25b:H4	fimH30	CTX-M-15	gyrA L83, N87	parC I80, V84
BLSE111-2012	SAMEA7758194	5283381	102	229734	5014	France	2012	Human	C	C2	Cl3	Cl3.2	O25b:H4	fimH30	CTX-M-15	gyrA L83, N87	parC I80, V84
BLSE130-2012	SAMEA7758196	5028678	84	136578	4699	France	2012	Human	A	A1	Cl1	Cl1.1	O16:H5	fimH41	CTX-M-14	gyrA L83, N87	parC S80, E84
BLSE13-2012	SAMEA7758195	5229825	65	203965	4977	France	2012	Human	C	C2	Cl3	Cl3.2	O25b:H4	fimH30	CTX-M-15	gyrA L83, N87	parC I80, V84
BLSE132-2012	SAMEA7758197	5101178	96	158869	4772	France	2012	Human	C	C1	Cl3	Cl3.2	O25b:H4	fimH30	CTX-M-27	gyrA L83, N87	parC I80, V84
BLSE136-2012	SAMEA7758198	5334206	95	159642	5032	France	2012	Human	C	C1	Cl3	Cl3.2	O25b:H4	fimH30	CTX-M-15	gyrA L83, N87	parC I80, V84
BLSE138-2012	SAMEA7758199	5225945	78	158868	4938	France	2012	Human	C	C2	Cl3	Cl3.2	O25b:H4	fimH30	CTX-M-15	gyrA L83, N87	parC I80, V84
BLSE140-2012	SAMEA7758200	5203772	104	181634	4908	France	2012	Human	A	A1	Cl1	Cl1.1	O16:H5	fimH41	CTX-M-15	gyrA L83, N87	parC S80, E84
BLSE157-2012	SAMEA7758201	5053688	89	186355	4725	France	2012	Human	A	A1	Cl1	Cl1.1	O16:H5	fimH41	CTX-M-15	gyrA L83, N87	parC S80, E84
BLSE158-2012	SAMEA7758202	5218906	80	191104	4914	France	2012	Human	C	C2	Cl3	Cl3.2	O25b:H4	fimH30	CTX-M-15	gyrA L83, N87	parC I80, V84
BLSE166-2012	SAMEA7758203	5147884	97	209323	4835	France	2012	Human	C	C2	Cl3	Cl3.2	O25b:H4	fimH30	CTX-M-15	gyrA L83, N87	parC I80, V84
BLSE2018-1	SAMEA7758204	5326195	82	230617	5041	France	2018	Human	C	C2	Cl3	Cl3.2	O25b:H4	fimH30	CTX-M-15	gyrA L83, N87	parC I80, V84
BLSE2018-101	SAMEA7758205	5349425	103	169482	5125	France	2018	Human	C	C2	Cl3	Cl3.3	O25b:H4	fimH30	CTX-M-15	gyrA L83, N87	parC I80, V84
BLSE2018-108	SAMEA7758206	5137004	67	231819	4881	France	2018	Human	A	A1	Cl1	Cl1.1	O16:H5	fimH41	CTX-M-15	gyrA L83, N87	parC S80, E84
BLSE2018-11	SAMEA7758207	5407085	62	219947	5189	France	2018	Human	C	C2	Cl3	Cl3.2	O25b:H4	fimH30	CTX-M-15	gyrA L83, N87	parC I80, V84
BLSE2018-110	SAMEA7758208	5121245	98	156958	4838	France	2018	Human	C	C1	Cl3	Cl3.2	O25b:H4	fimH30		gyrA L83, N87	parC I80, V84
BLSE2018-111	SAMEA7758209	5183090	100	173727	4863	France	2018	Human	C	C1	Cl3	Cl3.2	O25b:H4	fimH30	CTX-M-14	gyrA L83, N87	parC I80, V84
BLSE2018-117	SAMEA7758210	5305051	128	159030	5029	France	2018	Human	C	C2	Cl3	Cl3.2	O25b:H4	fimH30	CTX-M-15	gyrA L83, N87	parC I80, V84
BLSE2018-124	SAMEA7758211	5211901	98	165471	4921	France	2018	Human	C	C2	Cl3	Cl3.3	O25b:H4	fimH30	CTX-M-15	gyrA L83, N87	parC I80, V84
BLSE2018-126	SAMEA7758212	5224654	84	180367	4929	France	2018	Human	C	C2	Cl3	Cl3.2	O25b:H4	fimH30	CTX-M-15	gyrA L83, N87	parC I80, V84
BLSE2018-127	SAMEA7758213	5432789	148	168118	5181	France	2018	Human	C	C2	Cl3	Cl3.2	O25b:H4	fimH30	CTX-M-15	gyrA L83, N87	parC I80, V84
BLSE2018-130	SAMEA7758214	5083526	84	173718	4782	France	2018	Human	C	C2	Cl3	Cl3.2	O25b:H4	fimH30	CTX-M-15	gyrA L83, N87	parC I80, V84
BLSE2018-131	SAMEA7758215	5222498	126	169764	4954	France	2018	Human	C	C2	Cl3	Cl3.2	O25b:H4	fimH30	CTX-M-15	gyrA L83, N87	parC I80, V84
BLSE2018-132	SAMEA7758216	5244476	78	175143	4948	France	2018	Human	C	C2	Cl3	Cl3.2	O25b:H4	fimH30	CTX-M-15	gyrA L83, N87	parC I80, V84
BLSE2018-141	SAMEA7758217	5222570	112	182988	4898	France	2018	Human	C	C2	Cl3	Cl3.2	O25b:H4	fimH30	CTX-M-15	gyrA L83, N87	parC I80, V84
BLSE2018-142	SAMEA7758218	5042660	91	136872	4734	France	2018	Human	C	C1	Cl3	Cl3.2	O25b:H4	fimH30	CTX-M-27	gyrA L83, N87	parC I80, V84
BLSE2018-143	SAMEA7758219	5043812	78	186396	4724	France	2018	Human	C	C1	Cl3	Cl3.2	O25b:H4	fimH30	CTX-M-27	gyrA L83, N87	parC I80, V84
BLSE2018-144	SAMEA7758220	5056492	77	153459	4736	France	2018	Human	A	A1	Cl1	Cl1.1	O16:H5	fimH41	CTX-M-15	gyrA L83, N87	parC S80, E84
BLSE2018-150	SAMEA7758221	5158223	85	161362	4819	France	2018	Human	C	C1	Cl3	Cl3.2	O25b:H4	fimH30	CTX-M-27	gyrA L83, N87	parC I80, V84
BLSE2018-154	SAMEA7758222	5263527	87	166733	4990	France	2018	Human	C	C2	Cl3	Cl3.2	O25b:H4	fimH30	CTX-M-15	gyrA L83, N87	parC I80, V84
BLSE2018-155	SAMEA7758223	5233854	102	159030	4966	France	2018	Human	C	C1	Cl3	Cl3.2	O25b:H4	fimH30	CTX-M-27	gyrA L83, N87	parC I80, V84
BLSE2018-158	SAMEA7758224	5033420	64	173730	4746	France	2018	Human	C	C1	Cl3	Cl3.2	O25b:H4	fimH30	CTX-M-1	gyrA L83, N87	parC I80, V84
BLSE2018-159	SAMEA7758225	5232270	89	173508	4937	France	2018	Human	C	C2	Cl3	Cl3.2	O25b:H4	fimH30	CTX-M-15	gyrA L83, N87	parC I80, V84
BLSE2018-161	SAMEA7758226	5227924	90	158913	4948	France	2018	Human	C	C2	Cl3	Cl3.2	O25b:H4	fimH30	CTX-M-15	gyrA L83, N87	parC I80, V84
BLSE2018-166	SAMEA7758227	5311887	105	173729	5059	France	2018	Human	C	C2	Cl3	Cl3.3	O25b:H4	fimH30	CTX-M-15	gyrA L83, N87	parC I80, V84
BLSE2018-167	SAMEA7758228	5338276	102	181372	5103	France	2018	Human	C	C2	Cl3	Cl3.3	O25b:H4	fimH30	CTX-M-15	gyrA L83, N87	parC I80, V84
BLSE2018-169	SAMEA7758229	5018880	62	212606	4673	France	2018	Human	A	A1	Cl1	Cl1.1	O16:H5	fimH41	CTX-M-14	gyrA L83, N87	parC S80, E84
BLSE2018-170	SAMEA7758230	5168217	77	158855	4884	France	2018	Human	C	C1	Cl3	Cl3.2	O25b:H4	fimH30	CTX-M-27	gyrA L83, N87	parC I80, V84
BLSE2018-185	SAMEA7758231	5306826	81	193074	5068	France	2018	Human	C	C2	Cl3	Cl3.2	O25b:H4	fimH30	CTX-M-15	gyrA L83, N87	parC I80, V84
BLSE2018-195	SAMEA7758232	5334518	59	222695	5090	France	2018	Human	C	C2	Cl3	Cl3.3	O25b:H4	fimH30	CTX-M-15	gyrA L83, N87	parC I80, V84
BLSE2018-203	SAMEA7758233	5181045	69	231617	4922	France	2018	Human	C	C2	Cl3	Cl3.2	O25b:H4	fimH30	CTX-M-15	gyrA L83, N87	parC I80, V84
BLSE2018-205	SAMEA7758234	5108253	71	216335	4797	France	2018	Human	C	C1	Cl3	Cl3.2	O25b:H4	fimH30	CTX-M-27	gyrA L83, N87	parC I80, V84
BLSE2018-23	SAMEA7758235	5285715	74	211757	5028	France	2018	Human	C	C2	Cl3	Cl3.3	O25b:H4	fimH30	CTX-M-15	gyrA L83, N87	parC I80, V84
BLSE2018-25	SAMEA7758236	5103629	71	173732	4792	France	2018	Human	C	C2	Cl3	Cl3.2	O25b:H4	fimH30	CTX-M-15	gyrA L83, N87	parC I80, V84
BLSE2018-29	SAMEA7758237	5459064	67	206137	5258	France	2018	Human	C	C2	Cl3	Cl3.3	O25b:H4	fimH30	CTX-M-15	gyrA L83, N87	parC I80, V84
BLSE2018-3	SAMEA7758238	5046350	73	222619	4696	France	2018	Human	C	C1	Cl3	Cl3.2	O25b:H4	fimH30	CTX-M-27	gyrA L83, N87	parC I80, V84
BLSE2018-30	SAMEA7758239	5374015	94	180948	5148	France	2018	Human	C	C2	Cl3	Cl3.3	O25b:H4	fimH30	CTX-M-15	gyrA L83, N87	parC I80, V84
BLSE2018-45	SAMEA7758240	5081094	79	216568	4750	France	2018	Human	C	C1	Cl3	Cl3.2	O25b:H4	fimH30	CTX-M-27	gyrA L83, N87	parC I80, V84
BLSE2018-46	SAMEA7758241	4921391	59	159233	4631	France	2018	Human	C	C1	Cl3	Cl3.2		fimH30	CTX-M-1	gyrA L83, N87	parC I80, V84

BLSE2018-50	SAMEA7758242	5320291	57	236662	5029	France	2018	Human	C	C2	Cl3	Cl3.2	O25b:H4	fimH30	CTX-M-15	gyrA L83, N87	parC I80, V84
BLSE2018-59	SAMEA7758243	5188989	73	205057	4894	France	2018	Human	C	C1	Cl3	Cl3.2	O25b:H4	fimH30	CTX-M-27	gyrA L83, N87	parC I80, V84
BLSE2018-69	SAMEA7758244	5226981	61	189118	4967	France	2018	Human	C	C2	Cl3	Cl3.2	O25b:H4	fimH30	CTX-M-15	gyrA L83, N87	parC I80, V84
BLSE2018-70	SAMEA7758245	5221083	60	338260	4926	France	2018	Human	C	C2	Cl3	Cl3.2	O25b:H4	fimH30	CTX-M-15	gyrA L83, N87	parC I80, V84
BLSE2018-75	SAMEA7758246	4965558	58	249848	4644	France	2018	Human	A	A1	Cl1	Cl1.1	O16:H5	fimH41	CTX-M-15	gyrA L83, N87	parC S80, E84
BLSE2018-76	SAMEA7758247	5292930	57	227443	5043	France	2018	Human	C	C2	Cl3	Cl3.3	O25b:H4	fimH30	CTX-M-15	gyrA L83, N87	parC I80, V84
BLSE2018-85	SAMEA7758248	5052188	79	246069	4751	France	2018	Human	A	A1	Cl1	Cl1.1	O16:H5	fimH41	CTX-M-55	gyrA L83, N87	parC I80, V84
BLSE2018-86	SAMEA7758249	5292417	66	195263	5014	France	2018	Human	C	C2	Cl3	Cl3.3	O2:H4	fimH30	CTX-M-15	gyrA L83, N87	parC I80, V84
BLSE2018-89	SAMEA7758250	5009808	83	173729	4692	France	2018	Human	C	C1	Cl3	Cl3.2	O25b:H4	fimH30	CTX-M-27	gyrA L83, N87	parC I80, V84
BLSE2018-91	SAMEA7758251	4978926	54	191127	4660	France	2018	Human	B	B3	Cl2	Cl2.1	O25b:H4	fimH22	CTX-M-1	gyrA L83, N87	parC I80, E84
BLSE22-2012	SAMEA7758252	5101140	56	186399	4789	France	2012	Human	C	C2	Cl3	Cl3.2	O25b:H4	fimH30	CTX-M-15	gyrA L83, N87	parC I80, V84
BLSE23-2012	SAMEA7758253	5310136	84	207773	5047	France	2012	Human	C	C2	Cl3	Cl3.3	O25b:H4	fimH30	CTX-M-15	gyrA L83, N87	parC I80, V84
BLSE24-2012	SAMEA7758254	5102061	55	186358	4797	France	2012	Human	C	C2	Cl3	Cl3.2	O25b:H4	fimH30	CTX-M-15	gyrA L83, N87	parC I80, V84
BLSE26-2012	SAMEA7758255	5118018	121	78628	4844	France	2012	Human	C	C1	Cl3	Cl3.2	O25b:H4	fimH30	CTX-M-27	gyrA L83, N87	parC I80, V84
BLSE44-2012	SAMEA7758256	5038228	79	172924	4689	France	2012	Human	C	C2	Cl3	Cl3.2	O25b:H4	fimH30	CTX-M-15	gyrA L83, N87	parC I80, V84
BLSE45-2012	SAMEA7758257	5221412	102	191127	4938	France	2012	Human	C	C2	Cl3	Cl3.2	O25b:H4	fimH30	CTX-M-15	gyrA L83, N87	parC I80, V84
BLSE47-2012	SAMEA7758258	5217920	61	188340	4962	France	2012	Human	C	C2	Cl3	Cl3.3	O25b:H4	fimH30	CTX-M-15	gyrA L83, N87	parC I80, V84
BLSE48-2012	SAMEA7758259	5084541	76	133298	4793	France	2012	Human	C	C1	Cl3	Cl3.2	O25b:H4	fimH30	CTX-M-14	gyrA L83, N87	parC I80, V84
BLSE65-2012	SAMEA7758260	5117228	56	173688	4821	France	2012	Human	C	C2	Cl3	Cl3.2	O25b:H4	fimH30	CTX-M-15	gyrA L83, N87	parC I80, V84
BLSE67-2012	SAMEA7758261	5255522	89	170267	4967	France	2012	Human	C	C1	Cl3	Cl3.2	O25b:H4	fimH30		gyrA L83, N87	parC I80, V84
BLSE71-2012	SAMEA7758262	5171087	99	159643	4852	France	2012	Human	C	C2	Cl3	Cl3.2	O25b:H4	fimH30-like	CTX-M-15	gyrA L83, N87	parC I80, V84
BLSE78-2012	SAMEA7758263	5160028	96	158810	4882	France	2012	Human	C	C1	Cl3	Cl3.2	O25b:H4	fimH30	CTX-M-14	gyrA L83, N87	parC I80, V84
BLSE81-2012	SAMEA7758264	5269769	90	184254	5018	France	2012	Human	C	C2	Cl3	Cl3.2	O25b:H4	fimH30	CTX-M-15	gyrA L83, N87	parC I80, V84
BLSE96-2012	SAMEA7758265	5466792	183	153455	5245	France	2012	Human	C	C2	Cl3	Cl3.3	O25b:H4	fimH30	CTX-M-15	gyrA L83, N87	parC I80, V84
BLSE97-2012	SAMEA7758266	5314457	105	158869	5049	France	2012	Human	C	C2	Cl3	Cl3.3	O25b:H4	fimH30	CTX-M-15	gyrA L83, N87	parC I80, V84
BLSE98-2012	SAMEA7758267	5273544	95	222117	5005	France	2012	Human	C	C2	Cl3	Cl3.3	O25b:H4	fimH30	CTX-M-15	gyrA L83, N87	parC I80, V84
BRG128	DRR092887	4994439	61	229842	4675	Japan	2014	Human	C	C1	Cl3	Cl3.2	O25b:H4	fimH30		gyrA L83, N87	parC I80, V84
BRG137	DRR092868	5105169	79	229891	4772	Japan	2014	Human	I	I2	Cl3	Cl3.2	O25b:H4	fimH54		gyrA L83, N87	parC S80, E84
BRG145	DRR092889	5160489	110	320431	4832	Japan	2014	Human	C	C2	Cl3	Cl3.2	O25b:H4	fimH30		gyrA L83, N87	parC I80, V84
BRG21	DRR092885	5136831	122	229828	4828	Japan	2014	Human	B	B4	Cl3	Cl3.1	O25b:H4	fimH22-like		gyrA S83, N87	parC S80, E84
BRG210	DRR092869	5219023	81	281401	4933	Japan	2014	Human	I	I2	Cl3	Cl3.2	O25b:H4	fimH54		gyrA L83, N87	parC S80, E84
BRG214	DRR092894	4978246	76	238743	4627	Japan	2014	Human	A	A1	Cl1	Cl1.1	O16:H5	fimH41	CTX-M-14	gyrA L83, N87	parC S80, E84
BRG23	DRR051030	5131445	108	139275	4810	Japan	2014	Human	C	C2	Cl3	Cl3.2	O25b:H4	fimH30	CTX-M-15	gyrA L83, N87	parC I80, V84
BRG247	DRR092891	5182197	114	212907	4915	Japan	2014	Human	C	C1	Cl3	Cl3.2	O25b:H4	fimH30	CTX-M-27	gyrA L83, N87	parC I80, V84
BRG342	DRR092893	5215874	62	303973	4933	Japan	2014	Human	C	C1	Cl3	Cl3.2	O25b:H4	fimH99		gyrA L83, N87	parC I80, V84
BRG77	DRR092886	5015155	81	222857	4674	Japan	2014	Human	A	A1	Cl1	Cl1.1	O16:H5	fimH41		gyrA L83, N87	parC I80, E84
BZH101	SAMEA7758268	5029901	73	209219	4695	France	2019	Human	B	B4	Cl3	Cl3.1	O2:H4	fimH22		gyrA S83, N87	parC S80, E84
BZH106	SAMEA7758269	5311945	141	107029	5082	France	2019	Human	C	C1	Cl3	Cl3.2	O25b:H4	fimH30	CTX-M-27	gyrA L83, N87	parC I80, V84
BZH109	SAMEA7758270	5118745	98	143834	4753	France	2019	Human	A	A1	Cl1	Cl1.1	O16:H5	fimH41	CTX-M-27	gyrA L83, N87	parC S80, E84
C001	SRR933345	5145462	108	191083	4848	USA	2010	Human	I	I4	Cl3	Cl3.2	O25b:H4	fimH30		gyrA S83, N87	parC S80, E84
C17	SRS6410199	5023803	78	148329	4680	Switzerland	2020	Feline	C	C1	Cl3	Cl3.2	O25b:H4	fimH30	CTX-M-27	gyrA L83, N87	parC I80, V84
C23	SRS5403691	5170210	198	160616	4818	France	2012	Human	C	C2	Cl3	Cl3.2	O25b:H4	fimH30		gyrA L83, N87	parC I80, V84
C5	SRS5403687	5176038	169	180447	4821	France	2012	Human	C	C1	Cl3	Cl3.2	O25b:H4	fimH30	CTX-M-27	gyrA L83, N87	parC I80, V84
C55	SAMEA7758271	5068945	61	223421	4715	France	2015	Human	B	B3	Cl2	Cl2.2	O25b:H4	fimH22		gyrA L83, N87	parC S80, E84
C67	SAMEA7758272	5211799	60	202602	4879	France	2015	Human	B	B3	Cl2	Cl2.2	O25b:H4	fimH22		gyrA L83, N87	parC S80, E84
C68	SAMEA7758273	5210709	63	136819	4882	France	2015	Human	B	B3	Cl2	Cl2.2	O25b:H4	fimH22		gyrA L83, N87	parC S80, E84
C92	SAMEA7758274	5273469	69	191606	4906	France	2015	Human	B	B3	Cl2	Cl2.2	O25b:H4	fimH22		gyrA S83, N87	parC S80, E84
cam_1071	SRR2970752	4964649	90	188405	4555	Cambodia	2008	Human	A	A1	Cl1	Cl1.1	O16:H5	fimH41	CTX-M-14	gyrA S83, N87	parC S80, E84
cam_1439	SRR2970749	5237208	101	119602	4932	Cambodia	2009	Human	C	C1	Cl3	Cl3.2	O25b:H4	fimH30	CTX-M-14	gyrA L83, N87	parC I80, V84
cam_1814	SRR2970719	5048970	90	159641	4711	Cambodia	2010	Human	C	C1	Cl3	Cl3.2	O25b:H4	fimH30	CTX-M-27	gyrA L83, N87	parC I80, V84
cam_1920	SRR2970753	5465993	105	191083	5170	Cambodia	2010	Human	B	B5	Cl3	Cl3.1	O25b:H4	fimH298		gyrA L83, N87	parC S80, E84
cam_2254	SRR2970774	5289510	144	171274	4981	Cambodia	2010	Human	B	B5	Cl3	Cl3.1	O25b:H4	fimH298		gyrA L83, N87	parC S80, E84
cam_2830	SRR2970775	5289361	109	166176	4962	Cambodia	2009	Human	B	B5			O25b:H4	fimH298		gyrA L83, N87	parC S80, E84
cam_2853	SRR2970750	5137434	91	160340	4842	Cambodia	2009	Human	B	B5	Cl3	Cl3.1	O25b:H4	fimH22		gyrA L83, N87	parC S80, E84
cam_2917	SRR2970776	5219396	87	159643	4890	Cambodia	2011	Human	C	C2	Cl3	Cl3.2	O25b:H4	fimH30	CTX-M-15	gyrA L83, N87	parC I80, V84
cam_3162	SRR2970777	4952855	89	171124	4612	Cambodia	2011	Human	A	A1	Cl1	Cl1.1	O25b:H4	fimH41		gyrA S83, N87	parC S80, E84

CD340	SRR933467	5118871	73	206102	4815	USA	2005	Monkey	C	C1	Cl3	Cl3.2	O25b:H4	fimH30		gyrA L83, N87	parC I80, V84
CD400	SRR933351	5251975	76	173335	4926	USA	1992	Human	B	B4	Cl3	Cl3.1	O25b:H4	fimH22		gyrA S83, N87	parC S80, E84
CD466	SRR933353	5345114	109	241915	5055	USA	1990	Avian	B	B3	Cl2	Cl2.4	O25b:H4	fimH22		gyrA S83, N87	parC S80, E84
CD467	SRR933355	5135346	56	242560	4773	USA	2009	Avian	B	B3	Cl2	Cl2.2	O25b:H4	fimH22		gyrA S83, N87	parC S80, E84
CD505	SRR933359	5209749	156	109366	4928	USA	1983	Avian	B	B2	Cl2	Cl2.1	O25b:H4	fimH22		gyrA S83, N87	parC S80, E84
CEA618S	SAMEA7758275	5145528	55	227897	4853	France	2016	Human	C	C1	Cl3	Cl3.2	O25b:H4	fimH30		gyrA L83, N87	parC I80, V84
CEA704S	SAMEA7758276	5120604	48	224857	4830	France	2016	Human	B	B4	Cl3	Cl3.1	O25b:H4	fimH22		gyrA S83, N87	parC S80, E84
CES103C	SRS5403682	5103395	182	137128	4775	France	2007	Human	C	C1	Cl3	Cl3.2	O25b:H4	fimH30		gyrA L83, N87	parC I80, V84
CES131C	SRS5403663	5239648	171	190918	4959	France	2008	Human	B	B4	Cl3	Cl3.2	O25b:H4	fimH30		gyrA S83, N87	parC S80, E84
CES164C	SRS5403685	5048692	125	190918	4730	France	2009	Human	C	C1	Cl3	Cl3.2	O25b:H4	fimH30		gyrA L83, N87	parC I80, V84
CES9C	SRS5403683	5102214	143	173815	4781	France	2007	Human	C	C1	Cl3	Cl3.2	O25b:H4	fimH30		gyrA L83, N87	parC I80, V84
CHOG1	SAMEA7758277	5282683	81	188349	5013	Guyana	2013	Human	C	C2	Cl3	Cl3.3	O25b:H4	fimH30	CTX-M-15	gyrA L83, N87	parC I80, V84
CHOG36	SAMEA7758278	5125443	93	191127	4806	Guyana	2013	Human	C	C2	Cl3	Cl3.2	O25b:H4	fimH30	CTX-M-15	gyrA L83, N87	parC I80, V84
CNR1337	SAMEA7758279	5184213	102	152122	4881	France	2016	Human	C	C1	Cl3	Cl3.2	O25b:H4	fimH30	CTX-M-27	gyrA L83, N87	parC I80, V84
CNR1492	SAMEA7758280	5113785	65	209445	4771	France	2016	Human	C	C1	Cl3	Cl3.2	O25b:H4	fimH30	CTX-M-27	gyrA L83, N87	parC I80, V84
CNR1613	SAMEA7758281	5059545	142	106037	4708	France	2016	Human	C	C1	Cl3	Cl3.2	O25b:H4	fimH30	CTX-M-27	gyrA L83, N87	parC I80, V84
CNR2034	SAMEA7758282	5088803	106	117050	4790	France	2017	Human	C	C1	Cl3	Cl3.2	O25b:H4	fimH30	CTX-M-27	gyrA L83, N87	parC I80, V84
CNR2195	SAMEA7758283	5177250	134	225862	4871	France	2017	Human	C	C1	Cl3	Cl3.2	O25b:H4	fimH30	CTX-M-27	gyrA L83, N87	parC I80, V84
CNR2215	SAMEA7758284	5169226	66	257547	4872	France	2017	Human	A	A1	Cl1	Cl1.1	O16:H5	fimH41	CTX-M-15	gyrA L83, N87	parC S80, E84
CNR2225	SAMEA7758285	5013270	84	222567	4644	France	2017	Human	A	A1	Cl1	Cl1.1	O16:H4	fimH41		gyrA S83, N87	parC S80, E84
CNR2363	SAMEA7758286	5246590	73	427380	4919	La Reunion	2018	Human	B	B5	Cl2	Cl2.1	O25b:H4	fimH22		gyrA S83, N87	parC S80, E84
CNR2477	SAMEA7758287	5073131	41	284518	4742	France	2018	Human	B	B4	Cl3	Cl3.1	O25b:H4	fimH30		gyrA S83, N87	parC S80, E84
CNR2492	SAMEA7758288	5150247	74	208193	4827	France	2018	Human	C	C1	Cl3	Cl3.2	O25b:H4	fimH30	CTX-M-27	gyrA L83, N87	parC I80, V84
CNR2532	SAMEA7758289	5101618	78	173944	4747	France	2018	Human	C	C1	Cl3	Cl3.2	O25b:H4	fimH30	CTX-M-27	gyrA L83, N87	parC I80, V84
CNR2549	SAMEA7758290	5071490	91	176247	4771	France	2018	Human	A	A1	Cl1	Cl1.1	O16:H5	fimH41		gyrA S83, N87	parC S80, E84
CNR2557	SAMEA7758291	5324589	103	220271	5060	France	2018	Human	C	C2	Cl3	Cl3.3	O25b:H4	fimH30	CTX-M-15	gyrA L83, N87	parC I80, V84
CNR2885	SAMEA7758292	5036686	65	229903	4695	France	2018	Human	C	C1	Cl3	Cl3.2	O25b:H4	fimH30	CTX-M-27	gyrA L83, N87	parC I80, V84
CNR3021	SAMEA7758293	5279654	71	231809	5034	France	2019	Human	C	C2	Cl3	Cl3.2	O25b:H4			gyrA L83, N87	parC I80, V84
CNR3098	SAMEA7758294	5097175	118	140480	4785	France	2019	Human	C	C1	Cl3	Cl3.2	O25b:H4	fimH30	CTX-M-27	gyrA L83, N87	parC I80, V84
CNR3102	SAMEA7758295	5179563	87	186375	4883	France	2019	Human	C	C2	Cl3	Cl3.2	O25b:H4	fimH30	CTX-M-15	gyrA L83, N87	parC I80, V84
CNR622	SAMEA7758296	5206748	180	182224	4855	France	2014	Human	C	C1	Cl3	Cl3.2	O25b:H4	fimH30	CTX-M-27	gyrA L83, N87	parC I80, V84
CNR963	SAMEA7758297	5175428	110	222847	4890	France	2015	Human	C	C1	Cl3	Cl3.2	O25b:H4	fimH30	CTX-M-174	gyrA L83, N87	parC I80, V84
CR48	SAMN03798494	5185688	63	179956	4914	Australia	2014	Human	B	B4	Cl3	Cl3.1	O25b:H4	fimH22		gyrA S83, N87	parC S80, E84
CU799	SRR933485	5073067	162	50150	4778	USA	2008	Feline	C	C1	Cl3	Cl3.2	O25b:H4	fimH30		gyrA L83, N87	parC I80, V84
CVL137	SAMEA7758298	5104687	119	137799	4760	France	2019	Human	C	C1	Cl3	Cl3.2	O25b:H4	fimH30	CTX-M-27	gyrA L83, N87	parC I80, V84
CVL142	SAMEA7758299	5135769	92	191640	4769	France	2019	Human	A	A1	Cl1	Cl1.1	O16:H5	fimH41	CTX-M-27	gyrA L83, N87	parC S80, E84
E005	SAMN06106904	5304511	112	173799	5059	South Africa	2013	Human	C	C1	Cl3	Cl3.2	O25b:H4	fimH30	CTX-M-15	gyrA L83, N87	parC I80, V84
E009	SAMN06106905	5297268	114	192064	5100	South Africa	2013	Human	C	C1	Cl3	Cl3.2	O25b:H4	fimH30	CTX-M-15	gyrA L83, N87	parC I80, V84
E011	SAMN06106889	5338648	183	157722	5148	South Africa	2013	Human	C	C2	Cl3	Cl3.2	O25b:H4	fimH30	CTX-M-15	gyrA L83, N87	parC I80, V84
E013	SAMN06106834	5069612	125	180463	4768	South Africa	2013	Human	C	C1	Cl3	Cl3.2	O25b:H4	fimH30	CTX-M-27	gyrA L83, N87	parC I80, V84
E021	SAMN06106835	5174825	164	166728	4878	South Africa	2013	Human	C	C2	Cl3	Cl3.2	O25b:H4	fimH30	CTX-M-15	gyrA L83, N87	parC I80, V84
E056	SAMN06106850	5189204	186	153548	4919	South Africa	2013	Human	C	C2	Cl3	Cl3.2	O25b:H4	fimH30	CTX-M-15	gyrA L83, N87	parC I80, V84
E058	SAMN06106907	5138889	96	199691	4893	South Africa	2013	Human	C	C2	Cl3	Cl3.2	O25b:H4	fimH30	CTX-M-15	gyrA L83, N87	parC I80, V84
E060	SAMN06106908	5188955	123	185789	4920	South Africa	2013	Human	C	C2	Cl3	Cl3.2	O25b:H4	fimH30	CTX-M-15	gyrA L83, N87	parC I80, V84
E062	SAMN06106851	5125972	125	159178	4869	South Africa	2013	Human	C	C2	Cl3	Cl3.2	O25b:H4	fimH30	CTX-M-15	gyrA L83, N87	parC I80, V84
E063	SAMN06106840	5396374	199	151395	5170	South Africa	2013	Human	C	C2	Cl3	Cl3.2	O25b:H4	fimH30	CTX-M-15	gyrA L83, N87	parC I80, V84
E4	SRR3143605	5137179	176	163492	4830	China	2011	Human	C	C1	Cl3	Cl3.2	O25b:H4	fimH30	CTX-M-14	gyrA L83, N87	parC I80, V84
Ec24	DRR051042	5054256	77	182466	4705	Canada	2008	Human	C	C1	Cl3	Cl3.2	O25b:H4	fimH30	CTX-M-27	gyrA L83, N87	parC I80, V84
Ec58	DRR051044	5267108	90	159030	4961	Canada	2009	Human	C	C2	Cl3	Cl3.2	O25b:H4	fimH30	CTX-M-15	gyrA L83, N87	parC I80, V84
EC958	GCA_000285655.3	5287357	194	82668	4956	UK	2005	Human	C	C2	Cl3	Cl3.2	O25b:H4	fimH30	CTX-M-15	gyrA L83, N87	parC I80, V84
EcAZ156	DRR051046	5091385	85	146319	4777	Thailand	2013	Human	C	C1	Cl3	Cl3.2	O25b:H4	fimH30	CTX-M-27	gyrA L83, N87	parC I80, V84
ECNZ35	DRR051040	5272683	92	159030	4985	New Zealand	2010	Human	C	C1	Cl3	Cl3.2	O25b:H4	fimH30		gyrA L83, N87	parC I80, V84
EcSA01	DRR051041	5180608	76	158854	4864	South Africa	2008	Human	C	C1	Cl3	Cl3.2	O25b:H4	fimH30	CTX-M-14	gyrA L83, N87	parC I80, V84
ErtS	ERS1031999	5397004	6	5082442	5140	France	2012	Human	C	C2	Cl3	Cl3.2	O25b:H4	fimH30	CTX-M-15	gyrA L83, N87	parC I80, V84

ESC_BA9710AA	ERS193930	5211500	184	191718	4871	Czech Republic	2011	Wild avian	C	C2	Cl3	Cl3.2	O25b:H4	fimH30	CTX-M-15	gyrA L83, N87	parC I80, V84
ESC_BA9711AA	ERS193929	5287682	203	325756	4932	Czech Republic	2011	Wild avian	C	C2	Cl3	Cl3.2	O25b:H4	fimH30	CTX-M-15	gyrA L83, N87	parC I80, V84
ESC_BA9712AA	ERS193928	5141095	161	339975	4744	Germany	2007	Avian	B	B5	Cl2	Cl2.1	O25b:H4	fimH22		gyrA S83, N87	parC S80, E84
ESC_BA9713AA	ERS193927	5092069	172	214468	4737	Germany	2011	Canine	C	C1	Cl3	Cl3.2	O25b:H4	fimH30	CTX-M-27	gyrA L83, N87	parC I80, V84
ESC_BA9714AA	ERS193926	5232200	109	319751	4939	Germany	2011	Canine	C	C2	Cl3	Cl3.2	O25b:H4	fimH30	CTX-M-15	gyrA L83, N87	parC I80, V84
ESC_BA9715AA	ERS193925	4894521	117	229914	4509	Germany	2010	Canine	A	A1	Cl1	Cl1.1	O16:H5	fimH41	CTX-M-14	gyrA L83, N87	parC I80, E84
ESC_BA9716AA	ERS193924	5178848	113	322807	4848	Germany	2010	Canine	C	C2	Cl3	Cl3.2	O25b:H4	fimH30	CTX-M-15	gyrA L83, N87	parC I80, V84
ESC_BA9717AA	ERS193923	5041408	136	214468	4682	France	2010	Canine	C	C1	Cl3	Cl3.2	O25b:H4	fimH30	CTX-M-27	gyrA L83, N87	parC I80, V84
ESC_BA9718AA	ERS193922	5178848	107	322807	4850	Germany	2010	Feline	C	C2	Cl3	Cl3.2	O25b:H4	fimH30	CTX-M-15	gyrA L83, N87	parC I80, V84
ESC_BA9719AA	ERS193921	5041696	138	187704	4679	Germany	2010	Canine	C	C1	Cl3	Cl3.2	O25b:H4	fimH30	CTX-M-27	gyrA L83, N87	parC I80, V84
ESC_BA9720AA	ERS193920	5129655	155	202902	4807	Netherlands	2010	Canine	C	C1	Cl3	Cl3.2	O25b:H4	fimH30	CTX-M-27	gyrA L83, N87	parC I80, V84
ESC_BA9721AA	ERS193919	4816232	117	279812	4429	Germany	2010	Canine	A	A1	Cl1	Cl1.1	O16:H5	fimH41	CTX-M-14	gyrA L83, N87	parC I80, E84
ESC_BA9722AA	ERS193918	4999823	106	191612	4680	Germany	2010	Feline	C	C2	Cl3	Cl3.2		fimH30	CTX-M-15	gyrA L83, N87	parC I80, V84
ESC_BA9723AA	ERS193917	4818471	121	279684	4422	Germany	2010	Canine	A	A1	Cl1	Cl1.1	O16:H5	fimH41	CTX-M-14	gyrA L83, N87	parC I80, E84
ESC_BA9724AA	ERS193916	5204871	143	202902	4892	Denmark	2010	Canine	C	C1	Cl3	Cl3.2	O25b:H4	fimH30	CTX-M-55	gyrA L83, N87	parC I80, V84
ESC_BA9725AA	ERS193915	5041668	101	220895	4654	Germany	2010	Feline	B	B5	Cl3	Cl3.1	O25b:H4	fimH298-like		gyrA S83, N87	parC S80, E84
ESC_BA9726AA	ERS193914	5328155	107	429826	4994	Germany	2010	Wild avian	B	B1	Cl2	Cl2.3	O25b:H4	fimH22	CTX-M-2	gyrA S83, N87	parC S80, E84
ESC_BA9727AA	ERS193913	5661754	225	159172	5412	Germany	2010	Canine	C	C2			O25b:H4	fimH30	CTX-M-9	gyrA L83, N87	parC I80, V84
ESC_BA9734AA	ERS193906	5068454	154	223133	4728	Italy	2010	Canine	C	C1	Cl3	Cl3.2	O25b:H4	fimH30	CTX-M-1	gyrA L83, N87	parC I80, V84
ESC_BA9735AA	ERS193905	5177496	126	294970	4823	Italy	2010	Feline	B	B1	Cl2	Cl2.1	O25b:H4	fimH22		gyrA S83, N87	parC S80, E84
ESC_BA9736AA	ERS193904	5328167	106	275200	5000	Netherlands	2010	Feline	B	B5	Cl3	Cl3.1	O25b:H4	fimH298	CTX-M-2	gyrA S83, N87	parC S80, E84
ESC_BA9737AA	ERS193903	5040230	149	191608	4700	Italy	2009	Canine	C	C1	Cl3	Cl3.2	O25b:H4	fimH30		gyrA L83, N87	parC I80, V84
ESC_BA9738AA	ERS193902	5030389	108	338937	4695	Italy	2009	Canine	C	C1	Cl3	Cl3.2	O25b:H4	fimH30		gyrA L83, N87	parC I80, V84
ESC_BA9739AA	ERS193901	4929264	93	534973	4589	Austria	2009	Feline	B	B5	Cl2	Cl2.1	O25b:H4	fimH22		gyrA S83, N87	parC S80, E84
ESC_BA9740AA	ERS193900	4886729	132	279409	4503	Germany	2009	Canine	A	A1	Cl1	Cl1.1	O16:H5	fimH41	CTX-M-14	gyrA L83, N87	parC I80, E84
ESC_BA9741AA	ERS193899	5159963	178	327432	4824	Germany	2009	Canine	C	C2	Cl3	Cl3.2	O25b:H4	fimH30	CTX-M-15	gyrA L83, N87	parC I80, V84
ESC_BA9742AA	ERS193898	5217180	166	204989	4903	Italy	2009	Canine	C	C2	Cl3	Cl3.2	O25b:H4	fimH30	CTX-M-15	gyrA L83, N87	parC I80, V84
ESC_BA9743AA	ERS193897	4752443	98	294229	4377	Germany	2009	Canine	A	A1	Cl1	Cl1.1	O16:H5	fimH41	CTX-M-14	gyrA L83, N87	parC I80, E84
ESC_BA9744AA	ERS193896	4717005	105	261183	4340	Germany	2009	Canine	A	A1	Cl1	Cl1.1	O16:H5	fimH41	CTX-M-14	gyrA L83, N87	parC I80, E84
ESC_BA9745AA	ERS193895	5071642	136	222362	4725	Denmark	2009	Canine	C	C1	Cl3	Cl3.2	O25b:H4	fimH30	CTX-M-27	gyrA L83, N87	parC I80, V84
ESC_BA9746AA	ERS193894	5217799	135	202902	4883	France	2009	Canine	C	C2	Cl3	Cl3.2	O25b:H4	fimH30	CTX-M-15	gyrA L83, N87	parC I80, V84
ESC_BA9747AA	ERS193893	5218079	146	202901	4882	Netherlands	2009	Canine	C	C2	Cl3	Cl3.2	O25b:H4	fimH30	CTX-M-15	gyrA L83, N87	parC I80, V84
ESC_BA9748AA	ERS193892	5277018	218	186273	4948	Germany	2009	Canine	C	C2	Cl3	Cl3.2	O25b:H4	fimH30	CTX-M-15	gyrA L83, N87	parC I80, V84
ESC_BA9749AA	ERS193891	5133501	106	319752	4821	Spain	2008	Canine	C	C2	Cl3	Cl3.2	O25b:H4	fimH30	CTX-M-15	gyrA L83, N87	parC I80, V84
ESC_BA9750AA	ERS193890	5229208	155	336228	4901	Denmark	2008	Canine	C	C2	Cl3	Cl3.2	O25b:H4	fimH30	CTX-M-15	gyrA L83, N87	parC I80, V84
ESC_BA9751AA	ERS193939	5063792	158	298843	4676	Germany	2008	Canine	B	B3	Cl2	Cl2.1	O25b:H4	fimH22		gyrA S83, N87	parC S80, E84
ESC_BA9753AA	ERS193937	5123043	154	276157	4820	Serbia	2011	Wild avian	C	C1	Cl3	Cl3.2	O25b:H4	fimH30	CTX-M-15	gyrA L83, N87	parC I80, V84
ESC_BA9754AA	ERS193936	5304786	245	175528	4999	Serbia	2011	Wild avian	C	C2	Cl3	Cl3.2	O25b:H4	fimH30	CTX-M-15	gyrA L83, N87	parC I80, V84
ESC_BA9755AA	ERS193935	5233810	245	204989	4898	Czech Republic	2011	Wild avian	C	C2	Cl3	Cl3.2	O25b:H4	fimH30	CTX-M-15	gyrA L83, N87	parC I80, V84
ESC_BA9756AA	ERS193934	5067930	99	319368	4721	Czech Republic	2011	Wild avian	C	C2	Cl3	Cl3.2	O25b:H4	fimH30	CTX-M-15	gyrA L83, N87	parC I80, V84
ESC_BA9757AA	ERS193933	5078347	104	191608	4735	Czech Republic	2011	Wild avian	C	C2	Cl3	Cl3.2	O25b:H4	fimH30	CTX-M-15	gyrA L83, N87	parC I80, V84
ESC_BA9758AA	ERS193932	5068200	104	319368	4721	Czech Republic	2011	Wild avian	C	C2	Cl3	Cl3.2	O25b:H4	fimH30	CTX-M-15	gyrA L83, N87	parC I80, V84
ESC_BA9759AA	ERS193931	5322400	210	175528	4979	Czech Republic	2011	Wild avian	C	C2	Cl3	Cl3.2	O25b:H4	fimH30	CTX-M-15	gyrA L83, N87	parC I80, V84
ESC_CA0832AA	SAMN02228549	5011674	509	22922	4778	Australia	2008	Canine	C	C2	Cl3	Cl3.2	O25b:H4	fimH30	CTX-M-15	gyrA L83, N87	parC I80, V84
ESC_CA0859AA	SAMN02228522	5165870	340	49576	4869	USA	2008	Feline	C	C1	Cl3	Cl3.2	O25b:H4	fimH30		gyrA L83, N87	parC I80, V84
ESC_CA0873AA	SAMN02228508	5343207	436	29609	5077	USA	2001	Avian	B	B5	Cl2	Cl2.4	O25b:H4	fimH22		gyrA S83, N87	parC S80, E84
ESC_CA9001AA	SAMN04381845	5219043	2	5073822	4935	USA	2002	Feline	C	C1	Cl3	Cl3.2	O25b:H4	fimH30		gyrA S83, N87	parC S80, E84
ESC_DA1317AA	ERP015467	5667662	268	38642	5379	Denmark	2012	Avian	B	B3	Cl2	Cl2.3	O25b:H4	fimH22		gyrA S83, N87	parC S80, E84

ESC_DA1968AA	SAMEA92078668	5255653	142	114349	4942	Spain	2008	Avian	B	B3	Cl2	Cl2.3	O25b:H4	fimH22	CTX-M-9	gyrA A83, N87	parC S80, E84
ESC_DA1990AA	SAMEA92093668	5173369	132	191707	4791	Spain	2011	Avian	B	B3	Cl2	Cl2.2	O25b:H4	fimH22		gyrA S83, N87	parC S80, E84
ESC_DA4599AA	SAMN04273116	5069545	111	217403	4737	Serbia	2007	Wild avian	C	C1	Cl3	Cl3.2	O25b:H4	fimH30	CTX-M-27	gyrA L83, N87	parC I80, V84
ESC_DA4600AA	SAMN04273115	5066883	111	216137	4715	Czech Republic	2007	Wild avian	C	C1	Cl3	Cl3.2	O25b:H4	fimH30	CTX-M-27	gyrA L83, N87	parC I80, V84
ESC_DA4603AA	SAMN04273112	5126650	144	334434	4836	Serbia	2010	Wild avian	C	C1	Cl3	Cl3.2	O25b:H4	fimH30	CTX-M-15	gyrA L83, N87	parC I80, V84
ESC_DA4604AA	SAMN04273111	5298845	172	285473	4962	Czech Republic	2010	Wild avian	C	C2	Cl3	Cl3.2	O25b:H4	fimH30	CTX-M-15	gyrA L83, N87	parC I80, V84
ESC_DA4605AA	SAMN04273110	5098893	122	191009	4787	USA	2012	Wild avian	C	C1	Cl3	Cl3.2	O25b:H4	fimH30	CTX-M-27	gyrA L83, N87	parC I80, V84
ESC_EA0976AA	SAMN04992373	5324797	93	265378	4991	USA	2007	Avian	B	B3	Cl2	Cl2.3	O25b:H4	fimH22		gyrA S83, N87	parC S80, E84
ESC_EA1004AA	SAMN05360006	5158158	87	276255	4848	USA	2008	Feline	C	C1	Cl3	Cl3.2	O25b:H4	fimH30		gyrA L83, N87	parC I80, V84
ESC_EA1005AA	SAMN05360005	5156183	81	268228	4843	USA	2008	Feline	C	C1	Cl3	Cl3.2	O25b:H4	fimH30		gyrA L83, N87	parC I80, V84
ESC_EA1007AA	SAMN05360004	5143603	78	276255	4837	USA	2008	Canine	C	C1	Cl3	Cl3.2	O25b:H4	fimH30		gyrA L83, N87	parC I80, V84
ESC_EA1018AA	SAMN05360003	5154269	78	276327	4845	USA	2008	Canine	C	C1	Cl3	Cl3.2	O25b:H4	fimH30		gyrA L83, N87	parC I80, V84
ESC_EA1023AA	SAMN05360016	5331710	166	185705	5058	USA	2013		C	C2	Cl3	Cl3.3	O25b:H4	fimH30	CTX-M-15	gyrA L83, N87	parC I80, V84
ESC_EA1816AA	SAMD00053087	5140931	450	30960	4804	Japan	2016	Water	A	A1	Cl1	Cl1.1	O16:H5	fimH41		gyrA L83, N87	parC S80, E84
ESC_FA9052AA	SAMEA92140918	5310886	91	313295	4966	Spain	2016	Avian	B	B3	Cl2	Cl2.2	O25b:H4	fimH22		gyrA L83, N87	parC S80, E84
ESC_FA9058AA	SAMEA92136418	5311641	234	243019	4961	Spain	2009	Avian	B	B3	Cl2	Cl2.3	O25b:H4	fimH22		gyrA A83, N87	parC S80, E84
ESC_FA9063AA	SAMEA92132668	5417238	130	234897	5090	Spain	2010	Avian	B	B3	Cl2	Cl2.3	O25b:H4	fimH22	CTX-M-9	gyrA A83, N87	parC S80, E84
ESC_FA9072AA	SAMEA92125918	5415181	178	207620	5089	Spain	2008	Avian	B	B3	Cl2	Cl2.3	O25b:H4	fimH22	CTX-M-9	gyrA A83, N87	parC S80, E84
ESC_FA9081AA	SAMEA92119168	5070196	83	293819	4725	Spain	2012	Avian	B	B3	Cl2	Cl2.1	O25b:H4	fimH22		gyrA L83, N87	parC S80, E84
ESC_FA9082AA	SAMEA92118418	4992071	47	558512	4626	Spain	2009	Avian	B	B3	Cl2	Cl2.1	O25b:H4	fimH22		gyrA S83, N87	parC S80, E84
ESC_FA9114AA	SAMEA92094418	5706892	277	111659	5394	Spain	2009	Avian	B	B3	Cl2	Cl2.3	O25b:H4	fimH22	CTX-M-9	gyrA A83, N87	parC S80, E84
ESC_FA9132AA	SAMEA92080918	5036318	119	112560	4674	Spain	2009	Avian	B	B3	Cl2	Cl2.1	O25b:H4	fimH22		gyrA S83, N87	parC S80, E84
ESC_FA9133AA	SAMEA92080168	5107372	309	99045	4732	Spain	2009	Avian	B	B3	Cl2	Cl2.1	O25b:H4	fimH22		gyrA S83, N87	parC S80, E84
ESC_FA9137AA	SAMEA92077168	5106396	149	169151	4733	Spain	2010	Avian	B	B3	Cl2	Cl2.1	O25b:H4	fimH22		gyrA L83, N87	parC S80, E84
ESC_FA9143AA	SAMEA92072668	5406447	146	181730	5089	Spain	2009	Avian	B	B3	Cl2	Cl2.3	O25b:H4	fimH22	CTX-M-9	gyrA A83, N87	parC S80, E84
ESC_GA3026AA	SAMN06645781	5561920	121	191062	5260	USA	2014	Avian	B	B3	Cl2	Cl2.3	O25b:H4	fimH22		gyrA S83, N87	parC S80, E84
ESC_GA6791AA	SAMN06645873	5260166	71	276246	4898	USA	2015	Avian	B	B5	Cl2	Cl2.4	O25b:H4	fimH22		gyrA S83, N87	parC S80, E84
ESC_HA0015AA	SAMEA104205967	5376645	94	276255	5074	Germany	2016	Avian	B	B3	Cl2	Cl2.2	O25b:H4	fimH22		gyrA L83, N87	parC S80, E84
ESC_HA0017AA	SAMEA104205965	5294631	71	365023	4957	Germany	2016	Avian	B	B3	Cl2	Cl2.2	O25b:H4	fimH22		gyrA L83, N87	parC S80, E84
ESC_HA0071AA	SAMEA104205911	5224857	101	364909	4890	Germany	2016	Avian	B	B3	Cl2	Cl2.1	O25b:H4	fimH22		gyrA L83, N87	parC S80, E84
ESC_HA7251AA	ESC_HA7251AA	5315422	100	191062	4978	USA	2017	Avian	B	B5	Cl2	Cl2.4	O25b:H4	fimH22		gyrA S83, N87	parC S80, E84
ESC_HA7630AA	SAMEA4058434	5248336	125	249351	4911	Denmark	2015	Avian	B	B3	Cl2	Cl2.2	O25b:H4	fimH22		gyrA S83, N87	parC S80, E84
ESC_HA7663AA	SAMEA4058401	5333329	98	460900	4961	Denmark	2015	Avian	B	B3	Cl2	Cl2.2	O25b:H4	fimH22		gyrA S83, N87	parC S80, E84
ESC_IA0869AA	SAMN08167533	5373851	78	294984	5032	USA	2017	Avian	B	B3	Cl2	Cl2.2	O25b:H4	fimH22		gyrA S83, N87	parC S80, E84
ESC_IA2817AA	SAMN08397033	5331464	147	165195	5047	USA	2007	Dolphin	C	C2	Cl3	Cl3.2	O25b:H4	fimH30		gyrA L83, N87	parC I80, V84
ESC_IA2820AA	SAMN08395877	4989803	52	341085	4650	USA	2006	Avian	B	B3	Cl2	Cl2.1	O25b:H4	fimH22		gyrA S83, N87	parC S80, E84
ESC_IA3889AA	SAMN07709787	5174082	109	191062	4850	USA	2017	Canine	C	C2	Cl3	Cl3.2	O25b:H4	fimH30	CTX-M-15	gyrA L83, N87	parC I80, V84
ESC_IA4037AA	SAMEA104407591	5506919	183	92963	5124	Germany	2012	Avian	B	B3	Cl2	Cl2.3	O25b:H4	fimH22		gyrA L83, N87	parC S80, E84
ESC_IA4046AA	SAMEA104407582	5505253	250	60186	5177	Germany	2012	Avian	B	B3	Cl2	Cl2.3	O25b:H4	fimH22		gyrA L83, N87	parC S80, E84
ESC_IA4090AA	SAMEA104407538	5133512	108	353089	4765	Germany	2012	Avian	B	B3	Cl2	Cl2.1	O25b:H4	fimH22		gyrA L83, N87	parC S80, E84
ESC_IA4093AA	SAMEA104407535	5378940	109	364370	5052	Germany	2012	Avian	B	B3	Cl2	Cl2.1	O25b:H4	fimH22		gyrA L83, N87	parC S80, E84
ESC_IA4095AA	SAMEA104407533	5133961	106	277592	4765	Germany	2012	Avian	B	B3	Cl2	Cl2.1	O25b:H4	fimH22		gyrA L83, N87	parC S80, E84

ESC_IA4127AA	SAMEA104407501	5256027	97	479242	4863	Germany	2012	Avian	B	B3	Cl2	Cl2.2	O25b:H4	fimH22		gyrA L83, N87	parC S80, E84
ESC_IA4128AA	SAMEA104407500	5282540	84	276315	4918	Germany	2012	Avian	B	B3	Cl2	Cl2.2	O25b:H4	fimH22		gyrA L83, N87	parC S80, E84
ESC_IA4131AA	SAMEA104407497	5133151	102	353089	4765	Germany	2012	Avian	B	B3	Cl2	Cl2.1	O25b:H4	fimH22		gyrA L83, N87	parC S80, E84
ESC_IA6627AA	SAMN08902758	5125730	150	120318	4799	USA	1982	Avian	B	B2	Cl2	Cl2.1	O25b:H4	fimH22		gyrA S83, N87	parC S80, E84
ESC_IA6645AA	SAMN08902743	5210633	168	109479	4906	USA	1983	Avian	B	B2	Cl2	Cl2.1	O25b:H4	fimH22		gyrA S83, N87	parC S80, E84
ESC_IA7281AA	SAMN08978091	5251659	56	292830	4946	USA	2000	Avian	B	B3	Cl2	Cl2.1	O25b:H4	fimH22		gyrA S83, N87	parC S80, E84
ESC_JA5749AA	SAMN09580015	5119139	91	272814	4807	Australia	2010	Avian	B	B3	Cl2	Cl2.1	O25b:H4	fimH22		gyrA S83, N87	parC S80, E84
ESC_JA7896AA	SAMN09729030	5019473	48	387398	4661	USA	2018	Canine	B	B5	Cl3	Cl3.1	O25b:H4	fimH22		gyrA S83, N87	parC S80, E84
ESC_JA8469AA	ERP109804	5209525	59	533878	4855	Denmark	2014	Avian	B	B3	Cl2	Cl2.2	O25b:H4	fimH22		gyrA L83, N87	parC S80, E84
ESC_JA8470AA	ERP109804	5246709	127	249351	4914	Denmark	2015	Avian	B	B3	Cl2	Cl2.2	O25b:H4	fimH22		gyrA S83, N87	parC S80, E84
ESC_JA8471AA	ERP109804	5332330	100	460900	4961	Denmark	2015	Avian	B	B3	Cl2	Cl2.2	O25b:H4	fimH22		gyrA S83, N87	parC S80, E84
ESC_KA0096AA	SAMN09954414	5197619	273	35213	4873	USA	2001	Avian	B	B3	Cl2	Cl2.1	O25b:H4	fimH22		gyrA S83, N87	parC S80, E84
ESC_KA0130AA	SAMN09951528	5420462	141	169427	5165	USA	2018	Canine	C	C2	Cl3	Cl3.3	O25b:H4	fimH30	CTX-M-15	gyrA L83, N87	parC I80, V84
ESC_KA1862AA	SAMN10095960	5214114	144	190607	4934	USA	2017	Avian	B	B3	Cl2	Cl2.1	O25b:H4	fimH22-like		gyrA S83, N87	parC S80, E84
ESC_KA3236AA	SAMN10235280	5182269	135	169004	4867	USA	2018	Canine	C	C1	Cl3	Cl3.2	O25b:H4	fimH30		gyrA L83, N87	parC I80, V84
ESC_KA3718AA	SAMN10221499	5069748	88	364910	4685	USA	2017	Avian	B	B3	Cl2	Cl2.2	O25b:H4	fimH22		gyrA S83, N87	parC S80, E84
ESC_KA3808AA	SAMN10221227	5625148	261	210569	5317	USA	2017	Avian	B	B3	Cl2	Cl2.5	O25b:H4	fimH22		gyrA S83, N87	parC S80, E84
ESC_KA3838AA	SAMN10221650	5218357	146	158951	4912	USA	2017	Avian	B	B3	Cl2	Cl2.4	O25b:H4	fimH22		gyrA S83, N87	parC S80, E84
ESC_KA3859AA	SAMN10221193	5496651	104	229887	5191	USA	2017	Avian	B	B3	Cl2	Cl2.5	O25b:H4	fimH22		gyrA S83, N87	parC S80, E84
ESC_KA3868AA	SAMN10221293	5261664	60	401109	4911	USA	2017		B	B3	Cl2	Cl2.3	O25b:H4	fimH22		gyrA S83, N87	parC S80, E84
ESC_KA3909AA	SAMN10221659	5262326	195	166548	4941	USA	2017	Avian	B	B5	Cl2	Cl2.1	O25b:H4	fimH22		gyrA S83, N87	parC S80, E84
ESC_KA3977AA	SAMN10221165	5214513	100	185436	4889	USA	2017	Avian	B	B3	Cl2	Cl2.4	O25b:H4	fimH22		gyrA S83, N87	parC S80, E84
ESC_KA3983AA	SAMN10221324	5244240	133	191626	4936	USA	2017	Avian	B	B3	Cl2	Cl2.4	O25b:H4	fimH22		gyrA S83, N87	parC S80, E84
ESC_KA4015AA	SAMN10221075	5211751	73	339934	4885	USA	2017	Avian	B	B5	Cl2	Cl2.4	O25b:H4	fimH22		gyrA S83, N87	parC S80, E84
ESC_KA4076AA	SAMN10221515	5261568	155	352886	4876	USA	2017	Avian	B	B3	Cl2	Cl2.1	O25b:H4	fimH22		gyrA S83, N87	parC S80, E84
ESC_KA4140AA	SAMN10221562	5450763	703	197593	5053	USA	2017	Avian	B	B5	Cl2	Cl2.4	O25b:H4	fimH22		gyrA S83, N87	parC S80, E84
ESC_KA4205AA	SAMN10221001	5292087	173	188050	5036	USA	2017	Avian	B	B3	Cl2	Cl2.1	O25b:H4	fimH22		gyrA S83, N87	parC S80, E84
ESC_KA4213AA	SAMN10221059	5292683	121	263792	4954	USA	2017	Avian	B	B5	Cl2	Cl2.1	O25b:H4	fimH22		gyrA S83, N87	parC S80, E84
ESC_KA4242AA	SAMN10221405	5247756	106	170661	4939	USA	2017	Avian	B	B3	Cl2	Cl2.4	O25b:H4	fimH22		gyrA S83, N87	parC S80, E84
ESC_KA4253AA	SAMN10221520	5301586	138	167392	4987	USA	2017	Avian	B	B3	Cl2	Cl2.4	O25b:H4	fimH22		gyrA S83, N87	parC S80, E84
ESC_KA4309AA	SAMN10221234	5238987	102	293968	4911	USA	2017	Avian	B	B5	Cl2	Cl2.1	O25b:H4	fimH22		gyrA S83, N87	parC S80, E84
ESC_KA4331AA	SAMN10221576	5068724	118	219772	4720	USA	2017	Avian	B	B3	Cl2	Cl2.1	O25b:H4	fimH22		gyrA S83, N87	parC S80, E84
ESC_KA4334AA	SAMN10221569	5210750	92	191062	4925	USA	2017	Avian	B	B2	Cl2	Cl2.1	O25b:H4	fimH22		gyrA S83, N87	parC S80, E84
ESC_KA4355AA	SAMN10221525	5484094	162	229882	5164	USA	2017	Avian	B	B3	Cl2	Cl2.5	O25b:H4	fimH22		gyrA S83, N87	parC S80, E84
ESC_KA4595AA	SAMN10316109	5027098	50	341558	4703	USA	1990	Avian	B	B3	Cl2	Cl2.1	O25b:H4	fimH22		gyrA S83, N87	parC S80, E84
ESC_KA4627AA	SAMN10315919	5384867	81	204493	5085	USA	1990	Avian	B	B3	Cl2	Cl2.4	O25b:H4	fimH22		gyrA S83, N87	parC S80, E84
ESC_KA5207AA	SAMN10396957	5223873	80	229879	4948	USA	2018	Canine	C	C1	Cl3	Cl3.2	O25b:H4	fimH30		gyrA L83, N87	parC I80, V84
ESC_KA5788AA	SAMN10442545	5289574	234	145139	4998	USA	2018	Canine	A	A1	Cl1	Cl1.1	O16:H5	fimH41		gyrA L83, N87	parC S80, E84
ESC_KA8008AA	SAMEA104483611	5422159	157	155982	5066	Germany	2012	Wild avian	B	B3	Cl2	Cl2.3	O25b:H4	fimH22		gyrA L83, N87	parC S80, E84
ESC_KA9030AA	SAMN10686130	5146656	274	59802	4870	Canada	2018	Canine	C	C1	Cl3	Cl3.2	O25b:H4	fimH30		gyrA L83, N87	parC I80, V84
ESC_KA9540AA	SAMN10751888	5206501	200	85694	4854	USA	2018	Canine	B	B3	Cl2	Cl2.1	O25b:H4	fimH22		gyrA S83, N87	parC S80, E84
ESC_LA1685AA	SAMN10883272	5406652	246	81431	5143	USA	2018	Canine	C	C2	Cl3	Cl3.3	O25b:H4	fimH30	CTX-M-15	gyrA L83, N87	parC I80, V84
ESC_LA9643AA	SAMN10478623	5118349	106	166727	4801	USA	2018	Canine	C	C1	Cl3	Cl3.2	O25b:H4	fimH30		gyrA L83, N87	parC I80, V84
ESC_MA1055AA	SAMN11127471	5060601	80	187059	4740	USA	2018	Canine	B	B5	Cl3	Cl3.1	O25b:H4	fimH22		gyrA S83, N87	parC S80, E84
ESC_MA1299AA	SAMN11129378	5247213	356	94892	4840	USA	2019	Avian	B	B5	Cl3	Cl3.1	O25b:H4	fimH22		gyrA S83, N87	parC S80, E84
ESC_MA1803AA	SAMN11249687	4930163	28	391399	4548	USA	2018	Canine	B	B2	Cl2	Cl2.1	O62:H4	fimH22-like		gyrA S83, N87	parC S80, E84
ESC_MA1950AA	SAMN11288804	5064762	58	276256	4714	USA	2018	Canine	B	B5	Cl3	Cl3.1	O25b:H4	fimH22-like		gyrA S83, N87	parC S80, E84
ESC_MA1951AA	SAMN11288805	4710129	29	713497	4344	USA	2018	Canine	B	B1	Cl3	Cl3.1	O25b:H4	fimH22		gyrA S83, N87	parC S80, E84
ESC_MA2890AA	SAMN11352643	5378033	162	133976	5076	USA	2018	Canine	C	C2	Cl3	Cl3.2	O25b:H4	fimH30		gyrA L83, N87	parC I80, V84

ESC_MA3038AA	SAMN11371666	5020947	131	124364	4622	USA	2018	Canine	B	B5	Cl3	Cl3.1	O25b:H4	fimH22-like		gyrA S83, N87	parC S80, E84
ESC_MA3811AA	SAMN11417756	5115323	146	147632	4810	USA	2018	Canine	C	C1	Cl3	Cl3.2	O25b:H4	fimH30	CTX-M-14	gyrA L83, N87	parC I80, V84
ESC_MA5127AA	SAMN11372927	5416552	401	89860	5037	USA	2018	Avian	B	B3	Cl2	Cl2.3	O25b:H4	fimH22		gyrA S83, N87	parC S80, E84
ESC_MA5807AA	SAMN11634809	5168974	108	188452	4779	USA	2019	Avian	B	B5	Cl2	Cl2.1	O25b:H4	fimH22		gyrA S83, N87	parC S80, E84
ESC_MA6941AA	SAMN11842196	5009305	94	191062	4692	USA	2018	Canine	C	C1	Cl3	Cl3.2	O25b:H4	fimH30		gyrA L83, N87	parC I80, V84
ESC_MA7197AA	SAMEA5597538	5152681	118	163855	4823	Netherlands	2015	Canine	C	C1	Cl3	Cl3.2	O25b:H4	fimH30	CTX-M-27	gyrA L83, N87	parC I80, V84
ESC_MA7198AA	SAMEA5597537	5136555	282	47800	4791	Netherlands	2015	Canine	C	C1	Cl3	Cl3.2	O25b:H4	fimH30	CTX-M-27	gyrA L83, N87	parC I80, V84
ESC_MA8166AA	SAMEA5611234	5255639	98	225174	4951	Kenya	2016	Avian	B	B4	Cl3	Cl3.1	O25b:H4	fimH30		gyrA S83, N87	parC S80, E84
ESC_NA0121AA	SAMN11958802	5203309	118	140119	4898	USA	2019	Avian	B	B3	Cl2	Cl2.4	O25b:H4	fimH22		gyrA S83, N87	parC S80, E84
ESC_NA1840AA	ESC_NA1840AA	5504985	684	28014	5136	Unknown	2018	Avian	B	B3	Cl2	Cl2.5	O25b:H4	fimH22		gyrA S83, N87	parC S80, E84
ESC_NA1842AA	ESC_NA1842AA	5250039	507	28204	4921	Unknown	2018	Avian	B	B3	Cl2	Cl2.4	O25b:H4	fimH22		gyrA S83, N87	parC S80, E84
ESC_NA1845AA	ESC_NA1845AA	5250790	692	21673	4863	Unknown	2018	Avian	B	B3	Cl2	Cl2.4	O25b:H4	fimH22		gyrA S83, N87	parC S80, E84
ESC_NA1870AA	ESC_NA1870AA	5223662	367	39736	4884	Unknown	2018	Avian	B	B3	Cl2	Cl2.4	O25b:H4	fimH22		gyrA S83, N87	parC S80, E84
ESC_NA1885AA	ESC_NA1885AA	5254273	367	37842	4921	Unknown	2018	Avian	B	B3	Cl2	Cl2.4	O25b:H4	fimH22		gyrA S83, N87	parC S80, E84
ESC_NA1929AA	ESC_NA1929AA	5343363	434	29707	4989	Unknown	2018	Avian	B	B3	Cl2	Cl2.5		fimH22		gyrA S83, N87	parC S80, E84
ESC_NA1931AA	ESC_NA1931AA	5397410	503	37617	5016	Unknown	2018	Avian	B	B3	Cl2	Cl2.5	O25b:H4	fimH22		gyrA S83, N87	parC S80, E84
ESC_NA1942AA	ESC_NA1942AA	5399607	512	44917	5019	Unknown	2018	Avian	B	B3	Cl2	Cl2.5	O25b:H4	fimH22		gyrA S83, N87	parC S80, E84
ESC_NA1948AA	ESC_NA1948AA	5377057	385	33148	5050	Unknown	2018	Avian	B	B3	Cl2	Cl2.5	O25b:H4	fimH22		gyrA S83, N87	parC S80, E84
ESC_NA1955AA	ESC_NA1955AA	5358966	363	37486	5018	Unknown	2018	Avian	B	B3	Cl2	Cl2.5	O25b:H4	fimH22		gyrA S83, N87	parC S80, E84
ESC_NA1957AA	ESC_NA1957AA	5423812	562	38867	5037	Unknown	2018	Avian	B	B3	Cl2	Cl2.5	O25b:H4	fimH22		gyrA S83, N87	parC S80, E84
ESC_NA1964AA	ESC_NA1964AA	5470392	430	35025	5123	Unknown	2018	Avian	B	B3	Cl2	Cl2.5	O25b:H4	fimH22		gyrA S83, N87	parC S80, E84
ESC_NA1967AA	ESC_NA1967AA	5385035	331	44634	5028	Unknown	2018	Avian	B	B3	Cl2	Cl2.5	O25b:H4	fimH22		gyrA S83, N87	parC S80, E84
ESC_NA1979AA	ESC_NA1979AA	5210685	375	34504	4863	Unknown	2018	Avian	B	B3	Cl2	Cl2.4	O25b:H4	fimH22		gyrA S83, N87	parC S80, E84
ESC_NA1981AA	ESC_NA1981AA	5212872	350	44838	4875	Unknown	2018	Avian	B	B3	Cl2	Cl2.4	O25b:H4	fimH22		gyrA S83, N87	parC S80, E84
ESC_NA1990AA	ESC_NA1990AA	5442514	493	27099	5111	Unknown	2018	Avian	B	B3	Cl2	Cl2.5	O25b:H4	fimH22		gyrA S83, N87	parC S80, E84
ESC_NA1994AA	ESC_NA1994AA	5485082	366	36759	5172	Unknown	2018	Avian	B	B3	Cl2	Cl2.5	O25b:H4	fimH22		gyrA S83, N87	parC S80, E84
ESC_NA1996AA	ESC_NA1996AA	5122312	674	17142	4769	Unknown	2018	Avian	B	B3	Cl2	Cl2.4	O25b:H4	fimH22		gyrA S83, N87	parC S80, E84
ESC_NA2000AA	ESC_NA2000AA	5236666	255	53813	4910	Unknown	2018	Avian	B	B3	Cl2	Cl2.4	O25b:H4	fimH22		gyrA S83, N87	parC S80, E84
ESC_NA2002AA	ESC_NA2002AA	5238843	277	57694	4896	Unknown	2018	Avian	B	B3	Cl2	Cl2.4	O25b:H4	fimH22		gyrA S83, N87	parC S80, E84
ESC_NA2010AA	ESC_NA2010AA	5484790	381	35137	5167	Unknown	2018	Avian	B	B3	Cl2	Cl2.5	O25b:H4	fimH22		gyrA S83, N87	parC S80, E84
ESC_NA2029AA	ESC_NA2029AA	5449434	456	29248	5134	Unknown	2018	Avian	B	B3	Cl2	Cl2.5		fimH22		gyrA S83, N87	parC S80, E84
ESC_NA2031AA	ESC_NA2031AA	5238377	362	36452	4922	Unknown	2018	Avian	B	B3	Cl2	Cl2.4	O25b:H4	fimH22		gyrA S83, N87	parC S80, E84
ESC_NA2091AA	ESC_NA2091AA	5370368	217	80124	5070	Unknown	2018	Avian	B	B3	Cl2	Cl2.4	O25b:H4	fimH22		gyrA S83, N87	parC S80, E84
ESC_NA2093AA	ESC_NA2093AA	5458221	576	24350	5118	Unknown	2018	Avian	B	B3	Cl2	Cl2.5	O25b:H4	fimH22		gyrA S83, N87	parC S80, E84
ESC_NA2105AA	ESC_NA2105AA	5708014	329	75129	5441	Unknown	2018	Avian	B	B5	Cl2	Cl2.3	O25b:H4	fimH22		gyrA S83, N87	parC S80, E84
ESC_NA2108AA	ESC_NA2108AA	5334557	373	36714	5045	Unknown	2018	Avian	B	B3	Cl2	Cl2.4	O25b:H4	fimH22		gyrA S83, N87	parC S80, E84
ESC_NA2118AA	ESC_NA2118AA	5208311	432	33115	4873	Unknown	2018	Avian	B	B3	Cl2	Cl2.4	O25b:H4	fimH22		gyrA S83, N87	parC S80, E84
ESC_NA2138AA	ESC_NA2138AA	5487281	276	60474	5188	Unknown	2018	Avian	B	B3	Cl2	Cl2.5	O25b:H4	fimH22		gyrA S83, N87	parC S80, E84
ESC_NA2144AA	ESC_NA2144AA	5357443	353	38508	4956	Unknown	2018	Avian	B	B3	Cl2	Cl2.1	O25b:H4	fimH22		gyrA S83, N87	parC S80, E84
ESC_NA2146AA	ESC_NA2146AA	5243206	255	60087	4931	Unknown	2018	Avian	B	B3	Cl2	Cl2.4	O25b:H4	fimH22		gyrA S83, N87	parC S80, E84
ESC_NA2172AA	ESC_NA2172AA	5276977	413	27572	4992	Unknown	2018	Avian	B	B3	Cl2	Cl2.4	O25b:H4	fimH22		gyrA S83, N87	parC S80, E84
ESC_NA2193AA	ESC_NA2193AA	5226395	366	35167	4908	Unknown	2018	Avian	B	B3	Cl2	Cl2.4	O25b:H4	fimH22		gyrA S83, N87	parC S80, E84
ESC_NA2194AA	ESC_NA2194AA	5554630	330	45558	5258	Unknown	2018	Avian	B	B3	Cl2	Cl2.5	O25b:H4	fimH22		gyrA S83, N87	parC S80, E84
ESC_NA2199AA	ESC_NA2199AA	5463595	537	24097	5150	Unknown	2018	Avian	B	B3	Cl2	Cl2.5		fimH22		gyrA S83, N87	parC S80, E84
ESC_NA2201AA	ESC_NA2201AA	5483826	457	30176	5168	Unknown	2018	Avian	B	B3	Cl2	Cl2.5	O25b:H4	fimH22		gyrA S83, N87	parC S80, E84
ESC_NA2205AA	ESC_NA2205AA	5599842	432	32835	5298	Unknown	2018	Avian	B	B3	Cl2	Cl2.5	O25b:H4	fimH22		gyrA S83, N87	parC S80, E84
ESC_NA2209AA	ESC_NA2209AA	5396545	370	48223	5032	Unknown	2018	Avian	B	B3	Cl2	Cl2.3	O25b:H4	fimH22		gyrA L83, N87	parC S80, E84
ESC_NA2215AA	ESC_NA2215AA	5308559	364	38500	4961	Unknown	2018	Avian	B	B3	Cl2	Cl2.3	O25b:H4	fimH22		gyrA L83, N87	parC S80, E84
ESC_NA2224AA	ESC_NA2224AA	5486884	408	35882	5181	Unknown	2018	Avian	B	B3	Cl2	Cl2.5	O25b:H4	fimH22		gyrA S83, N87	parC S80, E84
ESC_NA2276AA	ESC_NA2276AA	5267744	294	45128	4911	Unknown	2018	Avian	B	B5	Cl2	Cl2.1	O25b:H4	fimH22		gyrA S83, N87	parC S80, E84

ESC_NA2288AA	ESC_NA2288AA	5437417	376	36455	5119	Unknown	2018	Avian	B	B3	Cl2	Cl2.5	O25b:H4	fimH22		gyrA S83, N87	parC S80, E84
ESC_NA2289AA	ESC_NA2289AA	5457403	433	30045	5133	Unknown	2018	Avian	B	B3	Cl2	Cl2.5	O25b:H4	fimH22		gyrA S83, N87	parC S80, E84
ESC_NA2325AA	ESC_NA2325AA	5468320	417	33864	5123	Unknown	2018	Avian	B	B5	Cl2	Cl2.4	O25b:H4	fimH22		gyrA S83, N87	parC S80, E84
ESC_NA2330AA	ESC_NA2330AA	5462075	259	59438	5171	Unknown	2018	Avian	B	B3	Cl2	Cl2.5	O25b:H4	fimH22		gyrA S83, N87	parC S80, E84
ESC_NA2391AA	ESC_NA2391AA	5096681	176	102082	4731	Unknown	2018	Avian	B	B3	Cl2	Cl2.1	O25b:H4	fimH22		gyrA S83, N87	parC S80, E84
ESC_NA2394AA	ESC_NA2394AA	5151992	259	60619	4779	Unknown	2018	Avian	B	B3	Cl2	Cl2.1	O25b:H4	fimH22		gyrA S83, N87	parC S80, E84
ESC_NA2396AA	ESC_NA2396AA	5396213	267	45488	5045	Unknown	2018	Avian	B	B3	Cl2	Cl2.3	O25b:H4	fimH22		gyrA S83, N87	parC S80, E84
ESC_NA2523AA	ESC_NA2523AA	5270855	211	115697	4962	Unknown	2018	Avian	B	B3	Cl2	Cl2.4	O25b:H4	fimH22		gyrA S83, N87	parC S80, E84
ESC_NA3867AA	SAMN12175445	5130667	91	222640	4778	USA	2019	Canine	B	B5	Cl3	Cl3.1	O25b:H4	fimH22		gyrA S83, N87	parC S80, E84
ESC_NA6079AA	SAMN12359541	5311839	92	349172	4971	USA	2018	Avian	B	B3	Cl2	Cl2.2	O25b:H4	fimH22		gyrA S83, N87	parC S80, E84
ESC_NA6103AA	SAMN12359549	5025210	72	382706	4695	USA	2018	Avian	B	B3	Cl2	Cl2.1	O25b:H4	fimH22		gyrA S83, N87	parC S80, E84
ESC_NA6619AA	SAMN12438581	5381016	83	301763	5065	USA	2005	Avian	B	B5	Cl2	Cl2.3	O25b:H4	fimH22		gyrA L83, N87	parC S80, E84
ESC_NA6650AA	SAMN12438610	4981749	87	251609	4651	USA	2007	Canine	C	C1	Cl3	Cl3.2	O25b:H4	fimH30		gyrA L83, N87	parC I80, V84
ESC_NA6652AA	SAMN12438612	5315499	79	340916	4982	USA	2007	Wild avian	B	B3	Cl2	Cl2.3	O25b:H4	fimH22		gyrA S83, N87	parC S80, E84
ESC_NA6729AA	SAMN12438659	5301482	147	192080	5008	USA	2012	Avian	B	B3	Cl2	Cl2.1	O25b:H4	fimH22		gyrA S83, N87	parC S80, E84
ESC_NA6741AA	SAMN12438667	5335725	57	312509	4996	USA	2013	Wild avian	B	B3	Cl2	Cl2.2	O25b:H4	fimH22		gyrA S83, N87	parC S80, E84
ESC_NA6788AA	SAMN12438599	4984805	41	379011	4650	USA	2006	Avian	B	B3	Cl2	Cl2.1	O25b:H4	fimH22		gyrA S83, N87	parC S80, E84
ESC_NA7890AA	SAMN12359664	5249114	92	386421	4893	USA	2018	Avian	B	B3	Cl2	Cl2.1	O25b:H4	fimH22		gyrA S83, N87	parC S80, E84
ESC_NA7904AA	SAMN12359654	5475922	166	263808	5130	USA	2018	Avian	B	B5	Cl2	Cl2.1	O25b:H4	fimH22		gyrA S83, N87	parC S80, E84
ESC_NA7916AA	SAMN12359895	5492387	177	229771	5208	USA	2018	Avian	B	B3	Cl2	Cl2.5	O25b:H4	fimH22		gyrA S83, N87	parC S80, E84
ESC_NA7982AA	SAMN12359705	5612160	217	168876	5347	USA	2018	Avian	B	B3	Cl2	Cl2.5	O25b:H4	fimH22		gyrA S83, N87	parC S80, E84
ESC_NA8019AA	SAMN12359616	5511009	194	124318	5215	USA	2018	Avian	B	B3	Cl2	Cl2.5	O25b:H4	fimH22		gyrA S83, N87	parC S80, E84
ESC_OA0125AA	SAMN12715618	5098601	99	203049	4724	USA	2019	Canine	A	A1	Cl1	Cl1.1	O16:H5	fimH41	CTX-M-27	gyrA L83, N87	parC S80, E84
ESC_OA0213AA	SAMN12722857	4976959	107	155603	4674	USA	2019	Canine	C	C1	Cl3	Cl3.2	O25b:H4	fimH30		gyrA L83, N87	parC I80, V84
ESC_OA2927AA	SAMN12822643	5166836	161	91132	4824	USA	2019	Avian	B	B3	Cl2	Cl2.4	O25b:H4	fimH22		gyrA S83, N87	parC S80, E84
F283	SRR3143607	5084694	116	159071	4778	USA	2012	Human	C	C1	Cl3	Cl3.2	O25b:H4	fimH30	CTX-M-27	gyrA L83, N87	parC I80, V84
FG10	SAMEA7758300	5085668	80	222205	4763	La Reunion	2013	Human	C	C1	Cl3	Cl3.2	O25b:H4	fimH30	CTX-M-27	gyrA L83, N87	parC I80, V84
FG11	SAMEA7758301	5474614	96	178427	5268	La Reunion	2013	Human	C	C2	Cl3	Cl3.3	O25b:H4	fimH30	CTX-M-15	gyrA L83, N87	parC I80, V84
FG12	SAMEA7758302	5109264	72	195295	4833	La Reunion	2013	Human	C	C2	Cl3	Cl3.2	O25b:H4	fimH30	CTX-M-15	gyrA L83, N87	parC I80, V84
FG47	SAMEA7758303	5216545	127	182116	4923	La Reunion	2013	Human	A	A1	Cl1	Cl1.1	O16:H5	fimH41	CTX-M-14	gyrA L83, N87	parC S80, E84
FG49	SAMEA7758304	5281391	93	175757	5052	La Reunion	2013	Human	C	C1	Cl3	Cl3.2	O25b:H4	fimH30	CTX-M-27	gyrA L83, N87	parC I80, V84
FG5	SAMEA7758305	5393253	97	213617	5180	La Reunion	2013	Human	C	C2	Cl3	Cl3.3	O25b:H4	fimH30	CTX-M-15	gyrA L83, N87	parC I80, V84
FG53	SAMEA7758306	5311199	78	229843	5068	La Reunion	2013	Human	C	C2	Cl3	Cl3.2	O25b:H4	fimH30	CTX-M-15	gyrA L83, N87	parC I80, V84
G132	SRR933363	5299521	168	204333	5030	USA	2010	Human	B	B4	Cl3	Cl3.1	O25b:H4	fimH22-like		gyrA S83, N87	parC S80, E84
G199	SRR933367	5213568	107	168312	4938	USA	2010	Human	B	B4	Cl3	Cl3.1	O25b:H4	fimH30		gyrA S83, N87	parC S80, E84
G216	SRR933371	5291751	157	186345	4988	USA	2010	Human	I	I3	Cl3	Cl3.1	O25b:H4	fimH22		gyrA S83, N87	parC S80, E84
G749	SRS4603360	5542186	44	499865	5285	USA	2011	Human	B	B3	Cl2	Cl2.1	O25b:H4	fimH22		gyrA S83, N87	parC S80, E84
GHSR24	SAMEA7758307	5226348	75	225743	4988	La Reunion	2013	Human	C	C2	Cl3	Cl3.3	O25b:H4	fimH30	CTX-M-15	gyrA L83, N87	parC I80, V84
GHSR33	SAMEA7758308	5357648	87	222117	5130	La Reunion	2013	Human	C	C2	Cl3	Cl3.3	O25b:H4	fimH30	CTX-M-15	gyrA L83, N87	parC I80, V84
GHSR36	SAMEA7758309	5377586	87	159972	5139	La Reunion	2014	Human	C	C2	Cl3	Cl3.3	O25b:H4	fimH30	CTX-M-15	gyrA L83, N87	parC I80, V84
GHSR37	SAMEA7758310	5324052	99	170065	5096	La Reunion	2013	Human	C	C2	Cl3	Cl3.3	O25b:H4	fimH30	CTX-M-15	gyrA L83, N87	parC I80, V84
H016	SRR933377	5089095	78	165059	4793	USA	2010	Human	C	C1	Cl3	Cl3.2	O25b:H4	fimH30		gyrA L83, N87	parC I80, V84
H061	SRR933379	5102798	102	284024	4796	USA	2011	Human	B	B4	Cl3	Cl3.1	O25b:H4	fimH22		gyrA S83, N87	parC S80, E84
H1088	SRS5403671	5157865	150	144048	4865	Spain	2001	Human	I	I4	Cl3	Cl3.2	O25b:H4	fimH30		gyrA S83, N87	parC S80, E84
H1447	SRS5403665	5040800	150	97675	4749	Spain	2004	Human	B	B5	Cl2	Cl2.1	O25b:H4	fimH22		gyrA L83, N87	parC S80, E84
H17	SRR933381	5314288	131	139752	5068	USA	1985	Human	I	I1	Cl3	Cl3.1	O25b:H4	fimH27		gyrA S83, N87	parC S80, E84
H2262	SRS5403669	5480217	186	171501	5172	Spain	2008	Human	B	B5	Cl2	Cl2.3	O25b:H4	fimH22	CTX-M-9	gyrA S83, N87	parC S80, E84
H3084B	SRS5403686	5022489	149	129384	4710	Spain	2010	Human	C	C1	Cl3	Cl3.2	O25b:H4	fimH30		gyrA L83, N87	parC I80, V84
HP47	SRR3143608	5271832	127	170885	4941	Czech Republic	2010	Avian	C	C2	Cl3	Cl3.2	O25b:H4	fimH30	CTX-M-15	gyrA L83, N87	parC I80, V84
HS115	SRR3143609	5096517	119	159792	4804	Serbia	2010	Avian	C	C1	Cl3	Cl3.2	O25b:H4	fimH30	CTX-M-15	gyrA L83, N87	parC I80, V84
HVM1147	ERR161318	5131204	93	190858	4811	Spain	2010	Human	B	B4	Cl3	Cl3.1	O25b:H4	fimH22		gyrA S83, N87	parC S80, E84
HVM1299	ERR161320	5292889	221	123915	5018	Spain	2010	Human	C	C2	Cl3	Cl3.3	O25b:H4	fimH30	CTX-M-15	gyrA L83, N87	parC I80, V84
HVM1997	ERR161322	5207466	235	142083	4886	Spain	2010	Human	C	C2	Cl3	Cl3.2	O25b:H4	fimH30	CTX-M-15	gyrA L83, N87	parC I80, V84
HVM2044	ERR161323	5208744	157	171471	4852	Spain	2010	Human	B	B1	Cl2	Cl2.1	O25b:H4	fimH22		gyrA L83, N87	parC S80, E84

HVM2289	ERR161325	5206852	157	123929	4858	Spain	2010	Human	B	B1	Cl2	Cl2.1	O25b:H4	fimH22		gyrA L83, N87	parC S80, E84
HVM3017	ERR161328	5277465	201	147586	4996	Spain	2010	Human	C	C2	Cl3	Cl3.3	O25b:H4	fimH30	CTX-M-15	gyrA L83, N87	parC I80, V84
HVM5	ERR161306	4972485	164	130373	4641	Spain	2010	Human	C	C1	Cl3	Cl3.2	O25b:H4	fimH30		gyrA L83, N87	parC I80, V84
HVM826	ERR161316	5096484	170	178532	4761	Spain	2010	Human	C	C1	Cl3	Cl3.2	O25b:H4	fimH30		gyrA L83, N87	parC I80, V84
HVR2496	ERR161326	5014198	196	96567	4666	Spain	2010	Human	C	C1	Cl3	Cl3.2	O25b:H4	fimH30		gyrA L83, N87	parC I80, V84
HVR83	ERR161311	5312443	197	159055	5034	Spain	2010	Human	C	C1	Cl3	Cl3.2	O25b:H4	fimH30		gyrA L83, N87	parC I80, V84
I34	SAMN12990271	5042652	47	303510	4687	France	2017	Canine	B	B5	Cl3	Cl3.1	O25b:H4	fimH298		gyrA S83, N87	parC S80, E84
IHIT25637	SAMN05257099	5690890	140	234455	5386	Germany	2010	Avian	B	B3			O25b:H4	fimH22		gyrA L83, N87	parC S80, E84
IR18E	ERR458470	5254399	204	147009	4963	India	2009	Human	C	C2	Cl3	Cl3.2	O25b:H4	fimH30	CTX-M-15	gyrA L83, N87	parC I80, V84
IR49	ERR458471	5321156	180	159067	4990	India	2009	Human	C	C2	Cl3	Cl3.2	O25b:H4	fimH30	CTX-M-15	gyrA L83, N87	parC I80, V84
IR65	ERR458472	5314775	176	133585	4992	India	2009	Human	C	C2	Cl3	Cl3.2	O25b:H4	fimH30	CTX-M-15	gyrA L83, N87	parC I80, V84
IR68	ERR458473	5330034	180	167452	5017	India	2009	Human	C	C2	Cl3	Cl3.2	O25b:H4	fimH30	CTX-M-15	gyrA L83, N87	parC I80, V84
J21	SRR3143611	5143831	101	190421	4838	China	2011	Human	I	I2	Cl3	Cl3.2	O25b:H4	fimH54		gyrA L83, N87	parC S80, E84
JJ1897	SRR933383	5230463	130	186345	4912	USA	2004	Human	I	I3	Cl3	Cl3.2	O25b:H4	fimH22		gyrA S83, N87	parC S80, E84
JJ1999	SRR933349	4974763	68	242563	4613	India	2007	Human	B	B3	Cl2	Cl2.1	O25b:H4	fimH22		gyrA S83, N87	parC S80, E84
JJ2038	SRR933411	5147708	106	165773	4837	USA	2007	Human	C	C2	Cl3	Cl3.2	O25b:H4	fimH30	CTX-M-15	gyrA L83, N87	parC I80, V84
JJ2055	SRR933339	5054462	180	137500	4647	USA	2007	Human	A	A1	Cl1	Cl1.1	O16:H5	fimH41	CTX-M-14	gyrA S83, N87	parC S80, E84
JJ2087	SRR933415	5092075	132	190921	4791	USA	2003	Human	B	B2	Cl2	Cl2.1	O25b:H4	fimH22-like		gyrA L83 G87	parC R80, E84
JJ2118	SRR933341	4986712	71	188593	4693	USA	2008	Human	C	C1	Cl3	Cl3.2	O25b:H4	fimH30		gyrA L83, N87	parC I80, V84
JJ2134	SRR933343	5034366	50	191973	4742	USA	2008	Human	C	C2	Cl3	Cl3.2	O25b:H4	fimH30		gyrA L83, N87	parC I80, V84
JJ2193	SRR933419	5114681	128	157538	4826	USA	2008	Human	C	C1	Cl3	Cl3.2	O25b:H4	fimH30		gyrA L83, N87	parC I80, V84
JJ2444	SRR933513	5081289	73	159648	4800	USA	2008	Human	C	C2	Cl3	Cl3.2	O25b:H4	fimH30	CTX-M-15	gyrA L83, N87	parC I80, V84
JJ2489	SRR933547	5076690	83	134597	4767	USA	2007	Human	C	C2	Cl3	Cl3.2	O25b:H4	fimH30	CTX-M-15	gyrA L83, N87	parC I80, V84
JJ2508	SRR933515	5151352	91	177965	4860	USA	2008	Human	C	C1	Cl3	Cl3.2	O25b:H4	fimH30		gyrA L83, N87	parC I80, V84
JJ2528	SRR933517	5328633	131	135849	5101	USA	2007	Human	C	C1	Cl3	Cl3.2	O25b:H4	fimH30	CTX-M-14	gyrA L83, N87	parC I80, V84
JJ2547	SRR933387	5142775	82	213193	4832	USA	2009	Human	C	C2	Cl3	Cl3.2	O25b:H17	fimH30	CTX-M-15	gyrA L83, N87	parC I80, V84
JJ2550	SRR933519	5074362	127	109047	4758	USA	2007	Human	C	C1	Cl3	Cl3.2	O25b:H4	fimH30		gyrA L83, N87	parC I80, V84
JJ2578	SRR933523	5152585	87	186345	4870	USA	2008	Human	C	C1	Cl3	Cl3.2	O25b:H4	fimH30		gyrA L83, N87	parC I80, V84
JJ2591	SRR933525	5065514	77	191752	4742	USA	2006	Human	A	A1	Cl1	Cl1.1	O16:H5	fimH41	CTX-M-15	gyrA L83, N87	parC S80, E84
JJ2608	SRR933527	4928389	87	158869	4602	USA	2008	Human	C	C1	Cl3	Cl3.2	O25b:H4	fimH30		gyrA L83, N87	parC I80, V84
JJ2643	SRR933421	5072929	95	185474	4772	USA	2008	Human	C	C2	Cl3	Cl3.2	O25b:H4	fimH35	CTX-M-15	gyrA L83, N87	parC I80, V84
JJ2657	SRR933529	5182742	86	186460	4882	USA	2009	Human	C	C2	Cl3	Cl3.2	O25b:H4	fimH30	CTX-M-15	gyrA L83, N87	parC I80, V84
JJ2668	SRR933531	5107555	101	162141	4847	USA	2009	Human	C	C2	Cl3	Cl3.2	O25b:H4	fimH30		gyrA L83, N87	parC I80, V84
JMI025	SRR933389	4971591	109	82933	4634	USA	2000	Human	B	B2	Cl3	Cl3.1	O25b:H4	fimH35		gyrA L83 G87	parC R80, E84
KCH38	DRR092882	5191637	87	240452	4928	Japan	2009	Human	C	C3	Cl3	Cl3.2	O25b:H4	fimH30	CTX-M-14	gyrA L83, N87	parC I80, V84
KFEC1	DRR092860	5558623	132	165682	5326	Japan	2003	Human	I	I3	Cl3	Cl3.2	O25b:H4	fimH22	CTX-M-2	gyrA S83, N87	parC S80, E84
KK58	DRR092879	5256918	138	173945	4992	Japan	2012	Human	C	C1	Cl3	Cl3.2	O25b:H4	fimH30	CTX-M-27	gyrA L83, N87	parC I80, V84
KK85	DRR092880	5011418	78	205332	4647	Japan	2012	Human	A	A1	Cl1	Cl1.1	O16:H5	fimH41	CTX-M-27	gyrA S83, N87	parC S80, E84
KN1604	SRR933537	5141513	92	172642	4853	Korea	2003	Human	C	C2	Cl3	Cl3.2	O25b:H4	fimH30	CTX-M-15	gyrA L83, N87	parC I80, V84
KN8	DRR092863	5200725	75	191865	4902	Japan	2010	Human	I	I2	Cl3	Cl3.2	O25b:H4	fimH54	CTX-M-14	gyrA L83, N87	parC S80, E84
KN93	DRR092881	4951810	76	270681	4571	Japan	2012	Human	A	A1	Cl1	Cl1.1	O16:H5	fimH41	CTX-M-14	gyrA L83, N87	parC I80, E84
KN95	DRR092890	5096923	100	209445	4811	Japan	2012	Human	C	C3	Cl3	Cl3.2	O25b:H4	fimH30	CTX-M-14	gyrA L83, N87	parC I80, V84
KO178B	SRR3143612	5052260	104	146809	4712	Czech Republic	2007	Wild avian	C	C1	Cl3	Cl3.2	O25b:H4	fimH30	CTX-M-27	gyrA L83, N87	parC I80, V84
KO198B	SRR3143613	5048305	81	190712	4718	Serbia	2007	Wild avian	C	C1	Cl3	Cl3.2	O25b:H4	fimH30	CTX-M-27	gyrA L83, N87	parC I80, V84
KP13	DRR092864	5191679	104	344663	4884	Japan	2010	Human	I	I2	Cl3	Cl3.2	O25b:H4	fimH54	CTX-M-14	gyrA L83, N87	parC S80, E84
KP67	DRR092876	4862351	50	229881	4476	Japan	2011	Human	A	A1	Cl1	Cl1.1	O16:H5	fimH41	CTX-M-15	gyrA S83, N87	parC S80, E84
KS127	DRR092883	5170194	63	345528	4853	Japan	2012	Human	B	B3	Cl2	Cl2.2	O25b:H4	fimH22	CTX-M-1	gyrA L83, N87	parC S80, E84
KS17	DRR092888	5188221	103	167601	4906	Japan	2010	Human	C	C3	Cl3	Cl3.2	O25b:H4	fimH30	CTX-M-14	gyrA L83, N87	parC I80, V84
KS26	DRR051015	5086513	106	112714	4757	Japan	2010	Human	C	C1	Cl3	Cl3.2	O25b:H4	fimH30	CTX-M-27	gyrA L83, N87	parC I80, V84
KSEC12	DRR092861	4924252	60	377622	4553	Japan	2004	Human	A	A1	Cl1	Cl1.1	O16:H5	fimH41	CTX-M-2	gyrA S83, N87	parC S80, E84
KSEC14	DRR092862	5285409	76	230102	5014	Japan	2004	Human	I	I3	Cl3	Cl3.2	O25b:H4	fimH22	CTX-M-2	gyrA S83, N87	parC S80, E84
KT10	DRR051024	4950951	108	129773	4630	Japan	2012	Human	C	C1	Cl3	Cl3.2	O25b:H4	fimH30	CTX-M-27	gyrA L83, N87	parC I80, V84

KT43	DRR092884	5042897	74	256897	4688	Japan	2012	Human	A	A1	Cl1	Cl1.1	O16:H5	fimH89	CTX-M-14	gyrA L83, N87	parC S80, E84
KUEC2	DRR092859	5271185	77	212537	4993	Japan	2001	Human	B	B4	Cl3	Cl3.1	O25b:H4	fimH22	CTX-M-2	gyrA S83, N87	parC S80, E84
KUN2030	DRS045503	5151683	109	229886	4857	Japan	2006	Human	C	C3	Cl3	Cl3.2	O25b:H4	fimH30	CTX-M-14	gyrA L83, N87	parC I80, V84
KUN4343	DRS045507	5138008	118	233359	4869	Japan	2008	Human	C	C3	Cl3	Cl3.2	O25b:H4	fimH30	CTX-M-14	gyrA L83, N87	parC I80, V84
KUN8724	DRR092867	5186617	54	344905	4906	Japan	2011	Human	I	I2	Cl3	Cl3.2	O25b:H4	fimH54	CTX-M-14	gyrA L83, N87	parC S80, E84
L01h	SAMEA7758321	5444162	194	173926	4866	France	2006	Human	C	C1	Cl3	Cl3.2	O25b:H4	fimH30		gyrA L83, N87	parC I80, V84
L02h	SAMEA7758322	4998206	169	180449	4892	France	2006	Human	C	C1	Cl3	Cl3.2	O25b:H4	fimH30		gyrA L83, N87	parC I80, V84
L03h	SAMEA7758323	5309315	133	173925	4920	France	2006	Human	C	C1	Cl3	Cl3.2	O25b:H4	fimH30		gyrA L83, N87	parC I80, V84
L04h	SAMEA7758324	5201866	162	191061	4824	France	2006	Human	B	B5	Cl2	Cl2.1	O25b:H4	fimH22		gyrA S83, N87	parC S80, E84
L05h	SAMEA7758325	5025562	60	218199	4651	France	2006	Human	B	B1	Cl2	Cl2.1	O25b:H4	fimH22		gyrA S83, N87	parC S80, E84
L06h	SAMEA7758326	5033930	136	180447	4836	France	2006	Human	C	C1	Cl3	Cl3.2	O25b:H4	fimH30		gyrA L83, N87	parC I80, V84
L102	SAMN09537399	5374577	149	191013	5158	France	2014	Human	C	C2	Cl3	Cl3.2	O25b:H4	fimH30	CTX-M-15	gyrA L83, N87	parC I80, V84
L106	SAMN09537400	5062160	177	55273	4682	France	2014	Human	C	C1	Cl3	Cl3.2	O25b:H4	fimH30	CTX-M-27	gyrA L83, N87	parC I80, V84
L111	SAMN09537402	5015861	113	123804	5033	France	2014	Human	C	C1	Cl3	Cl3.2	O25b:H4	fimH30	CTX-M-15	gyrA L83, N87	parC I80, V84
L114	SAMN09537403	5118496	199	58914	4936	France	2014	Human	C	C2	Cl3	Cl3.3	O25b:H4	fimH30	CTX-M-15	gyrA L83, N87	parC I80, V84
L134	SAMN09537404	5123620	82	167155	4737	France	2015	Human	C	C1	Cl3	Cl3.2	O25b:H4	fimH30	CTX-M-14	gyrA L83, N87	parC I80, V84
L136	SAMN09537405	5043610	86	181166	4704	France	2015	Human	C	C1	Cl3	Cl3.2	O25b:H4	fimH30	CTX-M-27	gyrA L83, N87	parC I80, V84
L14	SAMN09537385	5011366	106	176250	5119	France	2011	Human	C	C2	Cl3	Cl3.2	O25b:H4	fimH30	CTX-M-15	gyrA L83, N87	parC I80, V84
L140	SAMN09537406	5115065	87	149540	4742	France	2015	Human	C	C2	Cl3	Cl3.2	O25b:H4	fimH30	CTX-M-15	gyrA L83, N87	parC I80, V84
L146	SAMN09537407	5044339	83	191577	4680	France	2015	Human	C	C1	Cl3	Cl3.2	O25b:H4	fimH30	CTX-M-27	gyrA L83, N87	parC I80, V84
L148	SAMN09537408	5174720	114	145090	4794	France	2015	Human	C	C1	Cl3	Cl3.2	O25b:H4	fimH30	CTX-M-27	gyrA L83, N87	parC I80, V84
L149	SAMN09537409	5266660	103	198762	4814	France	2015	Human	C	C1	Cl3	Cl3.2	O25b:H4	fimH30	CTX-M-27	gyrA L83, N87	parC I80, V84
L155	SAMN09537411	5020313	90	192018	4717	France	2015	Human	C	C1	Cl3	Cl3.2	O25b:H4	fimH30	CTX-M-27	gyrA L83, N87	parC I80, V84
L166	SAMN09537413	5057383	85	184243	4680	France	2015	Human	C	C1	Cl3	Cl3.2	O25b:H4	fimH30	CTX-M-27	gyrA L83, N87	parC I80, V84
L169	SAMN09537415	5068658	100	158984	4801	France	2015	Human	C	C1	Cl3	Cl3.2	O25b:H4	fimH99	CTX-M-27	gyrA L83, N87	parC I80, V84
L173	SAMN09537417	5129653	101	99587	4740	France	2015	Human	C	C2	Cl3	Cl3.2	O25b:H4	fimH30	CTX-M-15	gyrA L83, N87	parC I80, V84
L175	SAMN09537418	5102195	94	191011	4854	France	2015	Human	C	C1	Cl3	Cl3.2	O25b:H4	fimH30	CTX-M-27	gyrA L83, N87	parC I80, V84
L176	SAMN09537419	5020858	83	218314	4979	France	2015	Human	C	C2	Cl3	Cl3.2	O25b:H4	fimH30	CTX-M-15	gyrA L83, N87	parC I80, V84
L31	SAMN09537388	5392520	104	148316	4732	France	2012	Human	C	C1	Cl3	Cl3.2	O25b:H4	fimH30	CTX-M-14	gyrA L83, N87	parC I80, V84
L36	SAMN09537389	5159788	149	89199	4742	France	2012	Human	C	C2	Cl3	Cl3.2	O25b:H4	fimH30	CTX-M-15	gyrA L83, N87	parC I80, V84
L58	SAMN09537393	5167841	90	164180	4742	France	2013	Human	C	C1	Cl3	Cl3.2	O25b:H4	fimH30	CTX-M-27	gyrA L83, N87	parC I80, V84
L89	SAMN09537398	4971361	164	112161	4825	France	2013	Human	C	C2	Cl3	Cl3.2	O25b:H4	fimH30	CTX-M-15	gyrA L83, N87	parC I80, V84
la_11242	SRR2970740	5141121	84	171117	4799	Laos	2008	Human	C	C1	Cl3	Cl3.2	O25b:H4	fimH30		gyrA L83, N87	parC I80, V84
la_11858	SRR2970728	5211644	88	186347	4631	Laos	2008	Human	A	A1	Cl1	Cl1.1	O16:H5	fimH41	CTX-M-14	gyrA S83, N87	parC S80, E84
la_13105	SRR2970762	5329937	98	136484	5122	Laos	2008	Human	C	C1	Cl3	Cl3.2	O25b:H4	fimH30	CTX-M-14	gyrA L83, N87	parC I80, V84
la_2266-2	SRR2970632	5094045	89	165063	4855	Laos	2006	Human	C	C1	Cl3	Cl3.2	O25b:H4	fimH30	CTX-M-14	gyrA L83, N87	parC I80, V84
la_5220-3	SRR2970717	5171965	87	124191	4885	Laos	2007	Human	C	C1	Cl3	Cl3.2	O25b:H4	fimH30	CTX-M-24	gyrA L83, N87	parC I80, V84
la_6169	SRR2970706	5128119	94	186343	4568	Laos	2007	Human	A	A1	Cl1	Cl1.1	O16:H5	fimH41	CTX-M-14	gyrA S83, N87	parC S80, E84
la_8507	SRR2970636	5030451	78	218645	4847	Laos	2007	Human	C	C1	Cl3	Cl3.2	O25b:H4	fimH30	CTX-M-27	gyrA L83, N87	parC I80, V84
M8	SRS1274389	5106383	98	225911	4918	China	2011	Human	C	C1	Cl3	Cl3.2	O25b:H4	fimH30		gyrA L83, N87	parC I80, V84
MB1074	SRS1274387	5402925	146	168676	5045	Ireland	2012	Human	I	I1	Cl3	Cl3.2	O25b:H4	fimH27		gyrA A83, N87	parC S80, E84
MB14972	SRS1274386	5035718	128	139904	4794	Ireland	2012	Human	C	C1	Cl3	Cl3.2	O25b:H4	fimH30		gyrA L83, N87	parC I80, V84
MB17684	SRS1274385	5316494	111	173703	4875	Ireland	2012	Human	C	C2	Cl3	Cl3.2	O25b:H4	fimH30		gyrA L83, N87	parC I80, V84
MB3298	SRS1274384	4878930	97	193550	4797	Ireland	2012	Human	B	B5	Cl3	Cl3.1	O25b:H4	fimH22		gyrA L83, N87	parC S80, E84
MB3323	SRS1274383	5239057	98	173698	4703	Ireland	2012	Human	C	C1	Cl3	Cl3.2	O25b:H4	fimH30		gyrA L83, N87	parC I80, V84
MH5800	SRR933535	5004474	66	210556	4792	Portugal	2005	Human	C	C2	Cl3	Cl3.2	O25b:H4	fimH30	CTX-M-15	gyrA L83, N87	parC I80, V84
MS2481	ERR161252	5163945	262	69625	5118	Australia	2007	Human	C	C2	Cl3	Cl3.2	O25b:H4	fimH30		gyrA L83, N87	parC I80, V84
MS2493	ERR161253	4973618	132	202839	4696	Australia	2007	Human	C	C1	Cl3	Cl3.2	O25b:H4	fimH30		gyrA L83, N87	parC I80, V84
MVAST014	SRR933393	5041596	114	165063	5038	USA	2010	Human	C	C1	Cl3	Cl3.2	O25b:H4	fimH30		gyrA L83, N87	parC I80, V84
MVAST020	SRR933395	4887294	66	218058	4527	USA	2010	Human	A	A1	Cl1	Cl1.1	O16:H5	fimH41		gyrA L83, N87	parC S80, E84
MVAST084	SRR933425	5143081	193	150495	4965	USA	2010	Human	C	C1	Cl3	Cl3.2	O25b:H4	fimH30		gyrA L83, N87	parC I80, V84
MVAST131	SRR933427	5130994	100	174196	4714	USA	2010	Human	C	C1	Cl3	Cl3.2	O25b:H4	fimH30		gyrA L83, N87	parC I80, V84
MVAST158	SRR933429	5106043	118	180842	4872	USA	2010	Human	C	C1	Cl3	Cl3.2	O25b:H4	fimH30		gyrA L83, N87	parC I80, V84
MVAST167	SRR933431	5130869	112	186343	4623	USA	2010	Human	A	A1	Cl1	Cl1.1	O16:H5	fimH41		gyrA L83, N87	parC S80, E84
MVAST179	SRR933433	5109947	93	186460	4727	USA	2010	Human	C	C1	Cl3	Cl3.2	O25b:H4	fimH30		gyrA L83, N87	parC I80, V84

NC11	SAMEA7758311	5139439	68	212809	4504	New Caledonia	2013	Human	A	A1	Cl1	Cl1.1	O16:H5	fimH41	CTX-M-3	gyrA L83, N87	parC I80, E84
NC117	SAMEA7758312	5283286	78	191796	4788	New Caledonia	2014	Human	A	A1	Cl1	Cl1.1	O16:H5	fimH41	CTX-M-27	gyrA L83, N87	parC S80, E84
NC36	SAMEA7758313	4943848	87	202057	4769	New Caledonia	2014	Human	A	A1	Cl1	Cl1.1	O16:H5	fimH41	CTX-M-27	gyrA L83, N87	parC S80, E84
NC51	SAMEA7758314	5205266	74	234851	4743	New Caledonia	2014	Human	A	A1	Cl1	Cl1.1	O16:H5	fimH41	CTX-M-27	gyrA L83, N87	parC S80, E84
NC57	SAMEA7758315	5198272	77	270056	4767	New Caledonia	2014	Human	A	A1	Cl1	Cl1.1	O16:H5	fimH41	CTX-M-27	gyrA L83, N87	parC S80, E84
NC81	SAMEA7758316	5112399	69	293540	4748	New Caledonia	2014	Human	A	A1	Cl1	Cl1.1	O16:H5	fimH41	CTX-M-27	gyrA L83, N87	parC S80, E84
ON28	DRR092874	5245859	86	393565	4785	Japan	2010	Human	B	B3	Cl2	Cl2.1	O25b:H4	fimH22		gyrA L83, N87	parC S80, E84
ONEC14	DRR051001	5093471	105	170190	4997	Japan	2006	Human	C	C2	Cl3	Cl3.2	O25b:H4	fimH30	CTX-M-15	gyrA L83, N87	parC I80, V84
ONEC31	DRR092865	5194727	80	248666	4575	Japan	2007	Human	A	A1	Cl1	Cl1.1	O16:H5	fimH41	CTX-M-14	gyrA S83, N87	parC S80, E84
ONEC9	DRR092872	5338473	77	196048	4919	Japan	2006	Human	C	C3	Cl3	Cl3.2	O25b:H4	fimH30		gyrA L83, N87	parC I80, V84
P146EC	ERR161313	5165580	251	142611	4892	Spain	2011	Human	C	C1	Cl3	Cl3.2	O25b:H4	fimH30		gyrA L83, N87	parC I80, V84
P33	SAMN14091247	5277838	70	158761	4794	USA	2015	Pig	B	B4	Cl3	Cl3.1	O25b:H4	fimH1531-like	CTX-M-15	gyrA S83, N87	parC S80, E84
P53EC	ERR161309	4888080	248	160389	4978	Spain	2011	Human	C	C1	Cl3	Cl3.2	O25b:H4	fimH30		gyrA L83, N87	parC I80, V84
P56EC	ERR161310	5091477	196	129478	4765	Spain	2011	Human	C	C1	Cl3	Cl3.2	O25b:H4	fimH30		gyrA L83, N87	parC I80, V84
PAP32	SAMEA7758317	5256787	95	186706	4895	Guadeloupe	2013	Human	C	C2	Cl3	Cl3.2	O25b:H4	fimH30	CTX-M-15	gyrA L83, N87	parC I80, V84
PAP33	SAMEA7758318	5368097	129	197387	5033	Guadeloupe	2013	Human	C	C2	Cl3	Cl3.2	O25b:H4	fimH30	CTX-M-15	gyrA L83, N87	parC I80, V84
PAP39	SAMEA7758319	5215322	120	159090	4871	Guadeloupe	2014	Human	C	C1	Cl3	Cl3.2	O25b:H4	fimH30	CTX-M-14	gyrA L83, N87	parC I80, V84
PAP41	SAMEA7758320	5214142	113	178563	5020	Guadeloupe	2013	Human	C	C2	Cl3	Cl3.3	O25b:H4	fimH30	CTX-M-15	gyrA L83, N87	parC I80, V84
QU090	SRR933423	5316482	76	191350	4519	Australia	2008	Human	A	A1	Cl1	Cl1.1	O16:H5	fimH41		gyrA L83, N87	parC I80, E84
QUC12	SRR933543	5170495	107	158993	4690	Australia	2008	Canine	A	A1	Cl1	Cl1.1	O16:H5	fimH41	CTX-M-14	gyrA L83, N87	parC S80, E84
R0007-199	ERS2017874	5402191	127	198323	4956	Tanzania	2014	Human	C	C2	Cl3	Cl3.2	O25b:H4	fimH30	CTX-M-15	gyrA L83, N87	parC I80, V84
R0007-210	ERS2017876	5228502	142	193809	5069	Tanzania	2014	Human	C	C2	Cl3	Cl3.2	O25b:H4	fimH30	CTX-M-15	gyrA L83, N87	parC I80, V84
R0009-587	ERS2017886	5145337	98	199488	4912	Tanzania	2014	Human	C	C2	Cl3	Cl3.2	O25b:H4	fimH30	CTX-M-15	gyrA L83, N87	parC I80, V84
RDK02-567B	ERS2017885	5093294	92	199488	4912	Tanzania	2015	Human	C	C2	Cl3	Cl3.2	O25b:H4	fimH30		gyrA L83, N87	parC I80, V84
RDK04-603	ERS2017887	5135006	120	193959	5008	Tanzania	2015	Human	C	C2	Cl3	Cl3.2	O25b:H4	fimH30	CTX-M-15	gyrA L83, N87	parC I80, V84
RDK40-70	ERS2017888	5087493	112	178652	4847	Tanzania	2013	Human	C	C2	Cl3	Cl3.2	O25b:H4	fimH30	CTX-M-15	gyrA L83, N87	parC I80, V84
RDK40-71E	ERS2017889	5104124	141	191062	5100	Tanzania	2013	Human	C	C2	Cl3	Cl3.2	O25b:H4	fimH30	CTX-M-15	gyrA L83, N87	parC I80, V84
RDK40-73	ERS2017890	5123158	197	191061	4887	Tanzania	2013	Human	C	C2	Cl3	Cl3.2	O25b:H4	fimH30	CTX-M-15	gyrA L83, N87	parC I80, V84
S100EC	ERR161263	5195953	175	192482	4817	Australia	2009	Human	C	C1	Cl3	Cl3.2	O25b:H4	fimH30	CTX-M-27	gyrA L83, N87	parC I80, V84
S102EC	ERR161265	5127822	199	143908	4792	Australia	2010	Human	C	C1	Cl3	Cl3.2	O25b:H4	fimH30	CTX-M-3	gyrA L83, N87	parC I80, V84
S104EC	ERR161267	5247006	108	157616	4806	Australia	2008	Human	B	B4	Cl3	Cl3.1	O25b:H4	fimH22		gyrA S83, N87	parC S80, E84
S105EC	ERR161268	5061451	108	154119	4768	Australia	2008	Human	B	B4	Cl3	Cl3.1	O25b:H4	fimH22		gyrA S83, N87	parC S80, E84
S108EC	ERR161271	5350821	148	190928	4751	Australia	2009	Human	C	C1	Cl3	Cl3.2	O25b:H4	fimH30	CTX-M-27	gyrA L83, N87	parC I80, V84
S10EC	ERR161237	5008480	175	121435	4801	UK	2009	Human	C	C2	Cl3	Cl3.2	O25b:H4	fimH30	CTX-M-15	gyrA L83, N87	parC I80, V84
S111EC	ERR161274	5064186	162	170494	4847	Australia	2009	Human	C	C2	Cl3	Cl3.2	O25b:H4	fimH30	CTX-M-15	gyrA L83, N87	parC I80, V84
S112EC	ERR161275	5295923	178	125967	4823	Australia	2009	Human	C	C1	Cl3	Cl3.2	O25b:H4	fimH30	CTX-M-3	gyrA L83, N87	parC I80, V84
S113EC	ERR161276	5297092	194	93628	4951	Australia	2009	Human	C	C2	Cl3	Cl3.2	O25b:H4	fimH30	CTX-M-15	gyrA L83, N87	parC I80, V84
S114EC	ERR161277	5033156	128	109625	4741	Australia	2011	Human	B	B4	Cl3	Cl3.1	O25b:H4	fimH22		gyrA S83, N87	parC S80, E84
S115EC	ERR161278	5203694	254	68136	5095	Australia	2011	Human	C	C2	Cl3	Cl3.2	O25b:H4	fimH30	CTX-M-15	gyrA L83, N87	parC I80, V84
S116EC	ERR161279	5294461	199	82834	4687	UK	2011	Human	C	C1	Cl3	Cl3.2	O25b:H4	fimH30		gyrA L83, N87	parC I80, V84
S117EC	ERR161280	5158740	127	185947	4750	UK	2011	Human	C	C1	Cl3	Cl3.2	O25b:H4	fimH30		gyrA L83, N87	parC I80, V84
S118EC	ERR161281	5090814	183	189466	5025	UK	2011	Human	C	C2	Cl3	Cl3.3	O25b:H4	fimH30	CTX-M-15	gyrA L83, N87	parC I80, V84
S119EC	ERR161282	5049941	176	189470	5021	UK	2011	Human	C	C2	Cl3	Cl3.3	O25b:H4	fimH30	CTX-M-15	gyrA L83, N87	parC I80, V84
S120EC	ERR161283	5407451	158	202804	4636	Canada	2009	Human	A	A1	Cl1	Cl1.1	O16:H5	fimH41	CTX-M-14	gyrA S83, N87	parC S80, E84
S121EC	ERR161284	5134192	251	56824	4896	Canada	2000	Human	C	C2	Cl3	Cl3.2	O25b:H4	fimH30	CTX-M-15	gyrA L83, N87	parC I80, V84
S124EC	ERR161287	5212395	231	123513	5003	Canada	2003	Human	C	C2	Cl3	Cl3.2	O25b:H4	fimH30	CTX-M-15	gyrA L83, N87	parC I80, V84
S126EC	ERR161289	5164140	190	89172	4857	Canada	2002	Human	C	C2	Cl3	Cl3.2	O25b:H4	fimH30	CTX-M-15	gyrA L83, N87	parC I80, V84
S127EC	ERR161290	5257153	124	190924	4780	Canada	2002	Human	C	C2	Cl3	Cl3.2	O25b:H4	fimH30	CTX-M-15	gyrA L83, N87	parC I80, V84
S128EC	ERR161291	5253636	168	77515	4723	Canada	2004	Human	B	B3	Cl2	Cl2.1	O25b:H4	fimH22		gyrA L83, N87	parC S80, E84
S129EC	ERR161292	5309712	283	93195	5125	Canada	2004	Human	C	C2	Cl3	Cl3.2	O25b:H4	fimH30	CTX-M-15	gyrA L83, N87	parC I80, V84
S12EC	ERR161239	5176188	166	119029	4805	UK	2009	Human	C	C2	Cl3	Cl3.2	O25b:H4	fimH30	CTX-M-15	gyrA L83, N87	parC I80, V84
S131EC	ERR161294	5134622	197	102668	4893	Canada	2002	Human	C	C2	Cl3	Cl3.2	O25b:H4	fimH30	CTX-M-15	gyrA L83, N87	parC I80, V84
S132EC	ERR161295	4978187	182	102316	4847	Canada	2005	Human	C	C2	Cl3	Cl3.2	O25b:H4	fimH30	CTX-M-15	gyrA L83, N87	parC I80, V84
S133EC	ERR161296	4868165	230	184251	4927	Canada	2005	Human	C	C2	Cl3	Cl3.2	O25b:H4	fimH30	CTX-M-15	gyrA L83, N87	parC I80, V84
S135EC	ERR161298	4906327	220	75949	4959	Canada	2005	Human	C	C1	Cl3	Cl3.2	O25b:H4	fimH30	CTX-M-14	gyrA L83, N87	parC I80, V84

S19EC	ERR161241	5118678	154	124042	5055	UK	2009	Human	B	B4	Cl3	Cl3.1	O25b:H4	fimH22		gyrA S83, N87	parC S80, E84
S21EC	ERR161242	5225797	236	242663	4863	UK	2009	Human	B	B4	Cl3	Cl3.1	O25b:H4	fimH22		gyrA S83, N87	parC S80, E84
S24EC	ERR161244	5039623	112	166751	4817	UK	2009	Human	B	B4	Cl3	Cl3.1	O25b:H4	fimH22		gyrA S83, N87	parC S80, E84
S250	SRR3657161	5240813	50	242276	4650	France	2006	Human	B	B1	Cl2	Cl2.1	O25b:H4	fimH22		gyrA S83, N87	parC S80, E84
S26EC	ERR161245	5343149	142	224808	4469	UK	2009	Human	A	A1	Cl1	Cl1.1	O16:H5	fimH41		gyrA S83, N87	parC S80, E84
S2EC	ERR161235	5283658	132	171487	4536	UK	2007	Human	A	A1	Cl1	Cl1.1	O16:H5	fimH41-like		gyrA S83, N87	parC S80, E84
S32EC	ERR161301	5102840	179	55902	4817	UK	2007	Human	B	B4	Cl3	Cl3.1	O25b:H4	fimH22		gyrA S83, N87	parC S80, E84
S34EC	ERR161247	5054554	198	107166	4901	UK	2009	Human	A	A1	Cl1	Cl1.1	O16:H5	fimH41		gyrA S83, N87	parC S80, E84
S5EC	ERR161236	5170763	140	179142	4688	UK	2007	Human	A	A1	Cl1	Cl1.1	O16:H5	fimH41		gyrA L83, N87	parC S80, E84
S6EC	ERR161299	5250090	139	143184	4937	UK	2007	Human	B	B4	Cl3	Cl3.1	O25b:H4	fimH22		gyrA S83, N87	parC S80, E84
S77EC	ERR161304	5360915	180	114885	5061	Australia	2010	Human	C	C2	Cl3	Cl3.2	O25b:H4		CTX-M-15	gyrA L83, N87	parC I80, V84
S79EC	ERR161305	5177228	169	136612	4963	Australia	2009	Human	B	B3	Cl2	Cl2.1	O25b:H4	fimH22		gyrA L83, N87	parC S80, K84
S92EC	ERR161255	5585941	142	173672	4786	New Zealand	2009	Human	C	C1	Cl3	Cl3.2	O25b:H4	fimH30		gyrA L83, N87	parC I80, V84
S94EC	ERR161257	5124132	151	187076	4676	New Zealand	2009	Human	A	A1	Cl1	Cl1.1	O16:H5	fimH41		gyrA S83, N87	parC S80, E84
S95EC	ERR161258	5089336	152	135875	4875	New Zealand	2009	Human	C	C1	Cl3	Cl3.2	O25b:H4	fimH30		gyrA L83, N87	parC I80, V84
S96EC	ERR161259	5056522	130	202750	4966	New Zealand	2010	Human	C	C2	Cl3	Cl3.2	O25b:H4	fimH30	CTX-M-15	gyrA L83, N87	parC I80, V84
S97EC	ERR161260	5085996	211	112009	5060	New Zealand	2010	Human	C	C2	Cl3	Cl3.2	O25b:H4	fimH30	CTX-M-15	gyrA L83, N87	parC I80, V84
S99EC	ERR161262	5229152	192	186832	4846	Australia	2009	Human	C	C2	Cl3	Cl3.2	O25b:H4	fimH30		gyrA L83, N87	parC I80, V84
SA186	SRR5937980	4717338	6	4828837	5259	Saudi Arabia	2006	Human	B	B3	Cl2	Cl2.3	O25b:H4	fimH22		gyrA L83, N87	parC S80, E84
SaT040	SRR933435	4937567	79	190080	4818	USA	2007	Human	B	B4	Cl3	Cl3.1	O25b:H4	fimH22		gyrA S83, N87	parC S80, E84
SaT049	SRR933437	5153384	100	165063	4821	USA	2007	Human	C	C1	Cl3	Cl3.2	O25b:H4	fimH30		gyrA L83, N87	parC I80, V84
SaT142	SRR933439	5190249	97	268759	4726	USA	2003	Human	B	B5	Cl3	Cl3.1	O25b:H4	fimH22		gyrA S83, N87	parC S80, E84
SaT158	SRR933441	5188459	107	186342	4789	USA	2003	Human	C	C1	Cl3	Cl3.2	O25b:H4	fimH30		gyrA L83, N87	parC I80, V84
SCB34	SAMN02712194	5174566	81	205944	4973	USA	2013	Human	C	C2	Cl3	Cl3.3	O25b:H4	fimH30		gyrA L83, N87	parC I80, V84
SE15	NC_013654	4984890	1	4717338	4296	Canada	2015	Human	A	A1	Cl1	Cl1.1	O16:H5	fimH41		gyrA S83, N87	parC S80, E84
SI11	DRR092878	5151944	103	159896	4588	Japan	2011	Human	A	A1	Cl1	Cl1.1	O16:H5	fimH41	CTX-M-14	gyrA L83, N87	parC S80, E84
TN03	SRS5403688	5102069	106	203999	4768	France	2002	Human	C	C2	Cl3	Cl3.2	O25b:H4	fimH30	CTX-M-15	gyrA L83, N87	parC I80, V84
U004	SRR933443	5094591	77	191083	4805	USA	2010	Human	C	C2	Cl3	Cl3.2	O25b:H4	fimH35	CTX-M-15	gyrA L83, N87	parC I80, V84
U024	SRR933445	5064840	91	133545	4744	USA	2010	Human	C	C1	Cl3	Cl3.2	O25b:H4	fimH30		gyrA L83, N87	parC I80, V84
U054	SRR933447	5066860	108	186995	4731	USA	2010	Human	A	A1	Cl1	Cl1.1	O16:H5	fimH89		gyrA S83, N87	parC S80, E84
U12	SRS1274382	5094184	50	284289	4778	UK	2012	Human	B	B4	Cl3	Cl3.1	O25b:H4	fimH22		gyrA S83, N87	parC S80, E84
U2	SRS1274381	5196627	97	163729	4920	UK	2012	Human	C	C2	Cl3	Cl3.2	O25b:H4	fimH30	CTX-M-15	gyrA L83, N87	parC I80, V84
U44	SRS1274380	5174542	100	173082	4869	UK	2012	Human	C	C2	Cl3	Cl3.2	O25b:H4	fimH30		gyrA L83, N87	parC I80, V84
U5	SRS1274379	5259272	126	136502	4946	UK	2012	Human	B	B4	Cl3	Cl3.1	O25b:H4	fimH22		gyrA S83, N87	parC S80, E84
U79	SAMN04457315	5291500	125	172941	4989	UK	2012	Human	C	C2	Cl3	Cl3.2	O25b:H4	fimH30	CTX-M-15	gyrA L83, N87	parC I80, V84
U92	SRS1274377	5271587	139	155293	4965	UK	2012	Human	I	I4	Cl3	Cl3.2	O25b:H4	fimH30		gyrA S83, N87	parC S80, E84
uk_17A7A	SRR2970700	5290693	76	158986	5023	UK	2009	Human	C	C2	Cl3	Cl3.2	O25b:H4	fimH30	CTX-M-15	gyrA L83, N87	parC I80, V84
uk_18A18K	SRR2970696	5104923	85	158840	4777	UK	2009	Human	C	C2	Cl3	Cl3.2	O25b:H4	fimH30	CTX-M-15	gyrA L83, N87	parC I80, V84
uk_18A33A	SRR2970701	5205482	97	169995	4908	UK	2009	Human	C	C2	Cl3	Cl3.2	O25b:H4	fimH30	CTX-M-15	gyrA L83, N87	parC I80, V84
uk_18B18D	SRR2970720	5232956	80	165059	4918	UK	2010	Human	C	C2	Cl3	Cl3.2	O25b:H4	fimH30	CTX-M-15	gyrA L83, N87	parC I80, V84
uk_18B21F	SRR2970705	5109405	86	159642	4780	UK	2010	Human	C	C2	Cl3	Cl3.2	O25b:H4	fimH30	CTX-M-15	gyrA L83, N87	parC I80, V84
uk_18B29L	SRR2970718	5125274	85	159642	4720	UK	2010	Human	B	B5	Cl2	Cl2.1	O25b:H4	fimH22		gyrA L83, N87	parC S80, E84
uk_18B30B	SRR2970714	5089134	53	259940	4916	UK	2010	Human	C	C2	Cl3	Cl3.2	O25b:H4	fimH30	CTX-M-15	gyrA L83, N87	parC I80, V84
uk_18C16C	SRR2970702	5240829	79	178431	5039	UK	2010	Human	C	C2	Cl3	Cl3.2	O25b:H4	fimH30	CTX-M-15	gyrA L83, N87	parC I80, V84
uk_18C22I	SRR2970709	5293783	111	161494	4790	UK	2010	Human	C	C2	Cl3	Cl3.2	O25b:H4	fimH30	CTX-M-15	gyrA L83, N87	parC I80, V84
uk_18C23A	SRR2970704	5113213	83	172642	4933	UK	2010	Human	C	C2	Cl3	Cl3.2	O25b:H4	fimH30	CTX-M-15	gyrA L83, N87	parC I80, V84
uk_18C4F	SRR2970713	5236255	76	166689	4908	UK	2010	Human	C	C2	Cl3	Cl3.2	O25b:H4	fimH30	CTX-M-15	gyrA L83, N87	parC I80, V84
uk_19A21D	SRR2970703	5235170	81	191083	4927	UK	2010	Human	C	C2	Cl3	Cl3.2	O25b:H4	fimH30	CTX-M-15	gyrA L83, N87	parC I80, V84
uk_19A8G	SRR2970716	5237112	78	165063	4827	UK	2010	Human	B	B4	Cl3	Cl3.1	O25b:H4	fimH22		gyrA S83, N87	parC S80, E84
uk_19B17I	SRR2970708	5151706	79	204114	4940	UK	2010	Human	C	C2	Cl3	Cl3.2	O25b:H4	fimH30	CTX-M-15	gyrA L83, N87	parC I80, V84
uk_19B19L	SRR2970691	5239672	68	172642	4984	UK	2010	Human	C	C2	Cl3	Cl3.2	O25b:H4	fimH99	CTX-M-15	gyrA L83, N87	parC I80, V84
uk_19B22L	SRR2970692	5247383	93	165063	4990	UK	2010	Human	C	C2	Cl3	Cl3.2	O25b:H4	fimH30		gyrA L83, N87	parC I80, V84
uk_7C26H	SRR2970694	5270645	61	166795	4911	UK	2008	Human	C	C2	Cl3	Cl3.2	O25b:H4	fimH30	CTX-M-15	gyrA L83, N87	parC I80, V84
uk_8A19D	SRR2970693	5227711	83	172642	4899	UK	2008	Human	C	C2	Cl3	Cl3.2	O25b:H4	fimH30	CTX-M-15	gyrA L83, N87	parC I80, V84

uk_8A9B	SRR2970690	5198062	83	159643	4602	UK	2008	Human	A	A1	Cl1	Cl1.1	O16:H5	fimH41	CTX-M-14	gyrA S83, N87	parC S80, E84
uk_P26250	SRR2970657	5005275	113	188399	4922	UK	2005	Human	C	C2	Cl3	Cl3.2	O25b:H4	fimH30	CTX-M-15	gyrA L83, N87	parC I80, V84
uk_P34091	SRR2970634	5209166	68	173798	4929	UK	2005	Human	C	C2	Cl3	Cl3.2	O25b:H4	fimH30	CTX-M-15	gyrA L83, N87	parC I80, V84
uk_P46212	SRR2970646	5221946	88	212750	4838	UK	2005	Human	C	C2	Cl3	Cl3.2	O25b:H4	fimH30	CTX-M-15	gyrA L83, N87	parC I80, V84
uk_UKUEC2	SRR2970668	5153588	79	165059	4801	UK	2010	Human	C	C2	Cl3	Cl3.2	O25b:H4	fimH30	CTX-M-15	gyrA L83, N87	parC I80, V84
UR40	SAMN03354200	5267762	90	216212	4974	Italia	2006	Human	C	C2	Cl3	Cl3.2	O25b:H4	fimH30	CTX-M-15	gyrA L83, N87	parC I80, V84
USA14	DRS034173	5217628	93	191127	4943	USA	2008	Human	C	C1	Cl3	Cl3.2	O25b:H4	fimH30	CTX-M-14	gyrA L83, N87	parC I80, V84
WCE208	SRS1274374	5161145	149	137211	4862	China	2006	Human	C	C1	Cl3	Cl3.2	O25b:H4	fimH30	CTX-M-14	gyrA L83, N87	parC I80, V84
WCE233	SRS1274373	5123154	82	192431	4814	China	2006	Human	C	C1	Cl3	Cl3.2	O25b:H4	fimH30	CTX-M-3	gyrA L83, N87	parC I80, V84
WCE266	SRS1274375	5227638	141	187015	4954	China	2005	Human	C	C1	Cl3	Cl3.2	O25b:H4	fimH30	CTX-M-14	gyrA L83, N87	parC I80, V84
WCE296	SRS1274372	5223957	142	166481	4947	China	2005	Human	C	C1	Cl3	Cl3.2	O25b:H4	fimH30	CTX-M-14	gyrA L83, N87	parC I80, V84
WCE307	SAMN04457316	5336717	197	170841	5048	China	2005	Human	C	C1	Cl3	Cl3.2	O25b:H4	fimH30	CTX-M-65	gyrA L83, N87	parC I80, V84
ZH071	SRR933451	5071903	130	125931	4804	Canada	2002	Human	B	B4	Cl3	Cl3.1	O25b:H4	fimH35		gyrA S83, N87	parC S80, E84
ZRUEC11	SRS3361170	5310985	94	191062	5011	Pakistan	2013	Human	C	C2	Cl3	Cl3.2	O25b:H4	fimH30	CTX-M-15	gyrA L83, N87	parC I80, V84
