# Supplementary material for: Host Colonization as a Major Evolutionary Force Favoring the Diversity and the Emergence of the Worldwide Multidrug-Resistant Escherichia coli ST131
Source: mBio. 2021 Aug 24;12(4):e01451-21. doi: 10.1128/mBio.01451-21 (PMC8406181; doi:10.1128/mBio.01451-21)
Supplement: TABLE S2 [file mbio.01451-21-st002.pdf]

Supplementary Table 2: Cluster/subcluster features with the frequency of subclades, serogroups, fimH alleles, major hosts, discriminant VFs and resistance mechanisms. The key values are indicated in bold.

Cluster (size)		C1 (69)	C2 (175)					C3 (552)		
Subcluster (size)		C1.1 (69)	C2.1 (69)	C2.2 (22)	C2.3 (23)	C2.4 (32)	C2.5 (29)	C3.1 (56)	C3.2 (434)	C3.3 (62)
Subclade	A1 (n=69)	**100.0%**	0.0%	0.0%	0.0%	0.0%	0.0%	0.0%	0.0%	0.0%
	B1 (n=9)	0.0%	**10.1%**	0.0%	4.3%	0.0%	0.0%	1.8%	0.0%	0.0%
	B2 (n=7)	0.0%	8.7%	0.0%	0.0%	0.0%	0.0%	1.8%	0.0%	0.0%
	B3 (n=135)	0.0%	**56.5%**	**100.0%**	**82.6%**	**81.3%**	**100.0%**	0.0%	0.0%	0.0%
	B4 (n=39)	0.0%	0.0%	0.0%	0.0%	0.0%	0.0%	**67.9%**	0.2%	0.0%
	B5 (n=40)	0.0%	**24.6%**	0.0%	**13.0%**	**18.8%**	0.0%	**25.0%**	0.0%	0.0%
	C1 (n=195)	0.0%	0.0%	0.0%	0.0%	0.0%	0.0%	0.0%	**44.9%**	0.0%
	C2 (n=273)	0.0%	0.0%	0.0%	0.0%	0.0%	0.0%	0.0%	**48.6%**	**100.0%**
	C3 (n=7)	0.0%	0.0%	0.0%	0.0%	0.0%	0.0%	0.0%	1.6%	0.0%
	I1 (n=9)	0.0%	0.0%	0.0%	0.0%	0.0%	0.0%	1.8%	1.6%	0.0%
	I2 (n=6)	0.0%	0.0%	0.0%	0.0%	0.0%	0.0%	0.0%	1.4%	0.0%
	I3 (n=4)	0.0%	0.0%	0.0%	0.0%	0.0%	0.0%	1.8%	0.7%	0.0%
	I4 (n=4)	0.0%	0.0%	0.0%	0.0%	0.0%	0.0%	0.0%	0.9%	0.0%
Serogroup	O16:H5 (n=67)	**97.1%**	0.0%	0.0%	0.0%	0.0%	0.0%	0.0%	0.0%	0.0%
	O25b:H4 (n=717)	2.9%	**95.7%**	**100.0%**	**100.0%**	**100.0%**	**89.7%**	**98.2%**	**99.1%**	**98.4%**
	O25b:H17 (n=1)	0.0%	0.0%	0.0%	0.0%	0.0%	0.0%	0.0%	0.2%	0.0%
	O2:H4 (n=2)	0.0%	0.0%	0.0%	0.0%	0.0%	0.0%	1.8%	0.0%	1.6%
	O62:H4 (n=1)	0.0%	1.4%	0.0%	0.0%	0.0%	0.0%	0.0%	0.0%	0.0%
FimH allele	fimH22 (n=217)	0.0%	**100.0%**	**100.0%**	**100.0%**	**100.0%**	**100.0%**	**81.3%**	0.7%	0.0%
	fimH27 (n=7)	0.0%	0.0%	0.0%	0.0%	0.0%	0.0%	2.1%	1.4%	0.0%
	fimH30 (n=469)	0.0%	0.0%	0.0%	0.0%	0.0%	0.0%	12.5%	**93.7%**	**100.0%**
	fimH35 (n=14)	0.0%	0.0%	0.0%	0.0%	0.0%	0.0%	4.2%	2.8%	0.0%
	fimH41 (n=67)	**100.0%**	0.0%	0.0%	0.0%	0.0%	0.0%	0.0%	0.0%	0.0%
	fimH54 (n=6)	0.0%	0.0%	0.0%	0.0%	0.0%	0.0%	0.0%	1.4%	0.0%
Host	Avian (n=139)	0.0%	**59.4%**	**72.7%**	**77.3%**	**100.0%**	**100.0%**	3.6%	0.5%	0.0%
	Canine (n=100)	14.5%	7.2%	0.0%	0.0%	0.0%	0.0%	14.3%	12.7%	**36.1%**
	Human (n=506)	**79.7%**	30.4%	22.7%	9.1%	0.0%	0.0%	**73.2%**	**79.5%**	**60.7%**
Resistance mechanism	blaCTX-M-15	**23.88%**	1.52%	0.00%	0.00%	0.00%	0.00%	3.64%	**48.47%**	**72.13%**
	gyrA D87N	**26.87%**	3.03%	0.00%	0.00%	0.00%	0.00%	5.45%	**93.88%**	**91.80%**
	parC S80I	**25.37%**	4.55%	0.00%	0.00%	0.00%	0.00%	5.45%	**93.88%**	**91.80%**
	mph(A)	**52.24%**	3.03%	0.00%	4.76%	3.13%	0.00%	14.55%	**55.53%**	**55.74%**
	blaOXA-1	6.0%	1.5%	0.0%	0.0%	0.0%	0.0%	5.5%	**38.1%**	**72.1%**
	parC E84V	8.96%	3.03%	0.00%	0.00%	0.00%	0.00%	5.45%	**93.88%**	**91.80%**
	aac(6')-Ib-cr	5.97%	1.52%	0.00%	0.00%	0.00%	0.00%	3.64%	**38.12%**	**72.13%**
	aac(3)-IIa	4.48%	3.03%	0.00%	4.76%	0.00%	0.00%	3.64%	**22.12%**	**47.54%**
	catB4	6.0%	1.5%	0.0%	0.0%	0.0%	0.0%	3.6%	**34.4%**	**72.1%**
	gyrA S83L	**70.15%**	**30.30%**	**47.62%**	**33.33%**	0.00%	0.00%	14.55%	**96.00%**	**95.08%**
	blaCTX-M-27	**14.93%**	0.00%	0.00%	0.00%	0.00%	0.00%	1.82%	**16.47%**	8.20%
	blaCTX-M-14	**29.85%**	3.03%	0.00%	0.00%	0.00%	0.00%	0.00%	7.53%	6.56%
	aph(3')-Ia	7.5%	10.6%	0.0%	**23.8%**	**71.9%**	**100.0%**	3.6%	2.8%	0.0%
	tetB	11.9%	10.6%	0.0%	**42.9%**	**84.4%**	**100.0%**	9.1%	5.2%	0.0%
	tetD	7.5%	10.6%	0.0%	**33.3%**	**75.0%**	**93.1%**	9.1%	4.7%	0.0%
	aac(3)-IId	**37.3%**	7.6%	0.0%	**33.3%**	**65.6%**	**58.6%**	5.5%	18.4%	6.6%
	aac(3)-IV	0.0%	1.5%	0.0%	9.5%	12.5%	**82.8%**	0.0%	0.0%	0.0%
	aph(4)-Ia	0.0%	1.5%	0.0%	9.5%	12.5%	**82.8%**	0.0%	0.0%	0.0%
	blaTEM-1	**64.2%**	**33.3%**	**14.3%**	**52.4%**	**84.4%**	**96.6%**	**30.9%**	**50.1%**	**19.7%**
	tetA	**43.3%**	**34.8%**	**19.0%**	**33.3%**	**6.3%**	**6.9%**	**12.7%**	**57.9%**	**70.5%**
	sul1	**50.7%**	**19.7%**	4.8%	**66.7%**	**75.0%**	**62.1%**	**21.8%**	**59.1%**	**59.0%**
	sul2	**49.3%**	**19.7%**	4.8%	**38.1%**	**68.8%**	**100.0%**	**21.8%**	**34.4%**	**21.3%**
	aph(3'')-Ib	**47.8%**	**12.1%**	0.0%	**38.1%**	**59.4%**	**75.9%**	**18.2%**	**32.5%**	**14.8%**
	aph(6)-Id	**49.3%**	**18.2%**	0.0%	**52.4%**	**46.9%**	**51.7%**	**20.0%**	**32.5%**	**16.4%**
	dfrA17	**47.8%**	4.5%	0.0%	0.0%	**65.6%**	**58.6%**	7.3%	**53.9%**	**50.8%**
	aadA5	**47.8%**	4.5%	0.0%	0.0%	**65.6%**	**58.6%**	5.5%	**53.4%**	**52.5%**
Virulence factor	cjr	**66.7%**	0.0%	0.0%	0.0%	0.0%	0.0%	**50.0%**	**37.1%**	**56.5%**
	sat	**55.1%**	0.0%	0.0%	0.0%	0.0%	0.0%	**71.4%**	**94.2%**	**100.0%**
	papX	**76.8%**	13.8%	0.0%	4.3%	0.0%	0.0%	**76.8%**	**93.7%**	**91.9%**
	Yqi pili	**82.6%**	41.5%	0.0%	17.4%	18.8%	0.0%	**100.0%**	**99.3%**	**100.0%**
	fbp	**98.6%**	43.1%	0.0%	17.4%	18.8%	0.0%	**98.2%**	**99.8%**	**100.0%**
	senB	**71.0%**	0.0%	0.0%	0.0%	0.0%	0.0%	**53.6%**	**40.1%**	**54.8%**
	iha	**82.6%**	9.2%	**100.0%**	26.1%	0.0%	0.0%	**71.4%**	**93.0%**	**100.0%**
	papI	**85.5%**	33.8%	0.0%	26.1%	**71.9%**	**100.0%**	**78.6%**	**95.6%**	**100.0%**
	Dr pili	**29.0%**	0.0%	0.0%	0.0%	0.0%	0.0%	**48.2%**	**18.6%**	0.0%
	kpsMT-K5	1.4%	0.0%	0.0%	0.0%	0.0%	0.0%	**67.9%**	**45.7%**	1.6%
	fec	1.4%	9.2%	0.0%	4.3%	3.1%	0.0%	23.2%	**92.5%**	**100.0%**
	P pili	17.4%	30.8%	0.0%	13.0%	3.1%	0.0%	10.7%	17.0%	**98.4%**
	F17-like pili	15.9%	7.7%	0.0%	4.3%	0.0%	0.0%	12.5%	11.7%	**98.4%**
	cdiAB	13.0%	18.5%	0.0%	8.7%	9.4%	0.0%	12.5%	9.6%	**98.4%**
	α-hly	14.5%	7.7%	0.0%	4.3%	0.0%	0.0%	10.7%	7.9%	**87.1%**
	kpsMT-K2	5.8%	0.0%	0.0%	0.0%	0.0%	0.0%	5.4%	12.6%	**95.2%**
	cnf-1	14.5%	7.7%	0.0%	4.3%	0.0%	0.0%	7.1%	7.0%	**88.7%**
	tia	26.1%	**50.8%**	**85.7%**	**52.2%**	**87.5%**	**100.0%**	21.4%	22.6%	**100.0%**
	ibeABC	0.0%	**29.7%**	**9.6%**	**10.0%**	**14.6%**	**13.2%**	**20.5%**	1.4%	0.0%
	kpsMT-K1	2.9%	**55.4%**	**95.2%**	**82.6%**	**75.0%**	**100.0%**	0.0%	1.4%	0.0%
	neu K1	0.0%	**49.2%**	**95.2%**	**60.9%**	**34.4%**	**31.0%**	0.0%	0.0%	0.0%
	sslE	4.3%	**81.5%**	**100.0%**	**95.7%**	**81.3%**	**65.5%**	19.6%	0.7%	0.0%
	hlyF	1.4%	**90.8%**	**100.0%**	**100.0%**	**87.5%**	**100.0%**	1.8%	1.9%	0.0%
	ets	1.4%	**75.4%**	**95.2%**	**91.3%**	**96.9%**	**100.0%**	1.8%	1.4%	0.0%
	salmochelin	1%	**92%**	**100%**	**100%**	**88%**	**100%**	9%	2%	0%
	eit	13.0%	**35.4%**	**95.2%**	**78.3%**	**75.0%**	**93.1%**	7.1%	3.0%	1.6%
	tsh (2 copies)	0.0%	**35.4%**	**76.2%**	**82.6%**	**53.1%**	**55.2%**	0.0%	0.0%	0.0%
	Pix pili	0.0%	**41.5%**	**95.2%**	**73.9%**	0.0%	0.0%	0.0%	0.0%	0.0%
	aatA	0.0%	6.2%	**95.2%**	17.4%	3.1%	0.0%	0.0%	0.0%	0.0%
	astA	7.2%	1.5%	0.0%	**34.8%**	6.3%	6.9%	1.8%	2.1%	3.2%
	CS31	1.4%	10.8%	0.0%	0.0%	**75.0%**	**100.0%**	0.0%	0.0%	0.0%
	K88 pili	0.0%	10.8%	0.0%	0.0%	**68.8%**	**82.8%**	0.0%	0.0%	0.0%
	lateral flagella	0.0%	**86.2%**	**100.0%**	**95.7%**	**100.0%**	**100.0%**	**92.9%**	**100.0%**	**100.0%**
	bor	1.4%	**95.4%**	**100.0%**	**100.0%**	**100.0%**	**100.0%**	**98.2%**	**97.2%**	**100.0%**
	cirA	**97.1%**	**92.3%**	0.0%	**82.6%**	**96.9%**	**100.0%**	**100.0%**	**97.0%**	**91.9%**
